# Western visitors at the *Blätterhöhle* (city of Hagen, southern Westphalia) during the Younger Dryas? A new final palaeolithic assemblage type in western Germany

**DOI:** 10.1371/journal.pone.0284479

**Published:** 2023-05-03

**Authors:** Michael Baales, Wolfgang Heuschen, Martin Kehl, Annika Manz, Nadine Nolde, Daniel Riemenschneider, Holger Rittweger, Jörg Orschiedt

**Affiliations:** 1 Department Olpe, LWL-Archaeology for Westphalia (State Office for Archaeology Westphalia), Olpe, Germany; 2 Institute of Pre- and Protohistory, Department of Archaeological Sciences, Ruhr-University Bochum, Bochum, Germany; 3 City Office for Preservation of Monuments and Archaeology, Hagen, Germany; 4 Institute of Geography, University of Cologne, Cologne, Germany; 5 Institute of Pre- and Protohistory, University of Cologne, Cologne, Germany; 6 MObiles LAndschaftsMUseum, Office for Landscape- and Palaeo-Ecology, Waldbrunn, Germany; 7 State Office for Preservation of Monuments and Archaeology Saxony-Anhalt, Halle (Saale), Germany; 8 Institute of Prehistoric Archaeology, Department of History and Cultural Studies, Freie Universität Berlin, Berlin, Germany; Universita degli Studi di Ferrara, ITALY

## Abstract

Until now, it was considered certain that the last reindeer hunters of the Ahrensburgian (tanged point groups) existed exclusively in northwestern Central Europe during the Younger Dryas Cold Period (~ Greenland Stadial 1). The excavations carried out since 2006 on the forecourt (*Vorplatz*) of the small *Blätterhöhle* in Hagen on the northern edge of the Sauerland uplands of southern Westphalia (North Rhine-Westphalia, western Germany) have now changed this view. Beneath a surprisingly extensive sequence of Mesolithic find horizons, Pleistocene sediments could be reached whose excavations yielded a Final Palaeolithic lithic ensemble of the Younger Dryas, unusual for the region and beyond. It is characterised by numerous backed lithic projectile points of high variability. Comparisons suggest a typological-technological connection with the Western European Laborian / Late Laborian. Neither in the nearer nor in the wider surroundings has a comparable lithic find ensemble been found so far. In addition, there is a lack of clear evidence for the reindeer in the fauna. Surprisingly, the vast majority of radiocarbon dates of bones and charcoals from the investigated archaeological horizon of the Final Pleistocene proved to be significantly older than expected from their stratigraphic position. This phenomenon has not yet been clarified.

## Introduction: The late upper and final palaeolithic and early mesolithic in West Germany and adjacent regions—A brief overview

Since 2006, excavations inside and in front of the *Blätterhöhle* cave near Hagen on the northern edge of the South Westphalian *Sauerland* uplands (North Rhine-Westphalia, western Germany) have provided many new insights into different periods of the Stone Age for North Rhine-Westphalia and beyond:

1^st^: numerous Mesolithic and Neolithic human remains have been recovered from the narrow cave, the analysis of which provides numerous insights into human behaviour in the respective epochs
2^nd^: in front of the cave entrance a sequence of Mesolithic layers was uncovered, which is considered as a reference stratigraphy for North Rhine-Westphalia and beyond
3^rd^: finally, below the Mesolithic horizons an exceptional find assemblage dating to the very end of the preceding Weichselian ice age was recovered which surprisingly expands our knowledge of human behaviour and short-term environmental changes during the Younger Dryas period in the region and beyond.

This paper presents the first results on the scientific and archaeological analyses concerning the last point.

**Fig 1:**
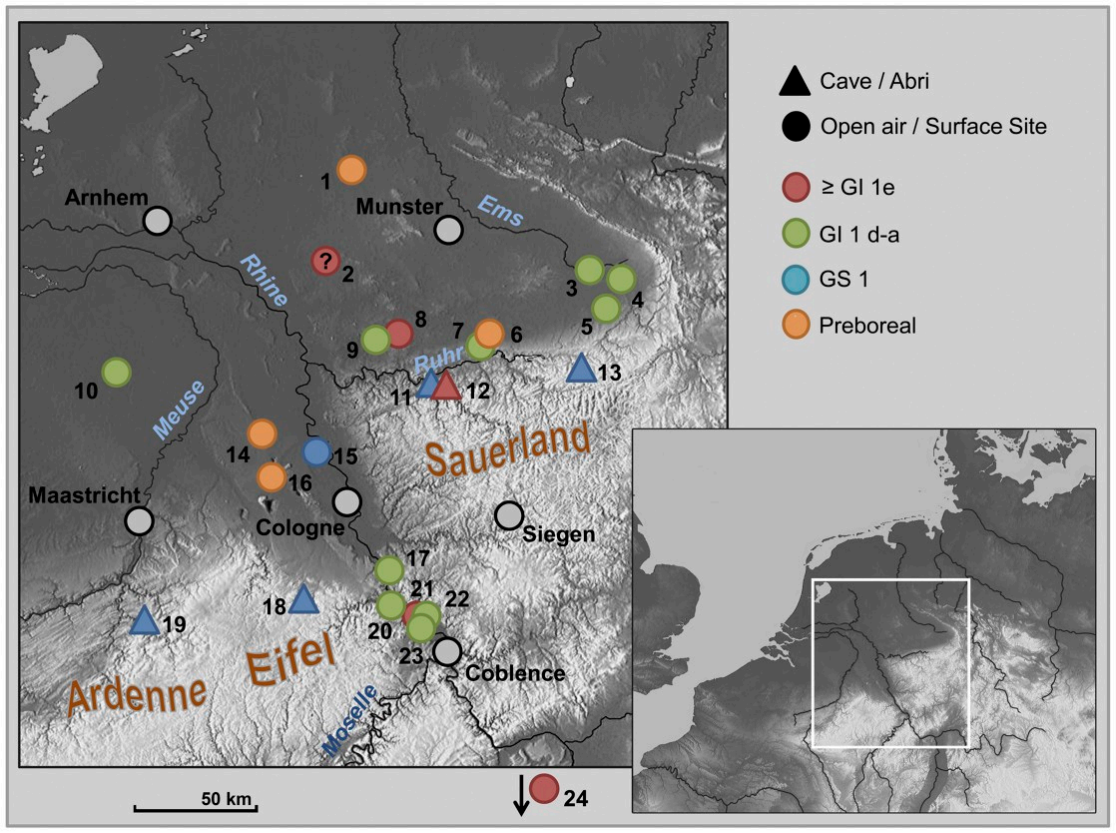
Late upper palaeolithic, final palaeolithic and preboreal (early mesolithic) sites in Rhineland, Westphalia and neighbouring regions in NW Europe mentioned or discussed in the text. – 1 Heek (distr. Borken, Munsterland Bucht, northern Westphalia); 2 Borken-Gemenkrückling (distr. Borken, Munsterland Bucht, northern Westphalia); 3 Rietberg (distr. Gütersloh, Munsterland Bucht, eastern Westphalia); 4 Paderborn-Sande (distr. Paderborn, Munsterland Bucht, eastern Westphalia); 5 Salzkotten-Thüle (distr. Paderborn, Munsterland Bucht, eastern Westphalia); 6 Werl-Büderich (distr. Soest, Soest Börde & Munsterland Bucht, southern Westphalia); 7 Fröndenberg-Schelk (distr. Unna, Haarstrang, southern Westphalia); 8 Castrop-Rauxel (distr. Recklinghausen, Ruhr region, northern Westphalia); 9 Herne (Ruhr region, southern Westphalia); 10 Geldrop (prov. North-Brabant, The Netherlands); 11 *Blätterhöhle* (Hagen, northern Sauerland, southern Westphalia); 12 *Oeger Höhle* (Hagen, northern Sauerland, southern Westphalia); 3 *Hohler Stein* (Rüthen-Kallenhardt, distr. Soest, northern Sauerland, southern Westphalia); 14 Mönchengladbach-Geneicken (Lower Rhine plain, Rhineland); 15 Dormagen-Nievenheim (distr. Rhein-Neuss, Lower Rhine plain, Rhineland); 16 Bedburg-Königshoven (distr. Rhein-Erft, Lower Rhine plain, Rhineland); 17 Bonn-Oberkassel (Rhineland); 18 *Kartstein* (Mechernich, North Eifel uplands, Rhineland); 19 Remouchamps (Aywaille, prov. de Liège, northern Ardenne, southern Belgium); 20 Bad Breisig (distr. Ahrweiler, northern Rhineland-Palatinate); 21 Gönnersdorf (Neuwied, Neuwied Basin, northern Rhineland-Palatinate); 22 Irlich (Neuwied, Neuwied Basin, northern Rhineland-Palatinate); 23 Andernach-Martinsberg (distr. Mayen-Koblenz, Neuwied Basin, northern Rhineland-Palatinate); 24 Fußgönheim (Maxdorf, distr. Rhein-Pfalz, southern Rhineland-Palatinate).–Geochronological classification of the Palaeolithic sites in the oxygen isotope stages according to Greenland ice cores.–Graphic: map basis = maps-for-free.com released under Creative Commons CC0; realisation by LWL-AfW Olpe/M. Baales & A. Müller.

**Fig 2:**
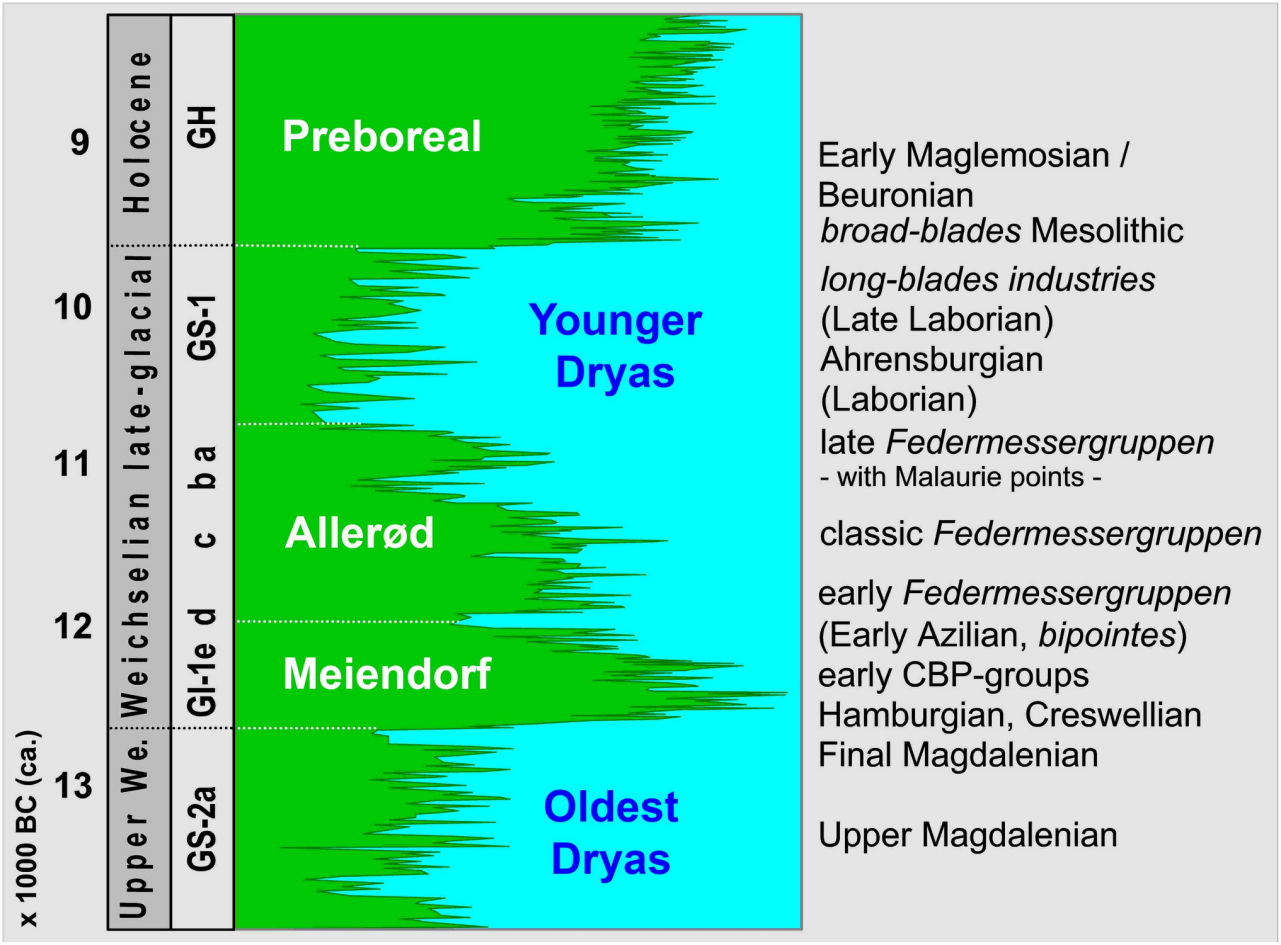
Geochronological classification of late upper palaeolithic, final palaeolithic and early mesolithic technocomplexes in NW Europe (in brackets: Exclusively French technocomplexes) against the background of the NGRIP ice core chronology [cf. 6]. –Graphic: map basis = Geobasis data of the NRW state and municipalities, Land NRW (2021)–Licence dl-de/zero-2-0 to LWL; realisation by LWL-AfW Olpe/M. Baales.

In principle–as will be outlined below–, the overall development of the late Upper and Final Palaeolithic techno-complexes for the Final Pleistocene Greenland Interstadial (GI) 1 and the following Greenland Stadial (GS) 1 in western Germany–northern Rhineland, Westphalia and adjacent regions (Fig 1)–is known (Fig 2), but the details are seldom better grasped due to only less well-dated sites. This is in clear contrast to France, for example, where extensive excavations and analysis projects have produced a fairly detailed picture for some regions over the last few decades [1–5]. At least there are some interesting parallels that can be drawn for our region.

Typologically, only a few lithic assemblages in the northern Rhineland and adjacent regions (Fig 1) can be classified as a Final Magdalenian with shouldered/angle-backed lithic projectile points (Fußgönheim, west of Ludwigshafen am Rhein, Upper Rhine Plain, southern Rhineland-Palatinate [7, 8]; Petershagen-Frille, north of Minden, Wesertal, eastern Westphalia [9, pp. 110]), which in France and Switzerland can be dated to the transition to and early GI-1e (Meiendorf-Interstadial [2, 10, 11, cf. 12–14]). However, there is no geochronometrically dated site at hand in our region. A conventional radiocarbon age on a bulked sample of female/subadult reindeer antler remains (Oeger Höhle, Hagen-Hohenlimburg, northern *Sauerland* upland, southern Westphalia) of about 13 kyr cal BC (*kilo-years calibrated before Christ*) is of little help [15]. Slightly younger are ^14^C dates of faunal remains from Gönnersdorf (Neuwied Basin, Central Rhineland, northern Rhineland-Palatinate), which can be attributed to a younger phase of use [16]; however, lithic artefacts certainly dating to this period are absent [cf. 17]. Further to the North, the late Upper Palaeolithic Hamburgian with the younger facies of the Havelte group dates to this period and later, and is first characterised by shouldered projectile points [12]. It remains to be seen whether a comparable point from the west of the Munsterland Basin near Borken-Gemenkrückling (northern Westphalia) should be classified here [9, pp. 110, but cf. 13].

A basal reindeer antler fragment from Castrop-Rauxel on the southern edge of the Munsterland Basin belongs to the time of the Havelte Group of the Hamburgian, the beam of which was separated by ringing around its entire circumference above the bez tine; this is an rare indication that around 12,2 kyr cal BC reindeer were still part of the fauna (and exploited by human groups) in northern Westphalia [15]. This is interesting because only a few dozen kilometres to the south-west (Fig 1), faunal remains of the same age refer to a completely different biotope: In connection with the human remains from Bonn-Oberkassel (northern Rhineland), bones of elk, red deer and brown bear were recovered in addition to the dog (*Canis familiaris*), which refer to a fundamentally different environmental situation [18, 19]. However, the temporal resolution of the finds and the findings from the ^14^C-method are too low to be able to draw any further conclusions.

Some human remains from Irlich (Neuwied Basin, northern Rhineland-Palatinate) date to the same period as Bonn-Oberkassel, with which a jewellery tooth pendant and two stone artefacts of little significance were recovered [20].

In contrast to some regions in France, where the oldest post-Magdalenian inventories (*bipointe* horizon) from GI-1e are quite common [2, 4], comparably significant inventories are missing in western Germany. If we look at south-west Germany, the ^14^C and biostratigraphically well-dated assemblages from the *Burghöhle* Dietfurt and the basal layers of the *Zigeunerfels* (both: upper Danube, Baden-Württemberg [21, 22]) should be taken into account, which, based on the lithic artefact morphology, are reminiscent of the *bipointe*-Creswellian complex showing NW-Europe [23]. Obviously there is a western influence to Central Europe in the GI-1e.

The East Westphalian site of Rietberg is a chronologically somewhat more recent fixed point for eastern North Rhine-Westphalia [13, 24]. Several back point ensembles have been excavated here whose radiocarbon age and biostratigraphic attribution point to the oldest, birch dominanted stage of the Allerød-Interstadial (GI-1c; “Bølling” according to [25]) around 11,7 kyr cal BC. In fact, some of the backed points still show echoes of *bipointes* not uncommon in French inventories of the same time (cf. Conty, Somme [3]).

According to their ^14^C-ages, only slightly older are two fragments of giant deer (*Megaloceros*) antlers from the southern edge of the Munsterland Basin (Herne and Paderborn-Sande) in Westphalia, the antlers of which in turn were separated by ring notches [15]; as they are single finds, they unfortunately cannot be evaluated further in terms of cultural stratigraphy. This also applies to some relatively old ^14^C-dates from Andernach-Martinsberg (Neuwied Basin, Central Rhineland, northern Rhineland-Palatinate [26, cf. 27]), chronologically placed between the Late Magdalenian there around 13,8 kyr cal BC and the younger Allerød period of the *Federmessergruppen* (pre-11 kyr cal BC, at the time of the Laacher See eruption; see below) from the same locality. Among the lithic artefacts–I.e. the projectile points–however, significant, typologically certainly older elements [cf. 28], such as those described for Rietberg, are absent here.

The classic *Federmessergruppen* and the boreal fauna of that time are highly evident in western Germany (Rhineland and adjacent regions), especially from the Central Rhineland Neuwied Basin (cf. Fig 1); the fact that these, as well as settlement sites and sites with relics from this period, are well recorded is mainly due to their preservation below huge tephra deposits erupted by the adjacent Laacher See volcano around 11 kyr cal BC [29]; these results have been extensively published [27, 30–37].

Stratigraphically younger than the Laacher See Tephras (LST) is a *Federmessergruppen* inventory (dated approx. 10,8 kyr cal BC) that could be examined a little north of the Neuwied Basin. The site of Bad Breisig (in the district of Ahrweiler, northern Rhineland-Palatinate) can be compared well with the inventories below the LST, but a few back points are present which bases were also intentionally retouched [38–40]. This, together with a straight back, is a feature found in Malaurie points known from France. The oldest specimens are dated to the end of the Allerød (see e.g. the recent *Federmessergruppen* horizon at Le Closeau near Paris [41]) while Malaurie points became typical there in the following GS-1 / Younger Dryas (*Laborien*/*Laborien ancien* [42–44]).

The Malaurie points, which can also be detected elsewhere in the wider area (e.g. in Salzkotten-Thüle in eastern Westphalia and Fröndenberg in southern Westphalia [24, 45, 46]) are again evidence of a western influence in our region, as they are a formative element of the final Allerød / Younger Dryas time slice in neighbouring western Europe.

In respect of the following cold-climatic Younger Dryas period (GS-1), an influence from northern regions is commonly found in our region, if one considers the formation of the tanged point groups in the North European Plain. The oldest ^14^C-dates on charcoal from a fireplace in Alt Duvenstedt LA 121 and 123 (Schleswig-Holstein, northern Germany) could indicate a use of the region by groups with small tanged points, which are typical within the Ahrensburgian towards the end of GI-1a [47, 48]. However, there is little data relating to this.

Surprisingly of a similar age is a reindeer bone with cut marks from the Grotte de Remouchamps in the northern Belgian Ardennes; here, too, the Ahrensburgian is amply documented with numerous lithic artefacts and reindeer as hunting prey being typical [49]. It is, however, an Oxford AMS measurement (OxA-4191) from the early 1990s. It might be necessary to check whether this date can be considered reliable according to today’s criteria; in addition, two other bone data from the same Oxford series date 4–500 radiocarbon years younger and are thus the same age as another ^14^C-age from a different laboratory [47, 48, 50].

Whether the relatively old charcoal dates for Ahrensburgian inventories from the Dutch Geldrop southeast of Eindhoven are reliable (especially with regard to more recent data on burned bones [51]) must be critically questioned [cf. 47, 51].

The sites with Ahrensburgian tanged points are generally dated to GS-1 (Fig 2), although radiometrically reliably dated sites are not common. Very similar ages of 10,1–9,9 kyr cal BC could be obtained from two reindeer remains from southern Westphalia, which indicate the use of the well-known *Hohler Stein* near Kallenhardt (Rüthen, Soest district, on the northern edge of the *Sauerland* uplands) in the second half of GS-1 [15]. Some of the data for the *Kartstein* in the northern *Eifel* uplands some 70 kms southwest of Cologne (between Remouchamps in the west and the *Hohler Stein* in the east and thus also on the northern edge of the low mountain range) are even younger [47], although there are definitely problematic ages here especially according to today’s measurement and sample standards and only one reindeer bone age from Oxford can be considered reasonably reliable for dating human activities at *Kartstein* (around 10,1–9,8 kyr cal BC [52]).

Thus, on the left and right of the Rhine on the northern edge of the low mountain range zone, two Ahrensburgian assemblages with numerous reindeer remains and other typical cold-age faunal elements are present [9, 47, 49, 53], which date to the second half of the Younger Dryas period. This current picture is also not contradicted by the relatively flat calibration curve for the younger section of the Younger Dryas period [cf. 27].

To the west of the distribution area with typical inventories of the Ahrensburgian, lithic inventories were identified about 40 years ago, which are particularly worthy of note due to large blades, a bipolar knapping method for large cores, oversized blades with severe edge damage (bruised blades) and often heterogeneous or variable projectile point types [2, 54, 55]. These *long-blade industries*–or the Belloisien–are derived from the Ahrensburgian (and recently re-assigned as Épi-Ahrensbourgien by French colleagues [56]). There are in fact large blades with characteristic bruised edges present within assemblages containing typical Ahrensburgian points (e.g. Teltwisch 2, *Ahrensburger Tunneltal*, Schleswig-Holstein [48]). However, the distribution area of the *long-blade industries* is significantly larger (from Denmark to Central France and southern England) than the classic distribution area of the Ahrensburgian disregarding, for example, the Scottish and southern English tanged points of Ahrensburgian type [57, 58]. The wide distribution of the *long-blade* inventories thus seems to reflect a north/northeast influence towards west/southwest Europe (France and southern England).

In the area examined here, there are several inventories, albeit not scientifically dated, which can be placed alongside the *long-blade industries* (especially from the northern Münsterland Basin, northern Westphalia [9, 59, 60]). The only assemblage dated is the one discovered in 2016 from Dormagen-Nievenheim located on the Lower Rhine (south of Düsseldorf). Numerous large blades and blade implements have been discovered here, together with a projectile inventory of simple points (so called Zohnhoven points with and without base retouching) associated with such inventories elsewhere as well. The find sediment could be dated to 11,5 ± 0.9 ky BP using OSL, a fireplace using birch charcoal was ^14^C-dated to about 10,1–9,5 kyr cal BC [60, 61], and therefore roughly the same period or tending to be somewhat younger than the reindeer bones dated from the *Hohler Stein* Ahrensburgian.

These results tend to suggest that in our region–and presumably also in other regions with inventories of the Ahrensburgian–during (the second half?) GS-1 different groups with different stone artefact traditions existed [60]. This could be used as an argument to challenge the common view that the *long-blade industries* are only to be placed chronologically at the end of the Younger Dryas period or even in the earliest Preboreal. They are possibly more deeply rooted in the GS-1 / Younger Dryas and therefore should not be classified as “Epi-Ahrensburgian”, as has recently been attempted [cf. 56].

In this context it is of interest that the inventory of Dormagen-Nievenheim shows a raw material spectrum that is spatially dispersed according to its respective occurrences and this indicates a significant range of movement of the group. Western European flint varieties are represented (in addition to regional Meuse gravel flint, the Rijckholt and Lousberg types from the southern Netherlands and the city of Aachen respectively), which implies distances of 40–80 km. Erratic Baltic flint from the regional moraine deposits is dominant. The distinctive red Heligoland flint with small yellow inclusions, which is represented in the assemblage of some 2350 lithics at 1.4%, is particularly striking [60, 61, cf. 62]. Apart from an end scraper also small pieces of waste were found which therefore does not suggest that individual items were exchanged with other groups. Heligoland is about 350 km north-east of Dormagen as the crow flies. This once again illustrates the enormous areas that Late and Final Pleistocene people migrated through or used, perhaps seasonally.

Based on a few sites, including some that have only recently been discovered, the characteristics of the initial, postglacial (early Preboreal; Fig 2) Mesolithic can currently be understood quite well for our area, much better than was the case a few years ago [59]. The recently specified age of Bedburg-Königshoven (Rhein-Erft-Kreis, Lower Rhineland) with its typical early Holocene fauna proves that the inventory including the two well-known antler headdresses can be dated to the very beginning of the Preboreal (ca. 9,6 kyr cal BC [60, 63]); the environmental changes–from a largely open steppe landscape to closed, albeit sparse forest landscapes–were therefore already well advanced. The hunting find of an aurochs cow in Geneicken (town of Mönchengladbach), also located on the Lower Rhine in the valley of the Niers, is of a similar age to Bedburg-Königshoven, as well as small lithic assemblages excavated adjacent to the bone scatter in the immediate vicinity of fireplaces. One of the inventories is characterised by base-retouched microliths [60, 64]. Such an early appearance of such projectile forms was unexpected; they are considered to be an influence from southern regions and are characteristic of the early Mesolithic Beuronian-Coincy tradition, which at its full extent spread from the Paris Basin to southern Poland [65, cf. 66].

Decades ago, Martin Street [67] associated the stone artefacts from Bedburg-Königshoven with the English so-called *broad-blade Mesolithic* [cf. 68] which can be understood as a variant (or just as a conceptual synonym) of the early Maglemosian developing in the North European Plain [69]. In Bedburg-Königshoven basally retouched points are absent (with only three projectiles perhaps being more of a coincidence, since the actual settlement site had already been destroyed before the excavation in 1988/89), but such projectile points are also absent in other early, Preboreal inventories. Instead, various simple or so-called Zonhoven points and triangles are evident in Heek and Werl-Büderich in the Munsterland Basin of Westphalia [68]. The inventory of Werl-Büderich (in the district of Soest) is associated with typical Holocene fauna and has been dated to 9,4–9,3 kyr cal BC and compares well with Middle Preboreal inventories from Friesack in Brandenburg (northwest of Berlin [59]). Some of the simple microliths from the basal early Holocene sedimentary sequence of the entrance area of the *Blätterhöhle* (the site is presented in detail below) may date to the same period [59].

This brief overview shows that the cultural-historical sequence in Rhineland and Westphalia against the background of the well-known (northwest) European development can basically be traced quite well, but in some details it can still be insufficiently traced [60]. Ultimately, there is a lack of informative stratigraphic squences with several find horizons that make the development directly comprehensible. In most cases, only few lithic assemblages or finds are available, which are more or less well provided with scientific data.

It can be ascertained that for the region considered here, typo-technological influences from different regions can be traced for the entire Final Pleistocene and initial Holocene. Apart from the "Younger Dryas hiatus" with the Ahrensburgian, which is primarily a phenomenon of northern Central Europe, constant south-western influences can be observed in this period. But, surprisingly, such influences are now also suggested for the “hiatus” by the new results from the entrance area of the *Blätterhöhle* presented below which are able to change our view so far on the Younger Dryas archaeological record in western Germany.

## *Blätterhöhle*—A cave and rock shelter site in South Westphalia

### Setting

The South Westphalian highlands south of the Ruhr, of which the *Sauerland* uplands are a part (Fig 1), belong to the northern edge of the Rhenish Massif. Here there are geological hollows with Middle Devonian reef limestone and dolomite deposits, in which numerous caves can be found. Such a west-east trending dolomite deposit is located on the northern edge of the so called *Remscheider saddle* between the citys of Hagen—Iserlohn—Hemer, which then bends to the south and forms the well-known cave landscape of the *Hönnetal* (valley of the Hönne river) near Balve (Märkischer Kreis district [70]).

In addition to well-known large caves (such as the *Dechenhöhle* near Iserlohn, which is rich in stalactites), numerous small caves and crevices are known, some of which have not yet been explored in their entirety [71]. Almost every year, new, mostly smaller caves can be added to the list.

### Research history

**Fig 3:**
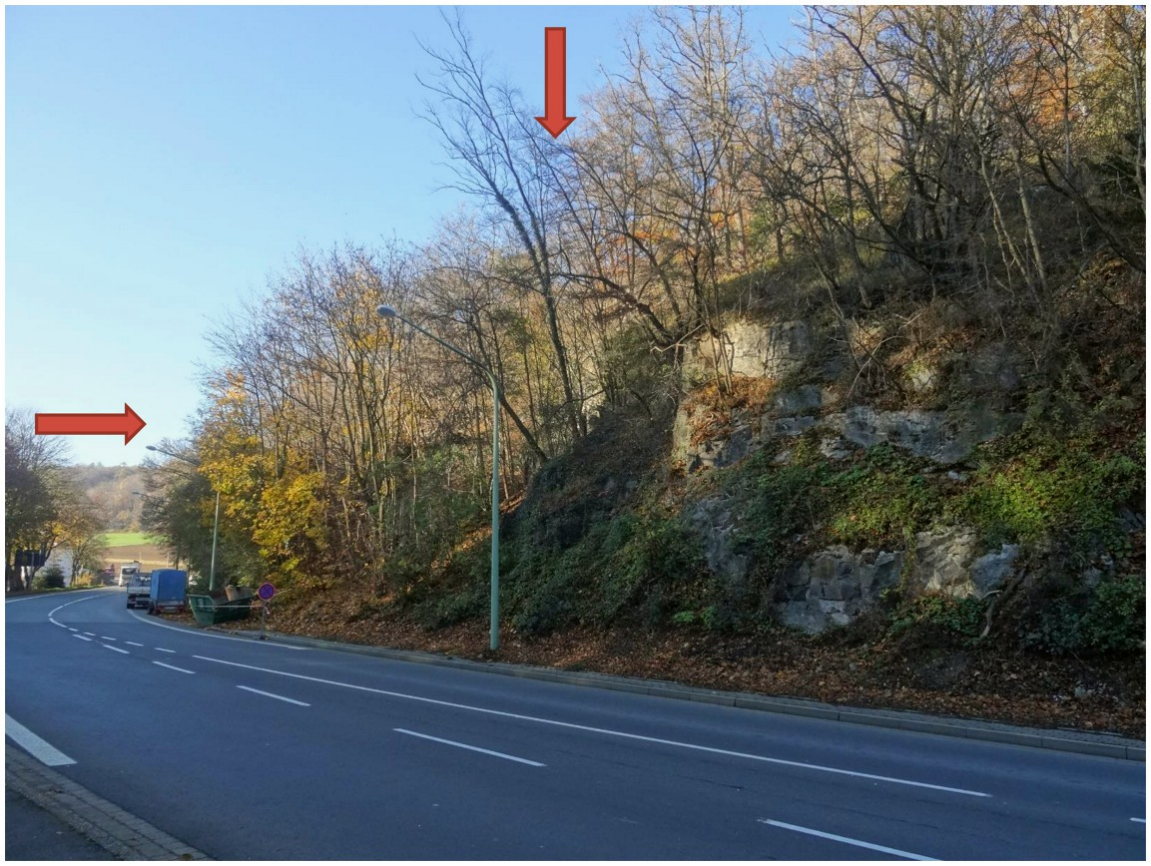
View (from SE) of the slope into the valley of the *Milchenbach* with the *Blätterhöhle* (arrows) at the southern end of the *Weissenstein*. –Photo: LWL-AfW Olpe/M. Baales.

**Fig 4:**
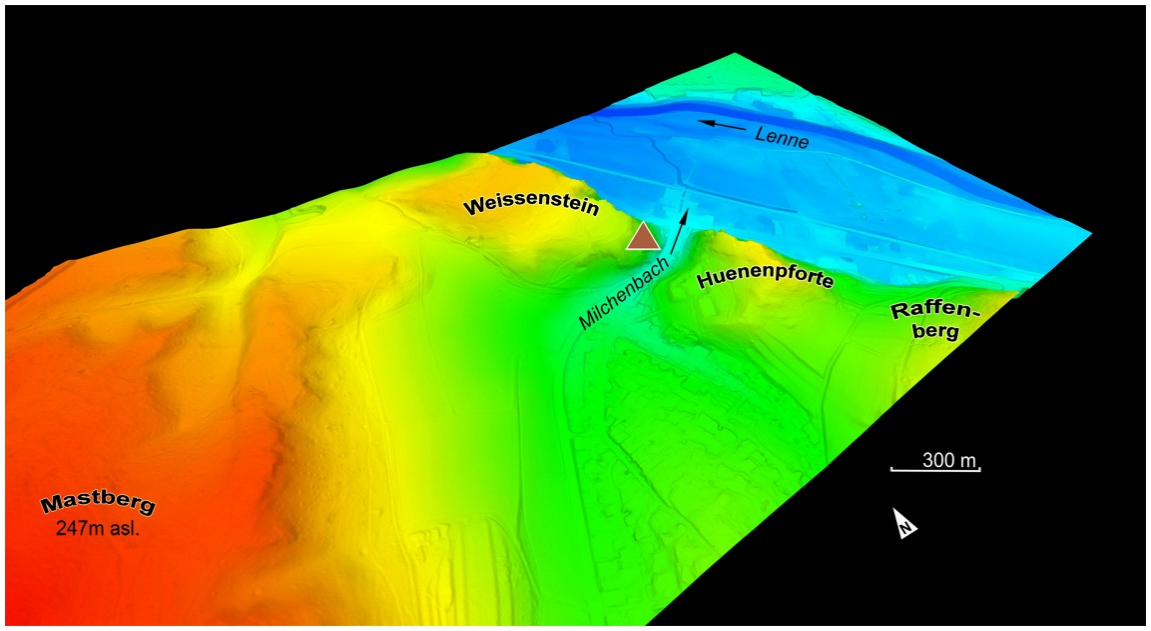
Location of the *Blätterhöhle* in the processed DEM terrain model. –Graphics and editing: LWL-AfW/I. Pfeffer & A. Müller; map basis: Geobasis data of the NRW state and municipalities © Geobasis NRW 2015.

**Fig 5:**
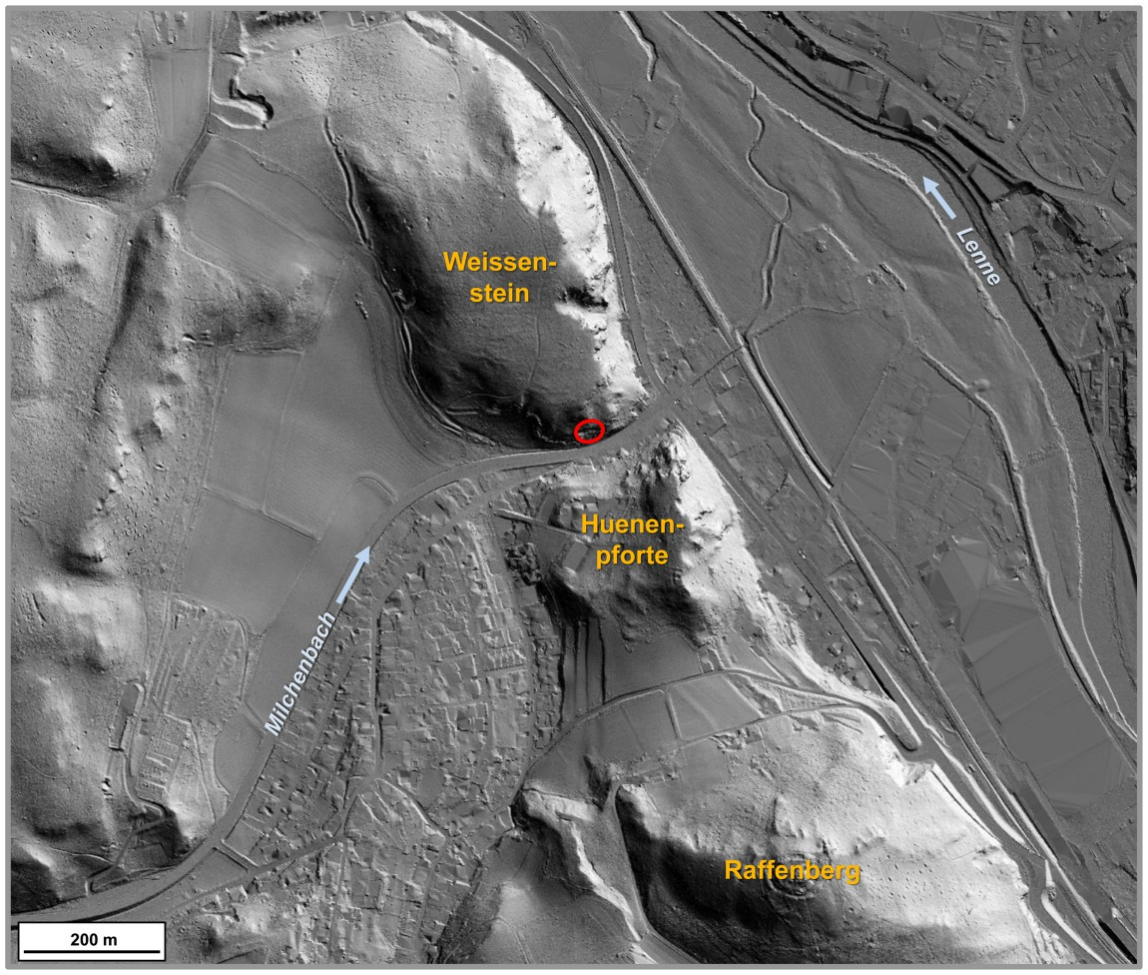
Situation of the *Blätterhöhle* on the western edge of the valley of the Lenne, DEM orthographic view. –Graphic: Geobasis data of the NRW state and municipalities, Land NRW [2021]–licence dl-de/zero-2-0; realisation LWL-AfW Olpe/M. Baales.

In 1983 the speleologists of the Kluterthöhle e.V. working group (Ennepe-Ruhr district, South Westphalia) discovered a small crevasse (now cave entrance 1) at the foot of the southern *Weissenstein* (Figs 3–5) a dolomite massif which was later christened *Blätterhöhle* (Leaf Cave) due to its dense foliage cover. It lies a little to the west of the confluence of the *Milchenbach* and the *Lenne*, which, coming from the south-east, flows into the Ruhr a little further on. In 2004, the city of Hagen commissioned the working group Kluterthöhle e.V. with further research into this cave in order to gain insights into the hydrological conditions in the *Weissenstein*. In doing so, particular attention was to be paid to archaeological finds, as agreed in advance with the Archaeological Heritage Service. Over the Easter period of 2004, the narrow cave passage was widened and a not inconsiderable amount of sediment was removed. The numerous human bones uncovered in the process, however, gave no reason to stop the undertaking, so that the continued work finally created a 15 m deep crawl space and numerous finds were brought out.

In retrospect, it turned out that the human remains uncovered in the process, which can be assigned to two epochs (Early Mesolithic and Late Neolithic), come from sediments that were mainly disturbed by numerous badgers. This was the result of the subsequent archaeological excavations, in which human remains could be recovered from cave sediments that had been subjected to intense bioturbation. The human remains of both Stone Age periods are interpreted as remains of primary burials; indications of secondary burial practices (e.g. in the form of cut marks on the bones) are missing [72–75]. Several studies have been published on the extensive analyses of the human remains, which also played a role in aDNA projects and revealed new aspects of the prehistoric way of life in the region during the two periods covered by human remains [76–80]. In the following sections, on the other hand, the focus is on the most recent results of the excavations begun in 2006 on the entrance area below cave entrance 1 which comprised a rock shelter (abri) situation during the Final Pleistocene and Early Holocene [cf. 75].

### The entrance area (*Vorplatz*) to the cave

**Fig 6:**
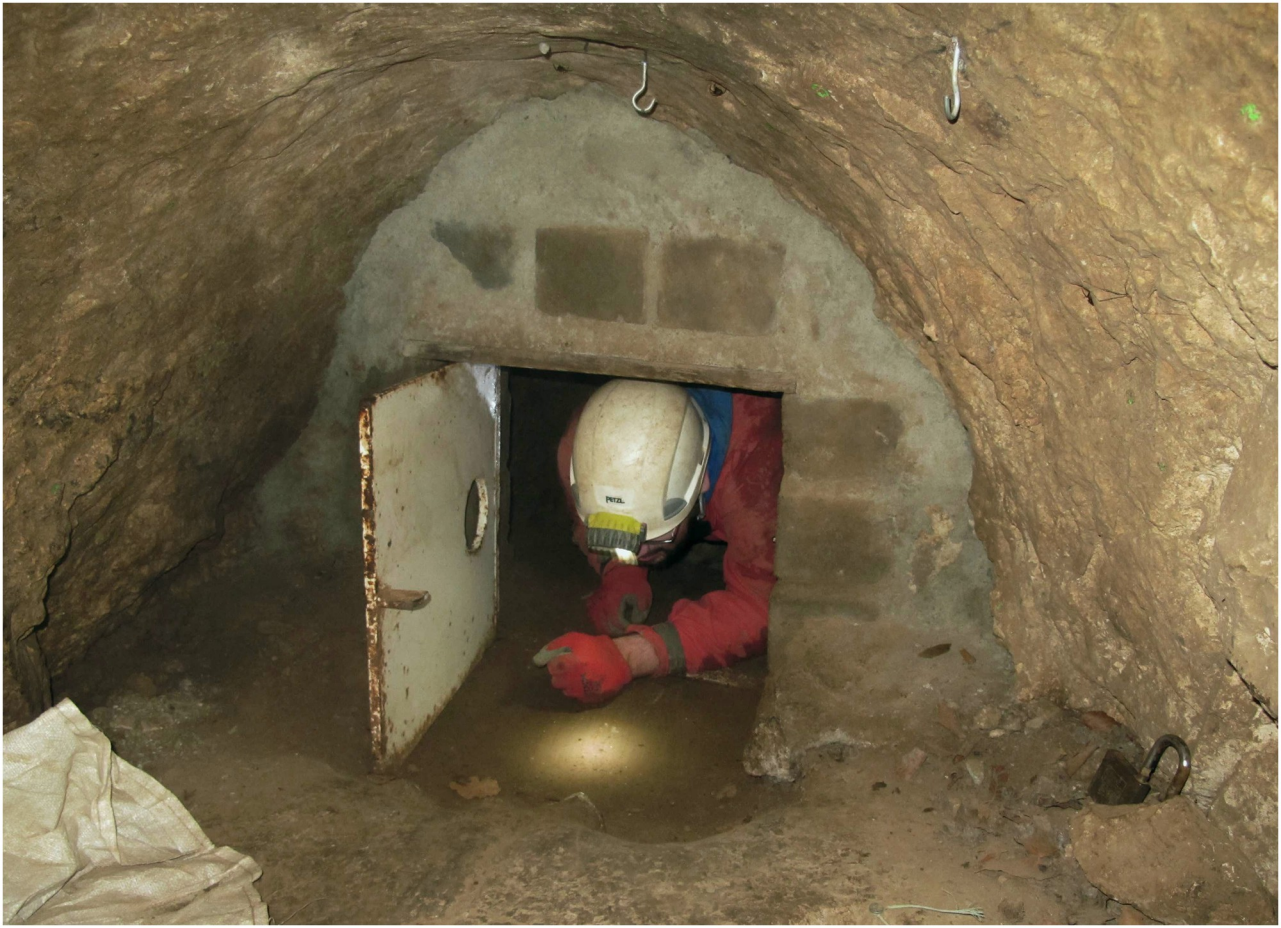
*Blätterhöhle* entrance situation with the protective gate in place. –Photo: City of Hagen/W.Heuschen.

**Fig 7:**
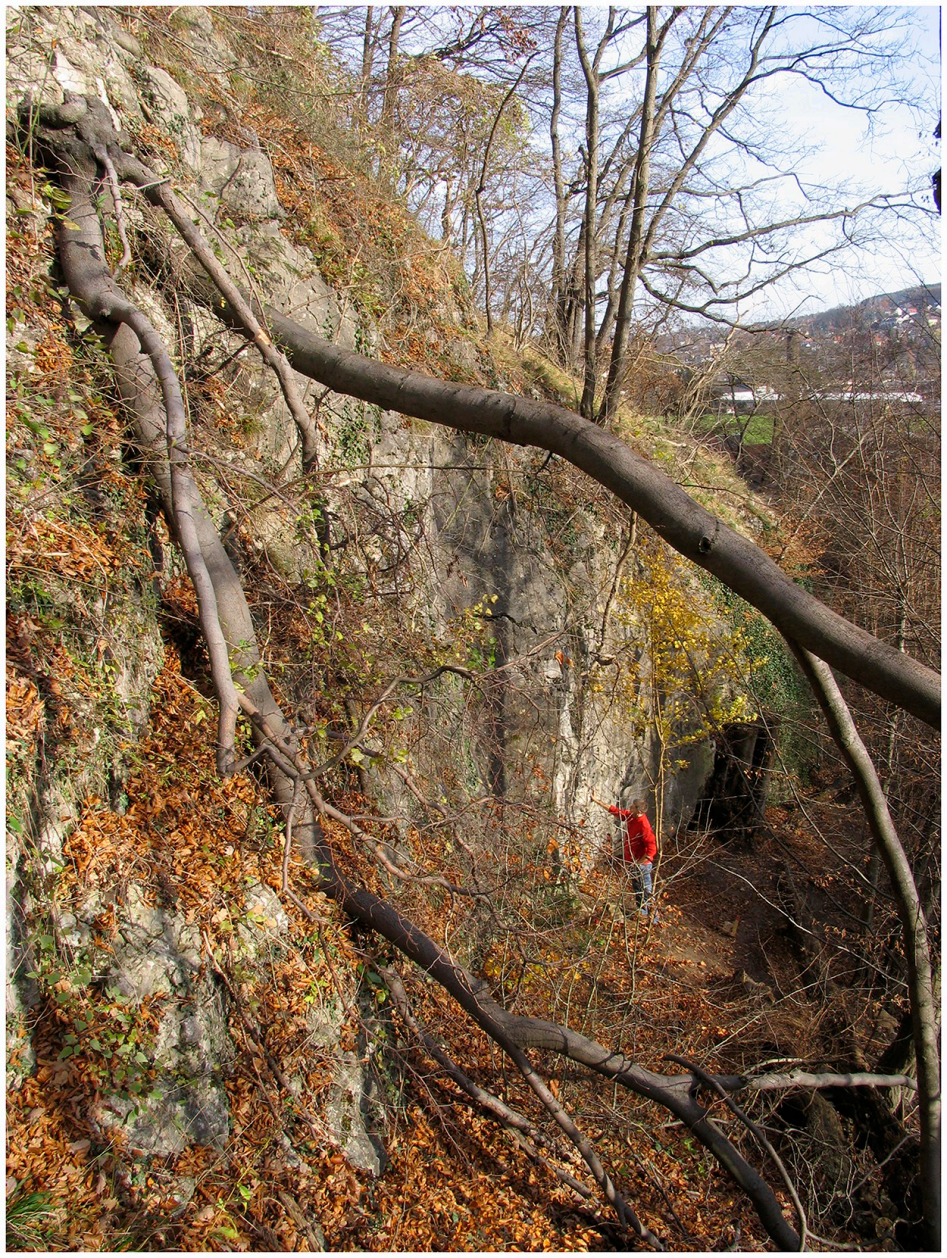
View from NW on the *Blätterhöhle* entrance area below a steep dolomite cliff of the *Weissenstein* with the *Lenne* valley in the background. –Photo: LWL-AfW Olpe/H. Menne.

**Fig 8:**
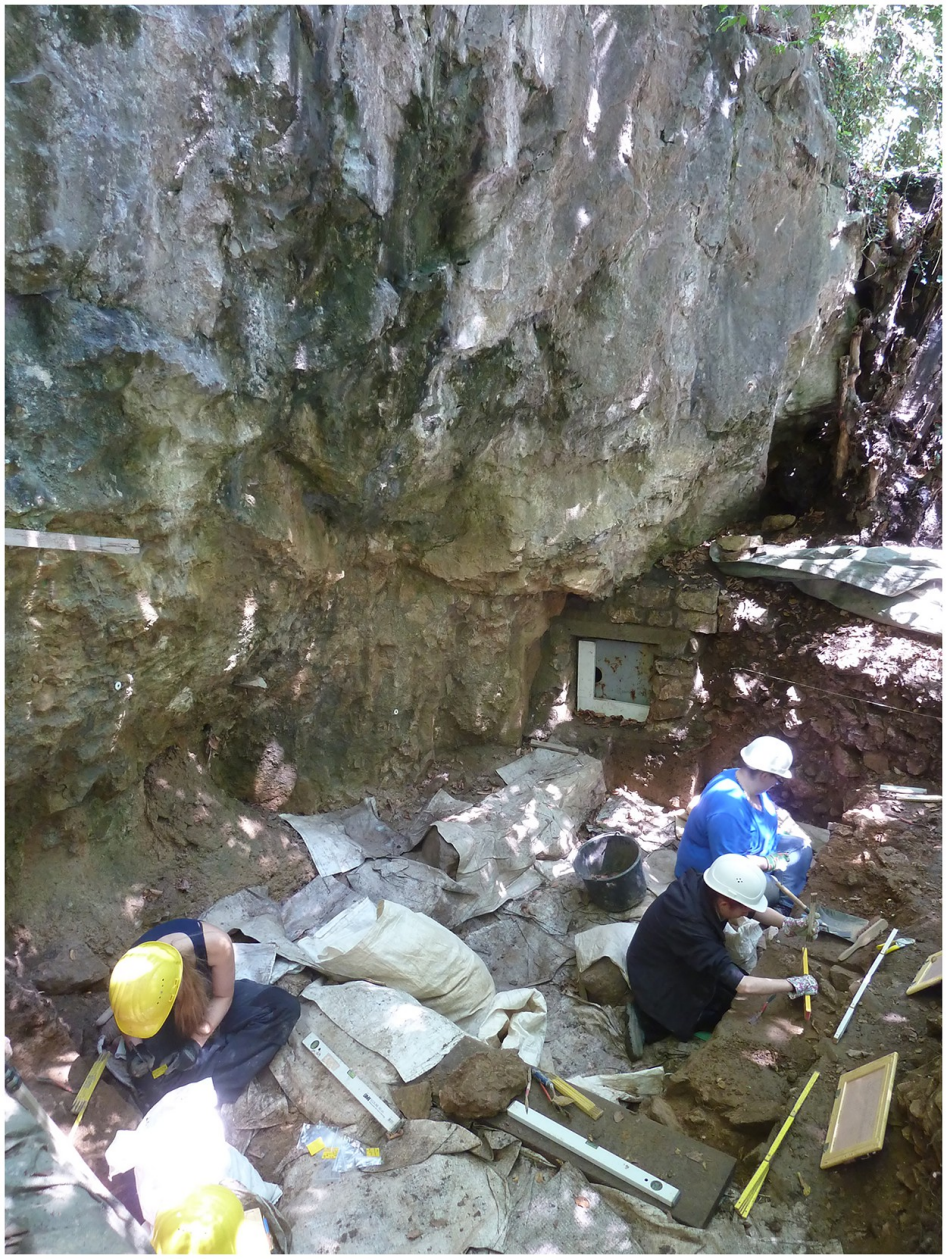
Excavation situation 2014 on the *Blätterhöhle* entrance area (*Vorplatz*) with the 2nd cave entrance either secured by a protective gate (in the background). –Photo: LWL-AfW Olpe/M. Baales.

To the east and south of the small cave entrance 1 (Fig 6), only a narrow strip of terrain was present below an approximately 10-metre-high dolomite cliff and above a steep slope (Fig 7). A further cave entrance 2, also secured by a gate (Fig 8), was later found a little lower and a few metres to the east, but this has not yet been examined in full detail.

**Fig 9:**
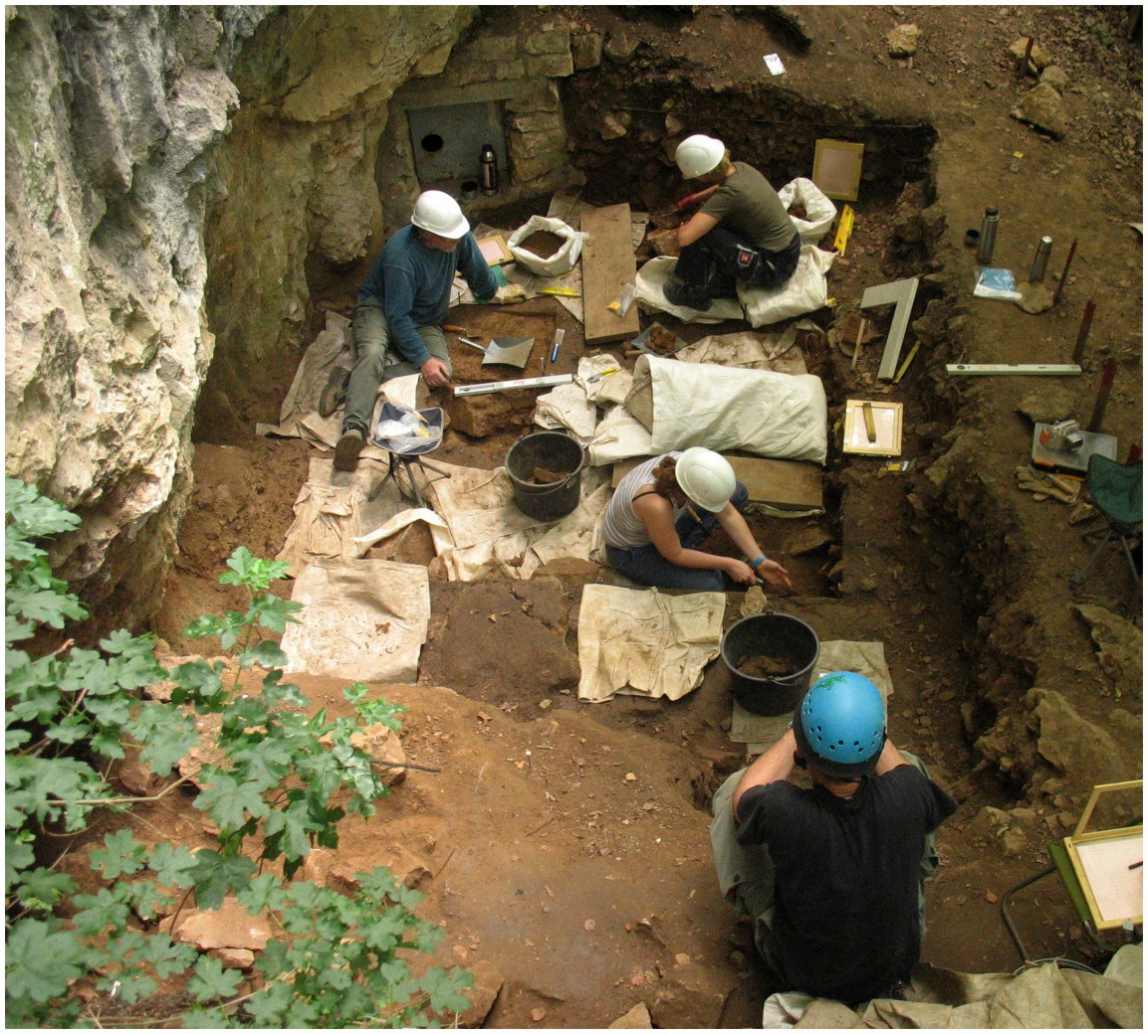
Excavation work 2009 on the *Blätterhöhle* entrance area in the Mesolithic find horizons. –Photo: City of Hagen/J. Orschiedt.

**Fig 10:**
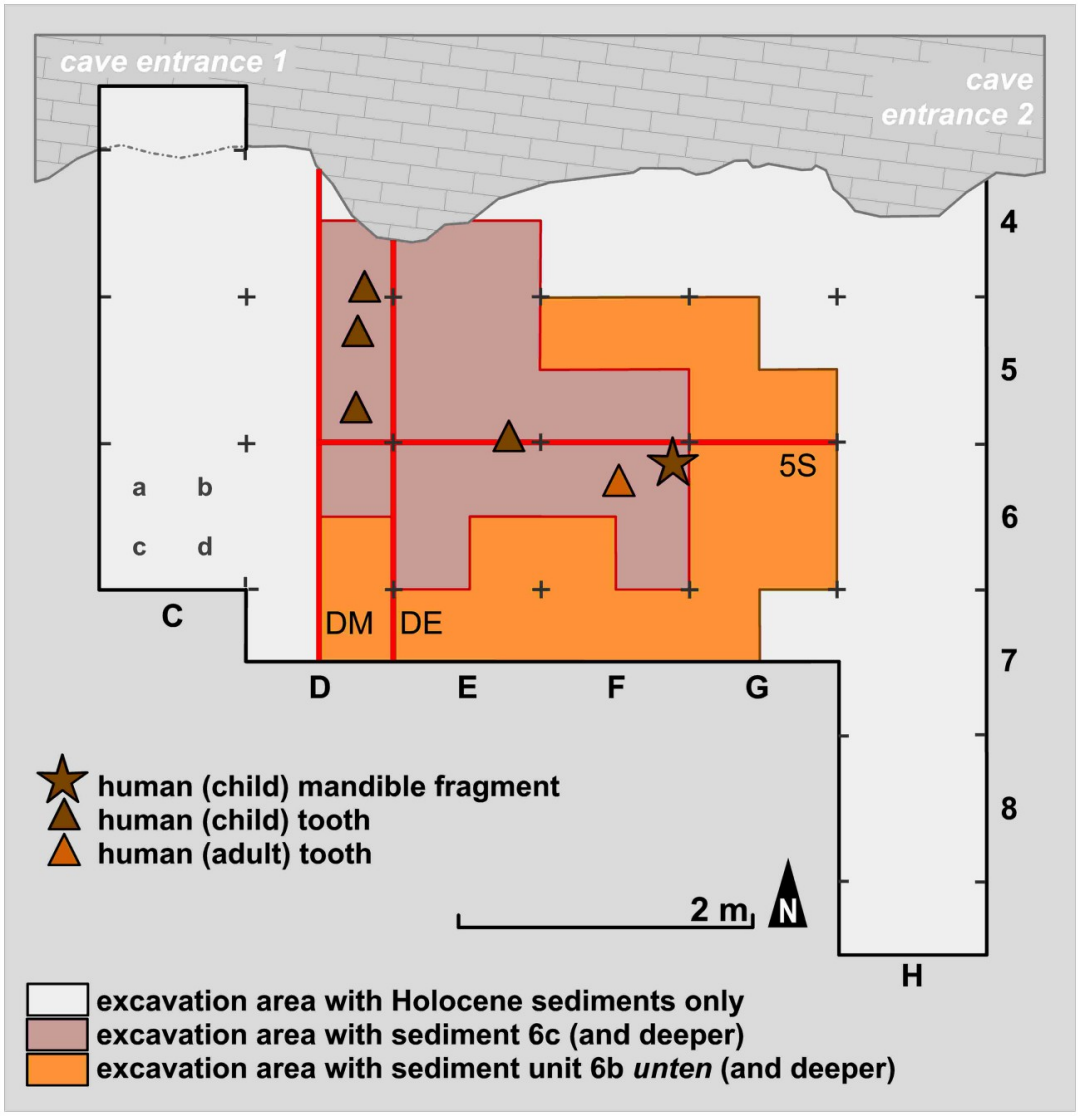
Map of the archaeologically investigated area in the *Blätterhöhle* entrance area. In the southernmost and easternmost excavation squares the grey sediment 6c was only partially present. The profiles DM, DE and 5S are indicated (see below), as well as the location of the Final Palaeolithic human remains.–Graphic: LWL-AfW Olpe/M. Baales.

In 2006 an attempt was made to clarify the archaeological potential of the *Vorplatz* (entrance area) through archaeological excavations (Fig 9). In the past 15 years, in addition to a sequence of Mesolithic find horizons with fireplace zones and further Early Mesolithic human remains that is unique for the wider region [59, 60, 73, 81–84], it has been possible to investigate on a relatively small area of some 9 m^2^ Final Pleistocene sediments (Fig 10) with unexpected archaeological finds.

Core drilling carried out by the Geographical Institute of the University of Cologne has revealed a further clastic sedimentary sequence of some 4 m below. Therefore, in principle, even older archaeological find horizons can be expected. Indications of this could be a piece of charcoal which was recovered from sediment 6c and subsequently ^14^C-dated (MAMS 43642: 48,500 ± 770 BP; QxCal: 48,051–47,273 cal BC 2-sigma; cf. Table 2) to the Upper Pleistocene, and a tooth of a cave hyena (N. Nolde) which was recovered from sediment unit 6b/8 in the eastern sector of the excavation area in 2019.

## Excavation of the entrance area to the *Blätterhöhle* cave with Pleistocene sediments

### Introduction

The following presentation of our research findings to date is based on the excavation results of the 2015–2021 campaigns, which were carried out by the Olpe department of the *LWL-Archäologie für Westfalen* (State authority of Monuments and archaeological heritage in Westphalia) in cooperation with the city of Hagen and the speleological working group *Arbeitskreis Kluterthöhle e*.*V*. (Ennepetal, South Westphalia). The legal basis of the field research is the Monument Protection Act (*Denkmalschutzgesetz*) of the federal state of North Rhine-Westphalia, which explicitly supports the exploration of the archaeological heritage, which represents thus an important task of the *LWL-Archäologie für Westfalen*. The *Blätterhöhle* forecourt is currently the only known site where–for the first time ever–both a Mesolithic stratigraphic sequence and the transition to the preceding Final Palaeolithic can be investigated for North Rhine-Westphalia and neighbouring regions. Our work and the results obtained therefore represent basic research for this period.

The entire excavation area of the entrance area of the *Blätterhöhle* is oriented along the west-east rock face that borders it in the north resulting in it being exposed to the south (Figs 5 and 10). The excavation area is approximately 10 m above street level and today seals off the former valley floor of the *Milchenbach* rivulet flowing from the west. It can be deduced that the morphology of the terrain would have been much more pronounced in the past.

The first excavations were only made possible after a large overlying block of debris had been dismantled (cf. Fig 21). This allowed for the safe of sediments deposited below and once formed a cantilevered rock roof until the younger Holocene (while the youngest Mesolithic find horizons lay underneath).

**Fig 11:**
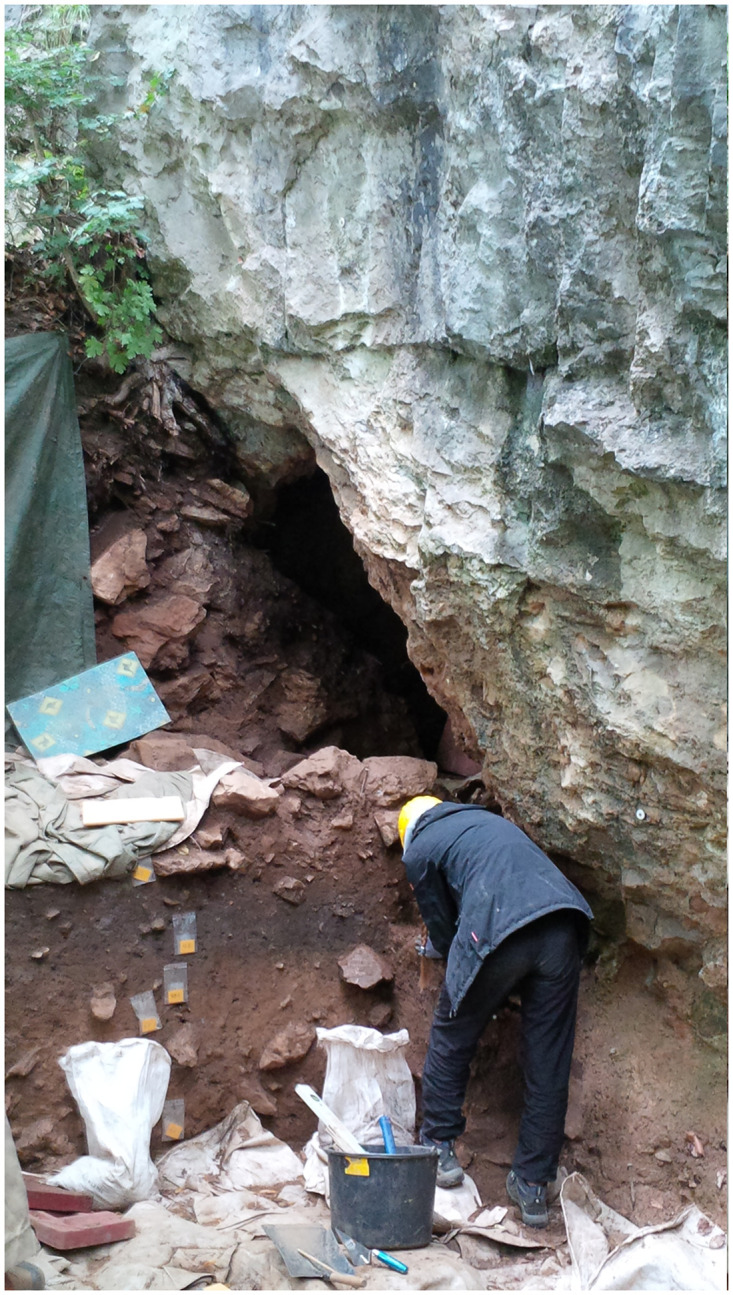
Cave entrance 1 above the NW corner of the 2014 excavation area. A Mesolithic combustion feature (dark colouring) can be seen in profile.–Photo: LWL-AfW Olpe/M. Baales.

In total, it was possible to examine nearly 22 m^2^ at different depths in front of the entrance area to the *Blätterhöhle* (Fig 11) while the core area of excavation containing Final Palaeolithic finds only covered an area of approximately 3 x 3 m (Fig 10). The more prominent sediment 6c could not be examined and documented over the entire investigation area, but only over an area of 6.5 m^2^. To the southeast of this area, sediment 6c was no longer preserved, and the characteristic grey colouration (see below) had disappeared to the south and east. Here, the stratigraphic equivalents were the medium-brown sediment unit 6b *unten* (basal part of sediment 6b, otherwise located above sediment 6c), and a mixed horizon 6b/8, with sediment 8 underlying the entire sequence (see below). Sediment 8, which was lighter in colour in comparison to the other sediments, was accessed throughout the entire excavation area with sediment 6c present; a further layer of sediment in the footwall (sediment 9) was accessible only in part. All these sediments or sediment units are presented and described in more detail below. No typical Mesolithic artefacts (especially geometric microliths) were found in these sediments and during the course of the excavation they had already been identified as originating from the (Final) Pleistocene.

The Final Pleistocene find sediment 6c was first recorded in 2016 in the area of the E5a excavation square [60, 84] before being examined further in all directions [85–89]. To the west and south, these surveys have been limited by the current profiles; further excavation was initially not possible here. The investigation of the area near the back wall of the rock in the north of the excavation area turned out to be very difficult as a result of the increasing sintering of the sediment and was even stopped in places or could only be continued after a winter break (frosty weather led to a partial disruption of the sinter).

The last excavation season exploring the Pleistocene sediments has taken place in 2021, as the ground conditions that had been created have not allowed further work in the basal sediments to take place [89]. Continued exploration into the adjacent area to the west will only be possible after the completion of the excavation of the hanging Holocene sediments which was begun in 2022.

### Profiles

**Fig 12:**
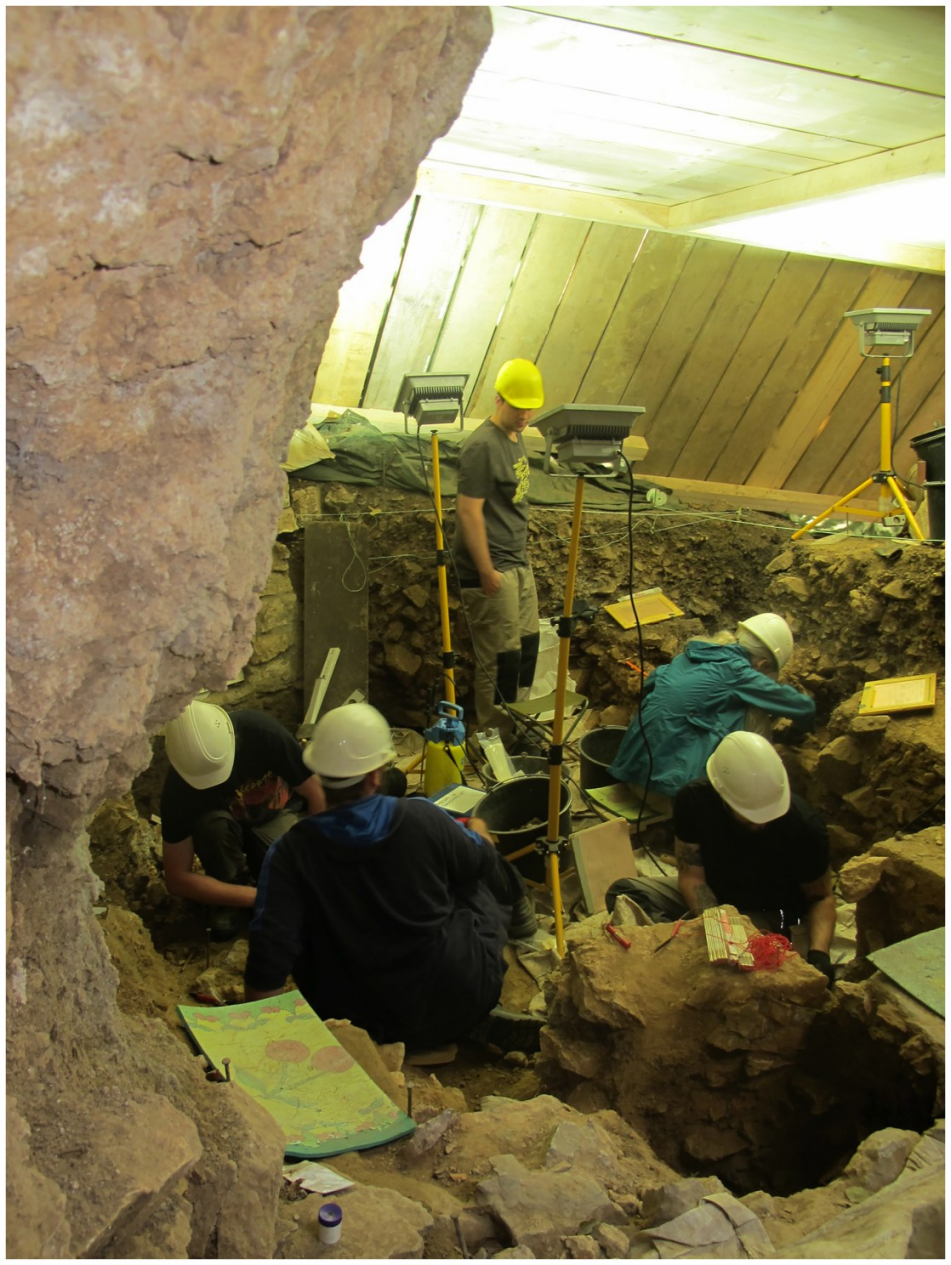
Excavation work under a protective roof in lower levels of the *Blätterhöhle* entrance area in 2016. –Photo: City of Hagen/W. Heuschen.

**Fig 13:**
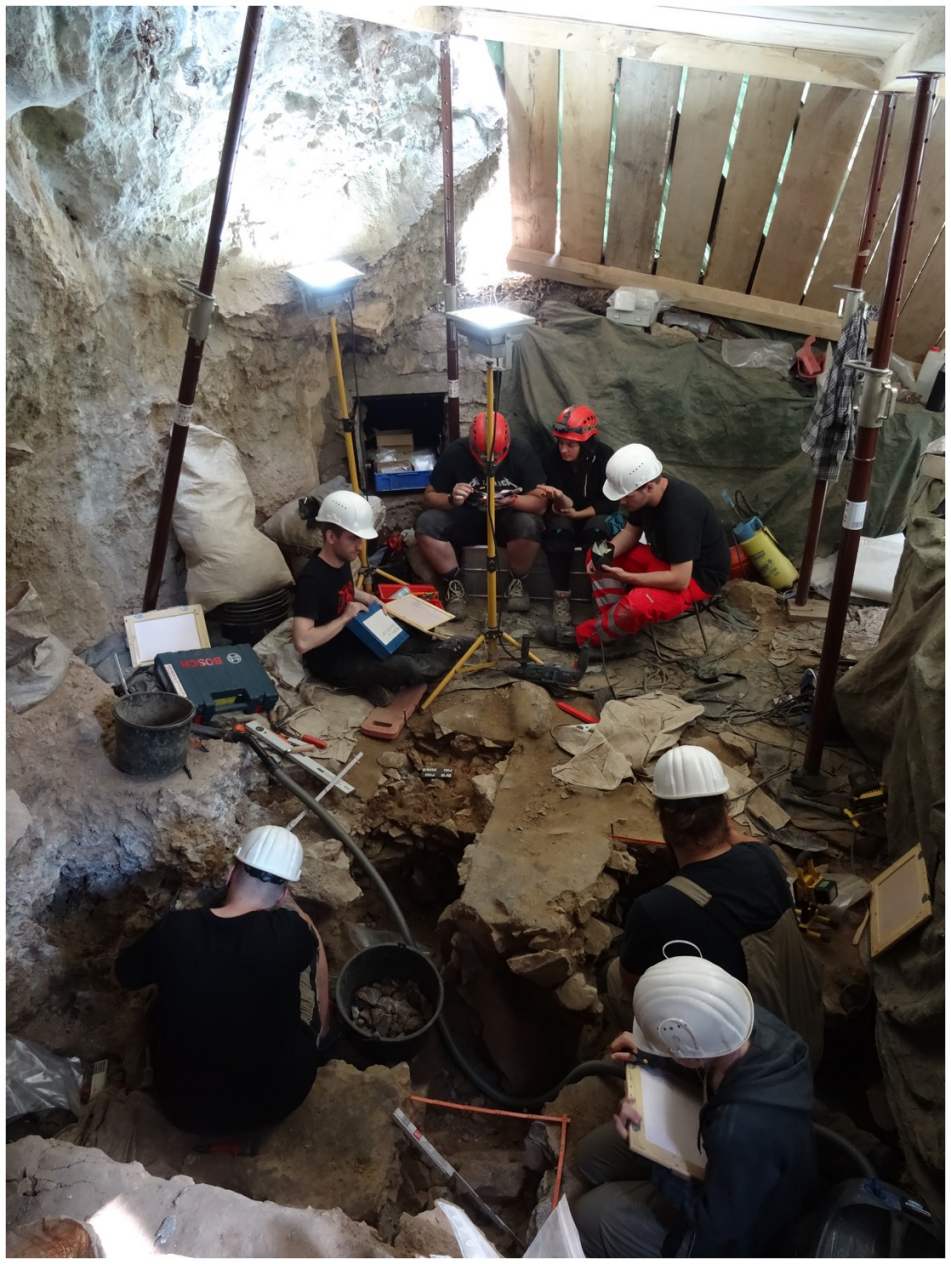
In 2019 the excavation work is further advanced. –Photo: LWL-AfW Olpe/M. Baales.

**Fig 14:**
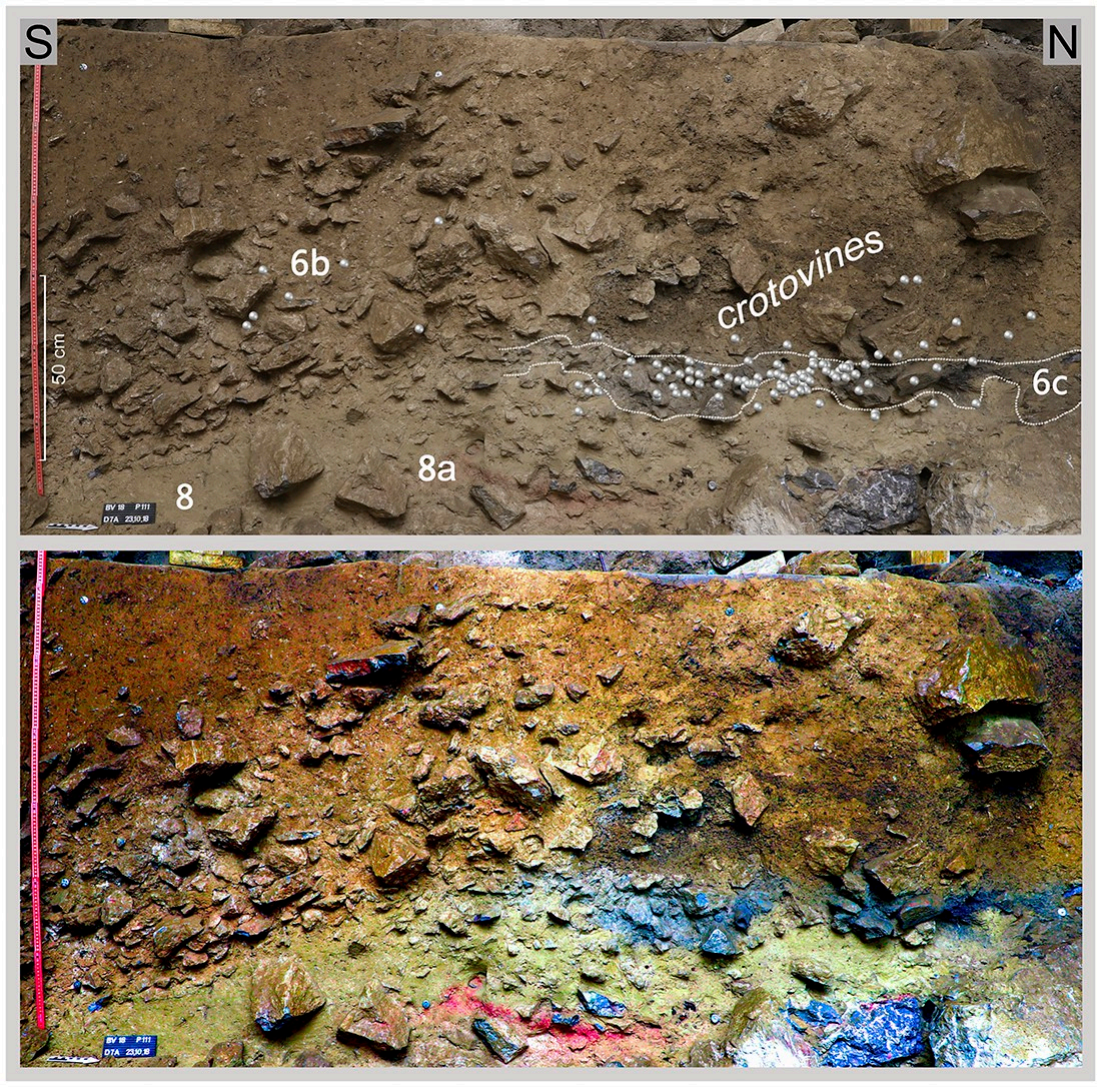
Profile DM. Top: Projected are the flint finds of sediment units 6b *unten*, 6b/8 and sediment 6c, from the directly adjacent excavation quarters; in addition, sediment 6c is highlighted. Below: False colour representation with DStretch. Sediment 6c and the red layer 8a stand out very clearly.–Photos/Data: City of Hagen/W. Heuschen, Ruhr-University Bochum/Annika Manz; compilation: LWL-AfW Olpe/D. Riemenschneider.

Among others, in the western part of the excavation area two larger continuous profiles were created (cf. Fig 10), documented and further investigated during the 2016–2021 excavations (cf. Figs 12 and 13). These have provided information about the composition and the stratigraphic position of sediment 6c, the sediments above and below. These two profiles, DE (max. height 195 cm; cf. Fig 21) and DM (max. height 160 cm; Fig 14), as well as other shorter sections in the eastern part of the excavation area, were the basis for numerous scientific investigations, such as micromorphological analyses, mollusc analyses, etc.; it should also be noted here that root tubes and animal burrows were sometimes still quite deeply preserved.

### Sediment sequence: General description

By and large it was possible to access a long sequence of sediments ranging from the recent humus-rich topsoil to the Pleistocene sediments within the entrance area of the *Blätterhöhle* which was partly covered by the aforementioned broken down rock roof of the former rock shelter (cf. Fig 21). Although a detailed and scientific evaluation is not yet available, a further more than 4-metre-long sediment sequence below the sediments was exposed by the aforementioned core drilling.

The conditions of sedimentation in the entrance area of the *Blätterhöhle* were extremely diverse even becoming apparent on a small scale:

- the sequence of sediments under the rock shelter close to the rock face is heavily sintered in parts (caused by the outflow of calcareous water). This was an area which would have been relatively sheltered and therefore preserved evidence of various human activities, for example the hearth zones
- under the eaves of the former shelter, slightly sintered sediment has been preserved in places, but it has been subject to the effects of the weather and also dripping water
- away from the shelter, the sediments were even more exposed to the weather conditions and as a result the sediments were not sintered here

These small-scale changes in sedimentation conditions are also reflected in the different characteristics and composition of the individual sediments defined.

In accordance with the shape of the recent slope, the Pleistocene (but also the overhanging Holocene) sediments dip to varying degrees both to the south and to the east. This phenomenon needs to be taken into account when preparing and evaluating profile projections of archaeological finds. The characteristics of the basal sediments are more closely detailed below; as already mentioned, these did not contain any typical Mesolithic artefact finds and are thus most likely to be Pleistocene relics. The sediments/sedimentary units 6b, 6c, 8/8a, and 9 taken from the hanging wall to the footwall are briefly characterised and include the archaeological finds contained therein:

#### Sediment 6b

Under Holocene layers that are in parts 2 metres thick, the medium-brown sediment 6b with a maximum thickness of approximately 95 cm was located (Fig 14). It is interspersed with limestone debris, some of which includes larger broken off boulders, especially in the lower part of the sediment. As well as recent root remains, crotovines have also been formed here and archaeological finds in the form of larger animal remains, charcoal, silex artefacts and river pebbles (manuports) with signs of wear were also found in this sediment. In the upper part of sediment 6b these relics can be assigned to the Early Mesolithic. Deeper in the sediment, about 30–40 cm above its base, from about 127.40 metres below ground level (BGL) on the S-N main profile DE, silex artefacts appear that can be dated back to the Final Palaeolithic. Macroscopically, this sedimentary unit 6b *unten* (6b base) is indistinguishable from the upper part but for the increase in the proportion of stone content within it. As the boundaries of the base area were often very blurred, the sedimentary units "6b/c" and "6b/8" were used to address transitional levels.

#### Sediment 6c

Below sediment 6b, but only in the area towards the rock face or cave entrance 1 and under the former rock roof (= large boulder in the overhanging sediment; Fig 10), a clear change in the colour of the sediment is evident. During the excavation, this distinctive grey to black-grey colour was identified (Fig 14) as being related to the charcoal and ash particles contained within the sediment, which was confirmed by further analyses. The grey colouring disappears macroscopically to the south and east (i.e. outside the area of the former large block above).

In sediment 6c, in addition to the larger limestone blocks, limestone pieces of up to about 10 cm in size were often observed and these appeared to be noticeably weathered or sintered. These limestones were also to be found to the south at the corresponding depth level outside the grey-coloured sediment. Sediment 6c dips to the east and south, consistent with the recent slope shape.

Only very few larger red clay aggregates and charcoal pieces of barely 3 mm in size could be found in the sediment. Although some degree of caution needs to be exercised here because of the relatively small area of the excavation, the increasing grey colouration of the sediment towards the north-west can be assumed as indicating the existence of a nearby combustion feature. This would be roughly in the same terrain position as the Mesolithic combustion features found higher up in the sequence.

Numerous Final Palaeolithic lithic artefacts, several mostly platy boulders with signs of wear, and some larger animal remains were recovered from sediment 6c which was in parts up to 15 cm thick.

#### Sediment 8/8a

Below sediment 6c and in places directly below sediment unit 6b *unten*, a yellowish, silty sediment (Fig 14) appeared which was also excavated to a certain degree. Where sediment 9 (see below) was accessed in the footwall, sediment 8 had a thickness of 50–70 cm and very large boulders were to be found in this sediment.

In the north-west corner of the excavation area, partly sintered block rubble was found at the base of sediment 8, the interstices of which were almost free of sediment and thus cavities had formed. Future excavations are needed to verify whether this phenomenon is associated with a significantly larger cave entrance 1 or with another previously undiscovered deeper cave entrance 3.

Approximately in the middle of the western edge of the excavation area a reddish east-dipping horizon up to 2 cm thick (labelled “sediment 8a”; Fig 14) was found within sediment 8. The reddish colouring found here is strongly indicative of the presence of hematite in the ground. However, such sediment sections have occasionally occurred in a very similar form during the weathering of sinter and under large limestone strata. The results of investigations carried out by restorers from the Archaeological Heritage Service in Münster using a portable XRF device proved a high content of iron (Fe); this may presumably be attributed to geological processes.

Sediment 8 is remarkably poor in finds with only a few silices having been discovered at the boundary between sediments 6c and 8 and in the southeast of the area. Most of the artefacts found here come from the water screening; charcoal and bone fragments are completely absent.

#### Sediment 9

A somewhat different colouration was to be seen at the base of sediment 8 in the southwestern excavation area. Sediment 9 was light-brown / grey in colour and differing only slightly from sediment 8 but completely devoid of any finds. The lower limit of sediment 9 could not be reached as the exposed thickness was 40 cm. Future excavations are needed to confirm whether or not this is–as presumed here–a local phenomenon present in the area of the former eaves of the rock shelter or ultimately an extensive further sediment layer.

### Micromorphological analysis of the sediments/sediment units 6a to 8

#### Material and method

In addition to the observations made in the field during the excavation, micromorphological analyses were carried out to gain additional information about sediment composition and processes of sediment accumulation and post-sedimentary overprinting by turbations and relocation processes or by soil formation. The micromorphological analysis of thin sections of sediment is very well suited for this purpose because sediment components can be identified and their microscale spatial arrangement can be examined [90, 91]. In addition to the indication of sedimentary and post-sedimentary processes, there are often indications of ground frost [92, 93], which in turn can help in the chronological classification of individual sediment packages.

In the course of the excavations on the entrance area of the *Blätterhöhle* sediment blocks were removed to create thin sections in order to enable a micromorphological characterisation of layer 6c and the stratigraphically neighbouring layers 8 and 6a/b.

The impregnation and preparation of the uncovered sections was carried out by the Thomas Beckmann company, Schwülper-Lagesbüttel, using the method described in [94]. In total, 14 thin sections were made available for the analysis (cf. S1 File).

Further information on the sampling strategy, the applied micromorphological methods and a detailed description of the thin sections is provided in the S1 File and the S1 Table.

#### Results

Sediment 8 is dominated by grains of silt, shows a coherent structure and is rich in grains of detrital calcite (S2 Fig). These features are typical of loess, which was probably deposited here at the end of the last glacial period. In places, both sharp-edged to weakly rounded limestone fragments are present, which come from the rock roof. After the sediment had been deposited, only a slight overprinting by pedogenetic processes took place. Slightly distinct silt cappings indicate the impact of ground frost [92, 93] which suggests an overprint of the sediment during the Late Glacial or Younger Dryas. Passage features as well as biogenic pores and aggregates show weak overprinting by soil animals [95]. The fine biopores (channels) that are quite common here were created by fine plant roots, which, however, also stem from recent vegetation, as can be deduced from some fresh root remains. In areas adjacent to this, differently coloured areas of sediment were formed by the limited incorporation of grey-coloured sediment from sediment 6c.

There is no evidence of carbonate dissolution or other processes of initial soil formation, yet there are many hypocoatings of micritic calcite formed by percolation of bicarbonate-rich leachate and its intrusion into the matrix with secondary calcite deposition [96]. This, in turn, is indicative of a partial decalcification in the layers above sediment 8. Sediment 8 was probably deposited before the Late Glacial Interstadials under dry-cold climatic conditions when hardly any soil formation occurred.

Sediment 6c has a darker colour, which is mainly caused by finely divided charcoal fragments and humus particles. There are also a few fragments of bone, which also occur in sections from the hanging sediments 6a/b. High amounts of primary calcite grains in the silt fraction and a crystallitic b-fabric indicate that layer 6c contains a great deal of loess and has not been altered by carbonate dissolution. Locally occurring silt cappings indicate frost that may have overprinted the sediment during the Younger Dryas. Apart from the interference of anthropogenic components (charcoal, bones) and the darker colouration (S1 Fig), sediment 6c strongly resembles the loess of sediment 8. Frequent secondary carbonate precipitations indicate stronger carbonate dynamics, whereby the carbonate dissolution has presumably taken place in the overlying sediments, because the sediment 6c itself hardly shows any decalcification zones. The carbonate enrichment could have occurred either during the Late Glacial Interstadial or during the Holocene pedogenesis. Although carbonate dissolution is unlikely to have played a role in the formation of sediment 6c, no ash particles considered to be readily soluble have been detected. Furthermore, there are no indications of sediment discolouration due to the influence of heat or significant concentrations of charcoal particles. This indicates that the thin sections of sediment 6c examined here do not originate from an *in-situ* fireplace, but refer to the adjacent area.

Sediments 6a and b are calcareous, silt-dominated hillside sediments. Complete decalcification and the beginning of clay mobilisation can be seen in thin sections taken from squares D6b and G7a, which is reflected in the absence of fine detrital carbonate particles and in the development of a stipple-speckled b-fabric and thin clay coatings. These entrance areas are presumed to have been fully covered by Holocene soil formation, whilst the samples from square D 5b, closer to the rock face, show little carbonate dissolution and are assumed to have been protected by the rock roof.

#### Conclusion

The micromorphological investigations documented here provide a detailed insight into the material composition and spatial organisation of the sediments 6a to 6c and 8 of the entrance area to the *Blätterhöhle*. The processes of sediment formation and pedogenesis derived from this can thus be reconstructed in terms of chronological sequence. Aeolian accumulation of mineral dust, the accumulation and partial dissolution of limestone fragments, bioturbation and fabric formation, dissolution of fine detrital calcite and secondary calcification can be considered to be natural processes, whilst on the other hand charcoal and bone accumulation represent anthropogenic inputs. The intensity of carbonate dissolution in particular shows spatial differences that are probably assignable to the protective effect of the former rock roof. Sediment 6c is similar in grain size and mineral composition to that of layer 8, which can be described as loess, but is significantly enriched with charcoal and bone.

### Pollen and vegetal macroremains

Investigations carried out by the Institute for Pre- and Protohistory at the University of Cologne in 2008 and 2017 showed that in the sediments of the cave entrance area–in contrast to those in the cave–no pollen nor phytoliths have been preserved. On the other hand, however, charcoal exists both in the cave and in the entrance area. While pieces of charcoal in the cave are well preserved and some of them measured several centimetres in size, they are considerably smaller in the entrance area of the *Blätterhöhle*. The charcoal finds there are mostly in the form of charcoal spangles, with larger chunks of up to 3 mm in size being the exception. Especially in the area of sediment 6c, where its grey colouring is a result of charcoal particles, only a few datable (see below), larger charcoals could be recovered in the course of the excavation, but could not be determined to species.

### Larger faunal remains from the sediments/sediment units 6b *unten*, 6b/8, 6c and 8

#### Material and method

**Fig 15:**
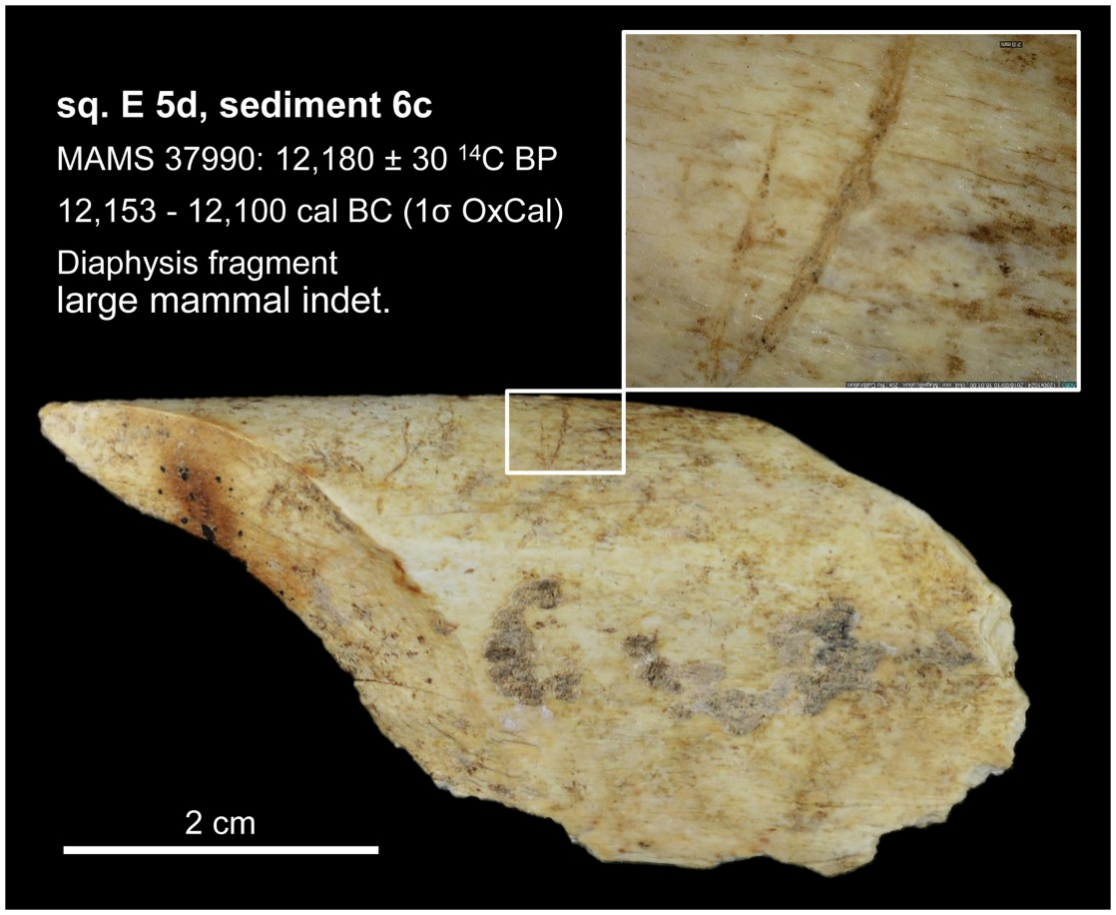
Small fragment of a larger mammal diaphysis cracked open while fresh with cut marks. The fragment comes from sediment 6c and was radiocarbon dated to about 12.1 kyr cal BC.–Photos: J. Orschiedt; compilation: LWL-AfW Olpe/M. Baales.

From the sediment units 6b *unten*, 6b/c, and 6b/8 as well as sediments 6c and 8, a total of 296 larger faunal remains have been analysed so far (the numerous small mammal and further faunal remains found in the sediments have not yet been studied). These have mostly been in the form of very small fragments–sometimes with signs of fresh bone breaking and/or cutmarks (Fig 15), but this has not been studied in detail yet–which only in rare cases have made it possible to determine the species precisely by comparison using the reference collection of the Archaeozoological Laboratory of the Cologne University. In mammal bones, the thickness of the bone cortex could often be used to infer the size of the respective animal species. Only around 4% (n = 12) of the bone finds could be identified wih a high degree of precision and these are divided into the following six species: red deer (*Cervus elaphus*), wild cat (*Felis silvestris*), red fox (*Vulpes vulpes*) a large canid (wolf, *Canis lupus* or domestic dog, *Canis familiaris*), wild boar (*Sus scrofa*) and European hamster (*Cricetus cricetus*). These finds are discussed in more detail below.

#### Results

Four fragments found in the said sediments represent the remains of the red deer. They comprise a zygomatic fragment, an upper jaw molar, the joint part of a mandible and a dorsal fragment from a metacarpal bone (MNI = 1). Red deer are among the largest deer species in Europe and are now widespread, albeit patchily, in the temperate zones of the Holarctic [97]. According to Wilfried Bützler [97], their current occurrence, which is mostly limited to forests, is a result of human settlement, which pushed the red deer into less favourable areas. The preferred biotopes normally also include sparse forests, alluvial forests, open grassland and heathland, but mountainous spruce forests are also populated [97]. Red deer are among the most climate-tolerant species that have been part of the European large mammal spectrum since the Middle Pleistocene [98], but retreated to more temperate refuges during the Lateglacial Maximum [99]. Dated remains of deer show that the red deer migrated northwards from their southern glacial retreat areas as early as the beginning of the Lateglacial warming phase (GI-1e, Meiendorf-Interstadial [34, 99–103]). Areas where the average January temperature was above -10°C [104] were preferred as areas for resettlement. Records of red deer from the Younger Dryas are rare and mostly limited to more southern regions, but deer remains from Great Britain and Belgium dated to this period [99] show that a more northerly distribution, such as the region of the *Blätterhöhle*, with recurrent immigration and emigration during the warmer and colder phases at the end of the last ice age could well have been possible.

Five other bone fragments can be assigned to the Cervidae (deer) family which includes reindeer (*Rangifer tarandus*). During the Younger Dryas in the North German Plain and the adjacent uplands to the south, these were among the main hunted prey [9, 49, 53, 105] and evidence of this was anticipated for the period under investigation at the *Blätterhöhle*, but no reliable evidence has yet been found.

The discovery of the humerus of a wild cat, a maxillary canine of a red fox, and a maxillary second molar and proximal fragment of a second metacarpal of a large canid, all provide evidence of the existence of predators.

Today, the wild cat is mostly found in larger, contiguous forest areas in Europe, Africa, western, central and southern Asia [106]. According to Helmut Hemmer [106], the height of the snow cover is a limiting factor influencing their current distribution. It is difficult for the animals to move easily if there is more than 20 cm of loose snow. Recent observations of wild cats in the Swiss Jura have shown that wild cats migrate to snow-free areas in winter and spring, only to return to their customary territories after the snow has melted [107]. During the cold periods, the wild cat was therefore only to be found in warmer climate zones [108]. From these glacial refuges, with the onset of warming and reforestation during the Allerød, they also reached areas north of the Alps for the first time [cf. 109]. Although their preferred habitat is often associated with dense forest cover in more temperate latitudes [cf. 106, 110], their current habitat preference covers a much broader spectrum, including open to treeless scrub and grassland [111]. An example of the wildcat’s adaptability is evident from habitat studies conducted in Scotland [112]. They prove that the preferred habitats of wild cats are not those of dense forests, but rather of open, treeless areas [cf. 111, 113]. In general, the availability of forms of shelter, the presence of prey [112, 114] and the maximum snow depth [106] are more important for habitat selection than dense tree populations. In light of this, the distribution of the wild cat at the end of the last ice age and thus its reputation as a "forest indicator" needs to be reconsidered. In view of the discovery of the cat humerus in Sediment 8, which can be classified as a glacial loess formation (see above), the possibility of mixing of younger strata by burrowing animals (e.g. badgers, foxes, hamsters) has to be considered. With this in mind, the cat bone remains a controversial find.

The distribution of the red fox now basically covers the entire European continent, large parts of Asia, North America and North Africa up to altitudes of approximately 3000 m [115]. Similar to that of the wild cat, the habitat of the red fox is determined more by the food supply, which consists of rodents, mainly field mice, rabbits, hares, insects and vegetable food, and by the shelter available [115]. The distribution of the red fox overlaps with that of the somewhat smaller arctic fox (*Vulpes* or *Alopex lagopus*) in the arctic tundra regions. In the overlapping areas, both species are in fierce competition, but the odds are more in favour of the red fox in the course of the progressive loss of habitat and the advance of red fox far into the tundra in recent decades [116, 117]. A distinction between subfossil bones is often made possible because of the difference in size between the two species. During the Last Glacial Maximum, there are numerous records of the red fox in their classic retreat areas, but also in the Carpathians and the Dordogne, while in Central Europe and in regions further north they disappear completely [118]. Only the polar fox is represented there during this time. Only at the end of the Last Glacial Maximum did the red fox migrate north again [cf. 119], but it cannot be completely ruled out that the bones examined here belong to the arctic fox. Both species reappear together in French and German sites during GI-1 [118]. Numerous well-dated records of the red fox for the Younger Dryas in northern and southern Germany [49, 120] highlight their adaptability even during glacial phases [cf. 118]. The red fox is documented in sediment 6c of the *Blätterhöhle* site by an upper canine, but again an intermingling of somewhat younger objects into this sediment cannot be completely ruled out here either (see above).

The wolf was widespread during the Pleistocene and Holocene throughout the Palearctic and Nearctic regions from North America to southern India and in all habitats except high alpine areas. By the middle of the 18th century, the persecution and hunting that had persisted since the Middle Ages led to targeted extermination in Germany [121]. Wolves have been regularly found in various sites from the Pleniglacial and Lateglacial, although they were less dependent on glacial refugia than other mammals [119]. The two remains of a large canid retrieved from the rock shelter area of the *Blätterhöhle* come from Sediment 6c. The length of the maxillary tooth, measuring 8.4 mm, falls within the variation range of large dogs (e.g. the German shepherd) as well as that of recent wolves [122]. A reliable assignment of these remains to dogs or wolves is therefore not possible with either of the two pieces.

As for the broken rib fragment of the badger (*Meles meles*) from sediment 6c, it can be assumed that it was secondarily displaced by burrowing activity, since the badger is a typical representative of the temperate, Holocene fauna of Central Europe. Moreover, some 40 individuals of badger have been documented as present in the overhanging layers and especially in those of the *Blätterhöhle* itself.

The second phalanx of a wild boar from sediment unit 6b/8 shows that the proximal articular surface is not fused and is that of a young animal less than 12 months old. In Europe, wild boar prefers larger deciduous and mixed forests, but they are also to be found in sparse forests in the Mediterranean region [123]. During the Last Glacial Maximum, wild boar was exclusively present in the southern European refugia like the Dordogne and the Balkans [102, 108]. However, there are records of their presence in more northern regions already during GI-1 [119, 124–126]. It can be assumed that the Younger Dryas Period cooling led to a renewed disappearance from northern Central Europe as there is no reliable evidence from corresponding find strata. In this case too, it is likely that this find is the result of a secondary displacement.

The situation is different for the last species detected. An incomplete femur of a young European hamster was recovered from sediment 6c. This species generally prefers open biotopes in the temperate Palaearctic with deep loamy-clayey soils, especially with loess content [127] in which it can dig complex burrows [128]. The northern limit of distribution today is around 55° north latitude, which in Russia can extend to 59° latitude [128]. As Steppe dwellers, hamsters were originally bound to continental climate and habitat parameters; areas with high precipitation rates, sandy or rocky soils and dense forests were avoided [127]. The species is clearly documented for the late glacial period of southern Germany [129, 130] and the Allerød of the Neuwied Basin (Central Rhineland [131, 132]). In the northern edge of the low mountain range–analogous to the *Blätterhöhle*–there is also evidence of the Younger Dryas with the *Kartstein* in the North Eifel uplands [133]. The presence of European hamster in these climatically different periods with different vegetation underlines that the European hamster as a "Pleistocene relic" [134] has a certain ability to adapt to its environment and its presence at the *Blätterhöhle* therefore comes as no surprise.

### Molluscan fauna from sediment 6c

#### Material and method

What follows is a presentation of the mollusc content of three analysis units from a sediment column (excavation square D 6b) and another sediment sample from excavation square D 5a –all from sediment unit 6c. The task was to obtain first informations (a full investigation of the mollusc remains is in progress) about the sedimentation environment, biostratigraphic classification and the landscape at that time by means of sieve analyses and the selection and determination of the remains of molluscs through the use of light microscopy.

For the purpose of interpreting the following results it should be noted that this is not an analysis of the excavation of a block of original sediment, but of the archaeologically extracted material removed. It is equally important to emphasise the fact that the sample volumes that were evaluated sometimes differ significantly from one another as a result of the strong penetration of the profile with larger rock falls. While with a homogeneous sediment structure each analysis unit (with an area of 50 x 50 cm and a layer height of 5 cm) would have had a volume of 12.5 litres, here (when large stones occur) there is a sometimes considerable reduction in the fossil-bearing material, which sometimes only comprises approximately 1 litre per analysis unit. Very few molluscs were recovered directly during the excavation; most mollusc remains were separated after water screening the sediments.

As a result of this sampling method, there is no directly comparable statistical validation for the horizon-related frequency of the identified taxa, which is why, for example, the absence of a species that regularly occurs in the hanging wall or footwall should not be taken as an indication of changed environmental conditions. Furthermore, the reliable determination of the shell residues in the sieve residues, which are generally only sparse and often only found in the form of fragments, is made even more difficult in samples with a small volume.

Finally, bioturbation and consequent displacement as well as (especially in the case of molluscs) active intrusion into ducts and cavities in the clastic sediment 6c must also be taken into account. The strong root penetration of the sediment analysed here with regard to the remains of roots in the analysis units, calls for appropriate caution.

Despite the limitations mentioned above, sufficient data was obtained to allow for a basic palaeoecological characterisation of sediment 6c in the case of the majority of the analysed units. However, the main focus here is on the overriding trend.

#### Results

Unfortunately, the samples examined here are characterised by a relatively low density of molluscs. A complete review was conducted but nonetheless only 62 identifiable conchylia could be separated and assigned to 17 different taxa (Table 1). As a result of the predominantly poor state of preservation and only a few intact individuals being found, the processing was often difficult. Some determinations are subsequently subject to degrees of uncertainty. This particularly relevant in the case of numerous shells of the genus *Pupilla* (pupa snails) where often only mouth fragments had been preserved, which did not allow all important features (neck bulge, dentition, curvature of the whorls, growth bands) to be clearly recognised.

**Table 1:** Identified species of molluscs from sediment 6c.

Species / Analysis Unit & depth	D 5a 127,29–127,19	D 6b 127,15–127,10	D 6b 127,10–127,05	D 6b 127,05–127,00
***Arianta arbustorum* /*Bradybaena fruticum*** *		1		
***Carychium tridentatum***	1			
***Cochlicopa* sp**.			1	
***Discus ruderatus***			1	
***Euconulus fulvus***		1		
***Nesovitrea hammonis***			1	
***Punctum pygmaeum***		2		
***Pupilla muscorum***		2		
***Pupilla* cf. *loessica***			11	3
***Pupilla* cf. *pratensis***	1	1	1	1
***Succinella oblonga***			1	
***Trochulus* cf. *hispidus***		3	4	
***Vallonia costata***		2	5	1
***Vallonia* sp**.			3	
***Vertigo pusilla***		2		
***Limacidae indet***.	3	3	3	3
***Pisidum* sp**.		1		

* the shell fragment shows the sculpting characteristic of both species, but is too small to be assigned to either species with any degree of certainty.

Owing to the small number of individuals found, no statistically reliable statements can be made, e.g. about the local forest-field distribution. While *Vertigo pusilla* and *Discus ruderatus* were considered indicators for forest (the latter especially for coniferous forest), *Vallonia costata* (Ribbed grass snail), *Pupilla muscorum* (Moss chrysalis snail) and *Pupilla loessica* (loess snail) were considered to be indicators of an open landscape. The simultaneous detection of *P*. *loessica*, which can also be considered to be an indicator of cold-period climatic conditions, and *Discus ruderatus*, which in turn is typical of early Holocene mollusc spectra, indicates a probable Lateglacial time position for the sedimentary unit 6c (possibly Younger Dryas period in the transition to the following Preboreal). However, in order to verify this classification, further detailed comparisons with the spectra from the overlying and hanging sediment layers have been deemed necessary and these are currently in progress.

In addition to the terrestrial mollusc remains, fine gravel, fish remains, ostracods and shell fragments of pea clams were also identified in the analysis units. This indicates that either a sporadic fluvial influence (phased flooding) is evident here, or the entry of corresponding allochthonous (sediment) material is due to human activities. With the exception of the sensitive ostracod and mussel shells, an entry based on wool from birds of prey that used resting and/or nesting opportunities on the rock face above the entrance area in front of the *Blätterhöhle* would also be conceivable.

### Geochronometric dating of the basal sediments

#### Luminescence dating

In the course of the examination of sediment 6c in 2018, an attempt was made to narrow down its deposition age using luminescence dating. One sample (No. 2) from sediment unit 6b *unten* in the hanging wall and two samples (1a and 1b, the last gave no reliable result) below sediment 6c (sediment 8) were recovered. No sample could be recovered from sediment 6c itself due to the large number of stones (for more informations on the sample and measurments methods cf. S1 File).

Sample 1a: square D6a (profile DM); item 187 (x 50; y 83; z 126.96); sediment 8

Sample 2: square E7c (WE-profile, southern limit of excavation area); item 134; x 18; y 50; z 127.28; sediment unit 6b *unten*

Susanne Lindauer (Curt-Engelhorn-Centre of Archaeometry, Mannheim) submitted the following age estimates and comments in respect of two sediment samples (letter S. Lindauer 30.07.2019):

Sample 1a, sediment 8 (MAL10383): 13,700 ± 950 kyr BP for fine-grain quartz and 8,000 ± 600 a BP (minimum age model) or 9,500 ± 500 kyr BP (central age model) for coarse-grain quartz.

Sample 2, sediment unit 6b *unten* (MAL10384): 11,200 ± 800 kyr BP for fine-grain quartz and 14,300 ± 900 kyr BP for fine-grain feldspar (feldspar bleaches more slowly than quartz and therefore often deviates)

The age estimates for the coarse and fine grain samples differ significantly. In the case of sample 1a they differ by around 4,000–5,000 years; there are also clear differences between quartz and feldspar. "This is an indication of different deposition conditions and/or ages of each grain size" (letter from S. Lindauer 30.07.2019).

Compared to the micromorphological analyses (see above), which allow the assumption of (at least) a GS-2 chronological deposition for sediments 8 and 6c, the average values of the measured ages are evidently far too young. Taking into account a double standard deviation for the sample fraction "fine grain quartz", the age of sediment 8 would be around 15,600 years and therefore within the expected range. The same fraction of sample 2 gave the expected age range (Early Holocene / late Final Pleistocene) for sediment unit 6b *unten* taking the simple standard deviation into account.

#### AMS-radiocarbon dating of bones and charcoal

The overhanging Holocene strata, Mesolithic fireplace zones and latent Mesolithic horizons have been ^14^C-dated multiple times using individual charcoal samples and the results fit well into the expected Preboreal—Boreal—Early Atlantic timeframe. This also applies to some age measurements performed on animal and human bones (especially those from the cave).

In the case of the basal sediment areas 6b *unten* and 6c, initially only bone samples were used for the ^14^C-dating and sometimes reveal an anthropogenic influence (cut marks, impact/fresh diaphyseal fragments). The samples were also dated in the Curt-Engelhorn-Centre of Archaeometry, Mannheim (Table 2), in accordance with the MAMS preparation protocol (including ultrafiltration; for a detailed description of the pre-treatment methodology cf. S1 File).

**Table 2:** AMS-^14^C-dates on bones and charcoal from the sediment unit 6b *unten* and sediment 6c (MAMS—Mannheim; KIA—Kiel). Calibration by using OxCal v4.4.4 [135].

Lab. ID	Sediment	Square	Material	δ^13^C ‰	^14^C BP	cal BC 1σ (OxCal)	cal BC 2σ (OxCal)
**MAMS 36031**	6b *unten*	E6d	bone	-16,5	10,760 ± 40	10,800–10,773	10,812–10,752
**MAMS 36032**	6b *unten*	E6c	bone	-12,1	11,793 ± 43	11,800–11,733	11,818–11,632
**KIA 55937** *	6b *unten*	E6c	bone	-17,1 ± 0,3	10,710 ± 40	11,611–11,555	11,668–11,521
**MAMS 36037**	6b *unten*	E6c	bone	-20,9	11,674 ± 46	11,641–11,594	11,659–11,489
**MAMS 37989**	6b *unten*	E7a	bone	-31,4	10,344 ± 25	10,158–10,091	10,248–10,045
**MAMS 43643**	6b *unten*	D5b	charcoal	-25,7	9,368 ± 29	8647–8609	8736–8556
**MAMS 43644**	6b *unten*	D7b	charcoal	-26,8	9,440 ± 29	8761–8701	8801–8629
**KIA 55613**	6c	D5d	charcoal	-26,0 ± 0,1	12,385 ± 50	12,594–12,338	12,898–12,239
**KIA 55615**	6c	E4d	charcoal	-20,3 ± 1,3	12,170 ± 80	12,247–12,056	12,378–11,852
**KIA 55616**	6c	F5c	charcoal	-26,1 ± 0,1	12,120 ± 50	12,134–12,056	12,154–11,860
**MAMS 36030** **	6c	E5c	bone	-35,6	10,690 ± 92	10,796–10,672	10,874–10,631
**MAMS 36034**	6c	E6a	bone	-12,8	12,120 ± 50	12,134–12,056	12,154–11,860
**KIA 55936** *	6c	E6a	bone	-15,8 ± 0,3	12,180 ± 45	12,182–12,090	12,266–12,052
**MAMS 36035**	6c	E6a	bone	-20,7	12,770 ± 50	13,354–13,197	13,496–13,116
**KIA 55935** *	6c	E6a	bone	-13,9 ± 0,3	12,800 ± 50	13,400–13,220	13,551–13,162
**MAMS 36036**	6c	E5c	bone	-22,0	12,230 ± 50	12,249–12,114	12,377–12,086
**KIA 55934** *	6c	E5c	bone	-15,4 ± 0,4	12,210 ± 45	12,213–12,110	12,361–12,081
**MAMS 37990**	6c	E5d	bone***	-20,1	12,180 ± 30	12,153–12,100	12,217–12,071
**MAMS 37991**	6c	E5d	bone	-27,3	12,740 ± 30	13,304–13,188	13,362–13,127
**MAMS 37992**	6c	E5d	bone	-23,3	12,540 ± 30	13,063–12,889	13,132–12,859
**MAMS 37993**	6c	D5d	bone	-20,9	12,260 ± 30	12,258–12,142	12,372–12,122
**KIA 55939** *	6c	D5d	bone	-16,0 ± 0,4	12,280 ± 45	12,331–12,151	12,410–12,116
**MAMS 43641**	6c	D5d	charcoal	-24,0	8,867 ± 28	8180–8112	8216–7940
**MAMS 43642**	6c	E5d	charcoal	-24,0	48,500 ± 770	50,571–48,046	47,717
**MAMS 43645**	6c	E6b	charcoal	-21,9	11,461 ± 36	11,417–11,352	11,497–11,338
**MAMS 43646**	6c	F6a	charcoal****	-26,5	10,341 ± 32	10,158–10,053	10,251–10,017

* Kiel second dating of the sample above

** low collagen content, contamination not excluded (letter by R. Friedrich, 03.07.2018)

*** cf. Fig 15.

**** floated charcoal spangles from sediment 6c

**Fig 16:**
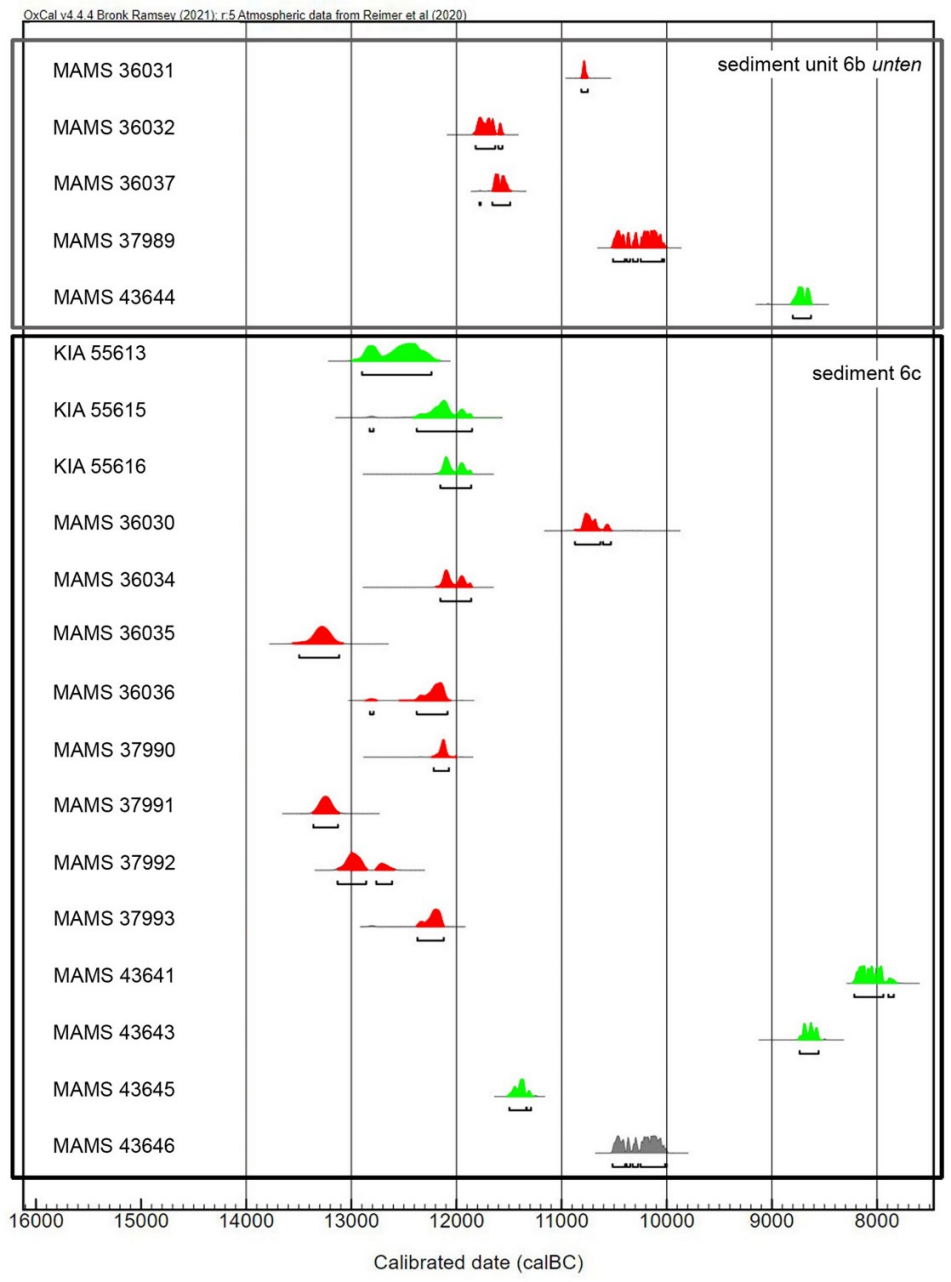
Calibration of AMS-^14^C ages with OxCal v.4.4.4 for sediment unit 6b *unten* and sediment 6c. red: bones, green: charcoal, dark grey: floated charcoal spangles.–Graphic: LWL-AfW Olpe/D. Riemenschneider.

Surprisingly, however, practically none of the age values correspond in any way to the expectations based on the archaeological (see below) and stratigraphic-sedimentological (see above) facts. Lateglacial / Early Holocene ages for sediment unit 6b *unten* and sediment 6c throughout showed significantly higher ^14^C-age values and were inconsistently too old overall (Fig 16). According to statements from the laboratory (Susanne Lindauer and Ronny Friedrich), the bone collagen analysed was basically good and sufficiently transmitted, the data quality was consistently good and technically flawless.

Only one bone fragment could be dated within the expected time range GS-1 (another bone sample from 6b *unten* and 6c yielded age values from the transition from GI-1 to GS1): a diaphyseal fragment from the excavation quarter E 7a yielded an age of 10,344 ± 25 BP MAMS 37989). This bone is from the transition zone of the 6b/8 sediments (i.e. 6b *unten*, the stratigraphic equivalent of the 6c sediment if present). However, this age value is questionable in view of the background of the other bone data.

It has to be noted that the mass of sample ages of bones measured in Mannheim from the sediment unit 6b *unten* or sediment 6c were consistently 1,500–2,500 ^14^C-years older than the expected time range "Younger Dryas / Earliest Holocene". Age inversions could also be determined: Two bones were dated from the excavation area E 6a from sediment 6c at a vertical distance of about 6 cm. The one located higher yielded a ^14^C-age of 12,770 ± 50 BP (MAMS 36035), the stratigraphically lower one "only" 12,120 ± 50 BP (MAMS 36034), thus there was a deviation of at least 650 ^14^C-years. This phenomenon cannot be explained by problematic δ^13^C-values either (Table 2), and the collagen values were also within the normal range.

In order to rule out an unrecognisable laboratory effect, five bone samples were second-dated in 2020 in the Leibniz Laboratory for Age Determination and Isotope Research at Kiel University. In the course of this, it was confirmed that the Mannheim age values had all been duplicated (Table 2), so a laboratory effect could be ruled out.

Subsequently, two more steps were taken to get closer to a solution to this "^14^C-phenomenon". First of all, charcoal fragments were selected from the basal part of sediment 6b and from sediment 6c in order to date them. However, the fact that only very few larger fragments could be extracted proved to be problematic. Five samples each from the excavation campaigns in 2018 and 2019 were separated and dated in Mannheim and Kiel. In the process of this, very different results were obtained (Table 2). Coming as a complete surprise was the Upper Pleistocene age of over 45 kyr BP (MAMS 43642) for a sample from the excavation area E 5d (sediment 6c). Two further samples from sedimentary unit 6b *unten* and one from 6c gave Early Holocene ages and another piece of charcoal from excavation area E 6b (sediment 6c) again–as for most bones–had an age within GI-1 (MAMS 43645). Out of the Kiel series (all samples from sediment 6c), two samples could not be dated (insufficient amount of charcoal), the remaining three yielded high ^14^C-ages between 12,120 and 12,385 BP.

As an interim result, the following can be stated: In the area of interest here, both older (Upper Pleistocene) and younger (Boreal) charcoal samples were evidently deposited, whereas other charcoals revealed ages that were too high, which were also obtained accordingly for the vast majority of bones.

As a final step, a larger sample of sediment 6c was sent to the Mannheim ^14^C-laboratory in 2019 and finely dispersed charcoal extracted to be used for dating. The result (MAMS 43646) with 10,341 ± 32 BP (δ^13^C -26,5‰) fell within the time range expected for stratigraphic and archaeological reasons (about mid GS-1) and duplicated the ^14^C-age of the above-mentioned bone fragment from E 7a (MAMS 37989).

Our findings regarding the radiocarbon datings can be summarised as follows:

1. In respect of the sediment unit and sediment of interest, 6b *unten* and 6c, only one animal bone fragment could be dated to the expected Younger Dryas / earliest Holocene time range.
2. The individual charcoal pieces from these units could not be dated to the expected period and consequently secondary interferences have been documented.
3. Only the "sediment age" of charcoal spangles silted from sediment 6c fell within the expected time range; however, this age should also be considered to be a mixed switch and is therefore not "absolute" (i.e. it is most likely that it points to the second half of GS-1 / Younger Dryas).
4. There is currently no explanation for the fundamentally much older age range and at the same time different old data regarding bones. It can be ruled out that bone fragments of different ages were stored in the find horizon and (almost exclusively) only these were subsequently dated. In addition, the bones that were dated were not noticeably badly abraded and sharp ridges were transmitted to the diaphyseal fragments. The collagen content was consistently good.
5. It is evident that there is a close connection between the bone finds and the lithic assemblage, with the latter (see below) and the stratigraphic position directly below Mesolithic / Early Holocene find horizons making it extremely likely that human activities were dated to the “Younger Dryas / earliest Holocene” period.
6. Regarding the sediments, bioturbate influences cannot be ruled out (and are also proven by some charcoal data). However, the documented interferences are rather small–not taking into account the bone data for the reasons mentioned–and understandably relate to smaller charcoal fragments. Larger crotovines end above sediment 6c and could be well delineated.
7. As an explanation for the discrepancy in dating of most of the animal bones (and some charcoal pieces), unknown contamination phenomena due to storage conditions in sediments with dolomite debris should be considered. Recently, it turned out that the formation of calcareous sinter apparently had a major influence on the radiocarbon dating results of two wooden iron tool handle remains found in a *Sauerland* medieval iron ore pit in dolomite rock. Thus, each measurment sample (likewise from the Mannheim AMS laboratory) yielded Upper Palaeolithic age values [136, pp. 134–135]! This example could be seen as a parallel to the problem described for the radiocarbon measurements of the *Blätterhöhle Vorplatz*; moreover, this could also have consequences for comparable sites, which would therefore also have to be examined.

It follows from the above that the archaeological remains recovered from the sediment unit 6b *unten* and especially from sediment 6c can be archaeologically dated to the middle / second half of the Younger Dryas; whereas geochronometric methods yielded conflicting results.

### Archaeology of the final palaeolithic at the *Blätterhöhle* site

#### Previous scattered pre-Mesolithic finds from the entrance area and cave

In the first years of the archaeological excavations, there had already been indications of pre-Mesolithic human presence from the *Blätterhöhle* as well as from the entrance area based on lithic artefacts [81] and a Pleistocene ^14^C-age on charcoal. However, these finds all come from disturbed or uncertain contexts.

In 2009, by excavating within the former rock shelter, a birch charcoal was recovered from a higher niveau of sediment 6b and dated to 10,981 ± 40 BP (COL 1448) and thus to the end of the GI-1. This charcoal originates from a disturbance and was probably transported up from lower-lying sediments by bioturbation.

From disturbed sediment there also came a larger end scraper with opposite burin modification, which was discovered in the course of the excavations in 2008 (sediment 3). Typologically, this artefact predates the Mesolithic finds from the same sediment. Then, in 2013, a backed point fragment was recovered from sediment 6b, which for the first time pointed to a Final Palaeolithic use of the area. From the cave itself, three back-retouched artefacts (which were only washed out later) came from the 2004 speleologically recovered sediments, which point to a Final Palaeolithic roaming of the area; these pieces were supplemented in 2019 by a small curved backed point or “Federmesser”, which was recovered in a bioturbat claimed sediment [87].

It was, however, only through the excavations from 2016 to 2021 that an extensive Final Palaeolithic inventory could be excavated, which provided some unexpected insights regarding this epoch in our region.

#### The final palaeolithic lithic assemblage(s) from the *Blätterhöhle* entrance area

In 2016, an undoubtedly pre-Mesolithic projectile form made of white patinated flint was recovered for the first time from a secure context; it came from sedimentary unit 6b *unten* of excavation square E 5a and thus from the northwest corner of the excavation area [84] (cf. Fig 19, 1). The distinctive grey colouring of the sediment 6c only began a few excavation cuts later. A clear massing of finds–in addition to significant silex artefacts, also animal bone fragments and non-local, often slightly platy river pebbles (sometimes with clearly recognisable signs of use)–then only appeared within sediment 6c below. A brief overview of the silex artefacts is given by the following, subdivided according to the sediments or sedimentary units 6c, 6b *unten*, 6b/8 and 8.

**Fig 17:**
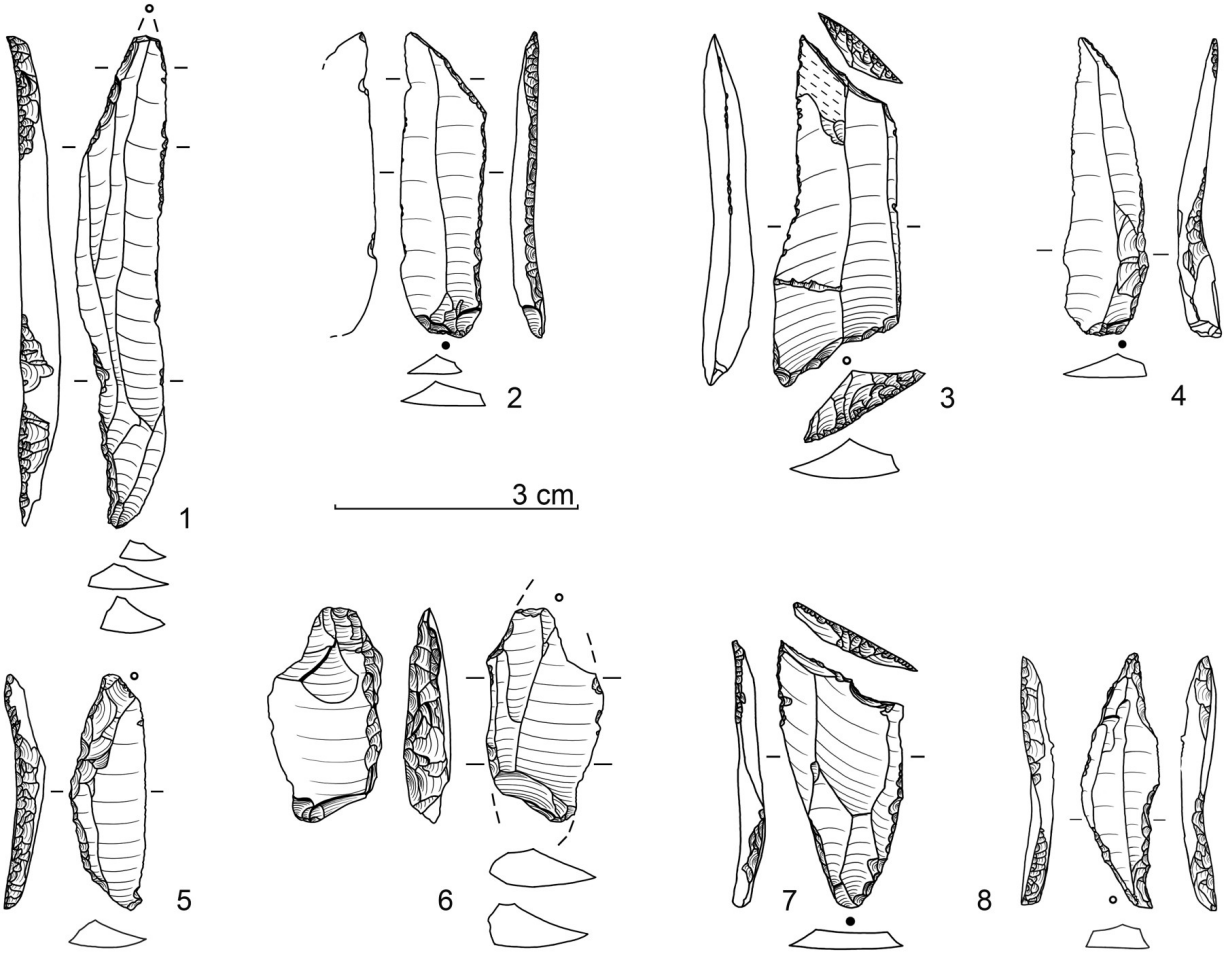
Lithic implements from sediment 6c (all white patinated flint). For a more detailed description see text.–Graphic: LWL-AfW Olpe/A. Müller.

**Fig 18:**
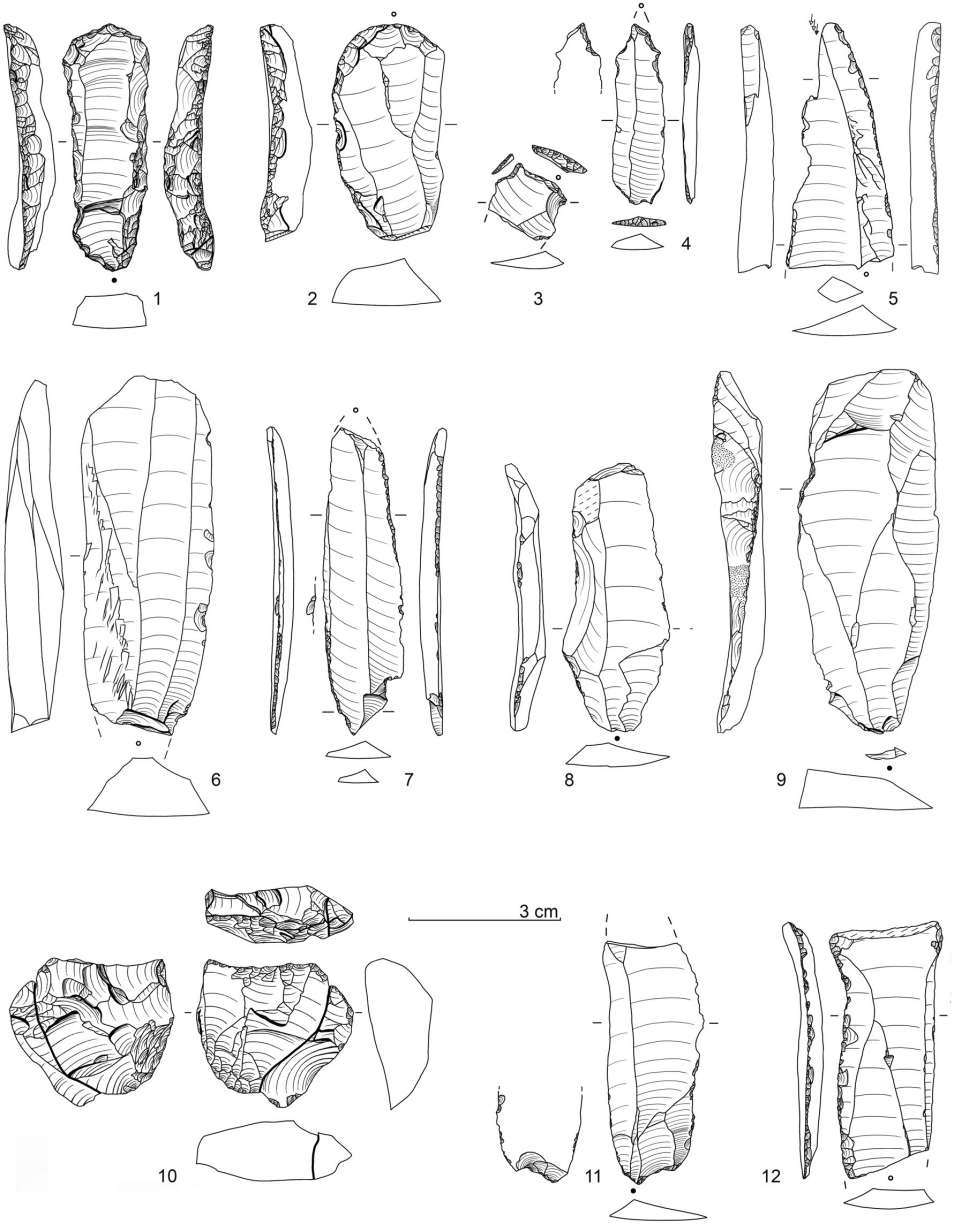
Further lithic implements from sediment 6c (all white patinated flint). For a more detailed description see text.–Graphic: LWL-AfW Olpe/A. Müller.

*Sediment 6c*. 476 lithic artefacts (Figs 17 and 18)

Typologically, the lithic assemblage is characterised by mostly larger, elongated backed points (or knives) and their fragments (Fig 17). The backed pieces are extremely varied and peculiarly individual. What was striking was the recovery of an angle-backed point with a fine, almost marginal edge retouch (Fig 17, 2), as well as a blade with double oblique truncation with no backing of an edge (Fig 17, 3). Still present is also a long, narrow point with a partially backed edge, which has a slightly kinked point section (with a missing tip) and fine splintering on the other longitudinal edge (Fig 17, 1). In addition, it is worth mentioning a small *Federmesser* (curved backed point; Fig 17, 5) and a slightly backed point with a fine tip retouching on a neo-crested blade (Fig 17, 4). The medial fragment of a further angle-backed point shows a strong, partially bilateral backing as well as heavy impact fractures at both ends (Fig 17, 6). Other backed pieces have survived only in fragmentary form.

Two other points are also interesting: a broad, simple point (“Zonhoven point”) with an obtuse-angled end retouching and some retouching of the edges (Fig 17, 7), as well as a tanged point-like piece that was initially referred to as a double borer (Fig 17, 8). However, it should actually represent an atypical (Ahrensburgian) tanged point, and so Veerle Rots (University of Liège), despite the patination, recognised some micro-signs of wear that are characteristic of projectiles, which supports the description as a handle tip (V. Rotts, letter 2017).

Other implements include end scrapers on edge retouched blades (Fig 18, 1 and 2), fine borers (Fig 18, 3 and 4), burins (Fig 18, 5), and retouched blades (Fig 18, 7) and flakes. Among the debitage, large, wide blades (Fig 18, 6. 8. 9. 11 and 12) and flakes, some with chipped/retouched edges, are significant. Only one heavily used core has been found on which one elongated flake could be refitted (Fig 18, 10). The blades and bladelets mostly show parallel/subparallel dorsal ridges and edges and indicate a dominant unipolar knapping strategy.

#### *Sedimentary unit 6b* unten

**Fig 19:**
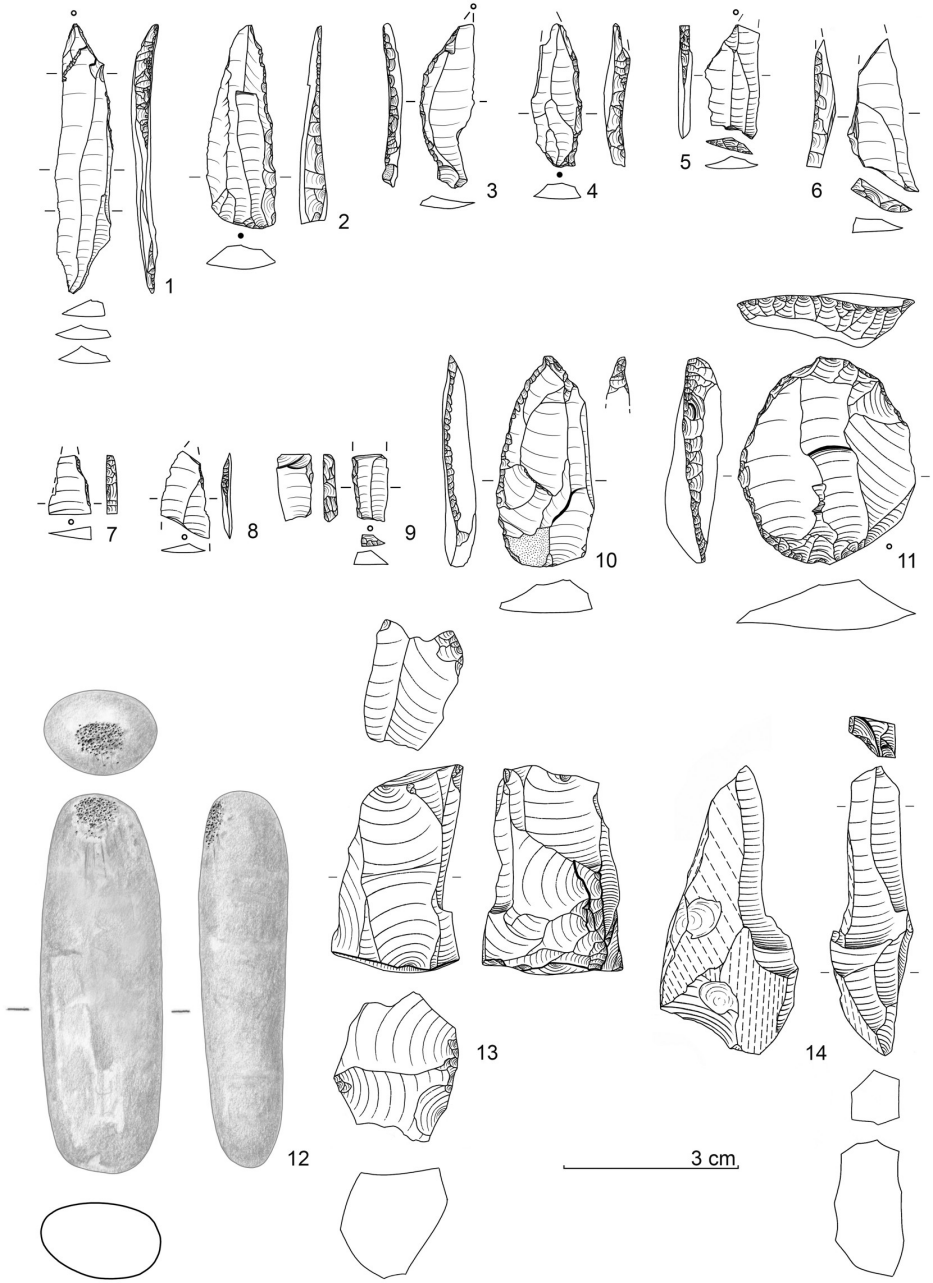
Lithic implements from sediment unit 6b *unten* (lydite: Nos. 2, 3, 6; all others: White patinated flint) and a retoucher from an elongated river quartzite pebble. For a more detailed description see text.–Graphic: LWL-AfW Olpe/A. Müller.

131 lithic artefacts (Fig 19)

The first backed point found, as already mentioned, is only partially backed on a narrow straight blade (Fig 19, 1). The tip, which has survived in its entirety, is slightly oblique and the base shows a continuous fine edge retouch. There are also several small *Federmesser*-like pieces present (Fig 19, 3 and 4), as well as various fragments of backed points (Fig 19, 7 and 8). Remarkable is the recovery of a broad point with an almost straight, continuously retouched back (Fig 19, 2) as well as a fragment of a backed point with an oblique retouched base (Fig 19, 6) reminiscent of Malaurie points. The inventory is supplemented by the fragments of a basal retouched narrow “Zonhoven point” (Fig 19, 5), and a truncated narrow backed bladelet (Fig 19, 9).

As late as 2021, two cores were recovered from this sediment unit, a burin-like piece on a flint frost shard (Fig 19, 14) and a cubic piece that was finally knapped by using an “anvil” (Fig 19, 13). In addition to an almost completely edge retouched, larger end scraper on a broad flake (Fig 19, 11), there are also a borer (Fig 19, 10), and edge retouched/splintered blades or bladelets present. In addition, a small, long, narrow river pebble of quartzite shows clear traces of having been used as a retoucher (Fig 19, 12).

**Fig 20:**
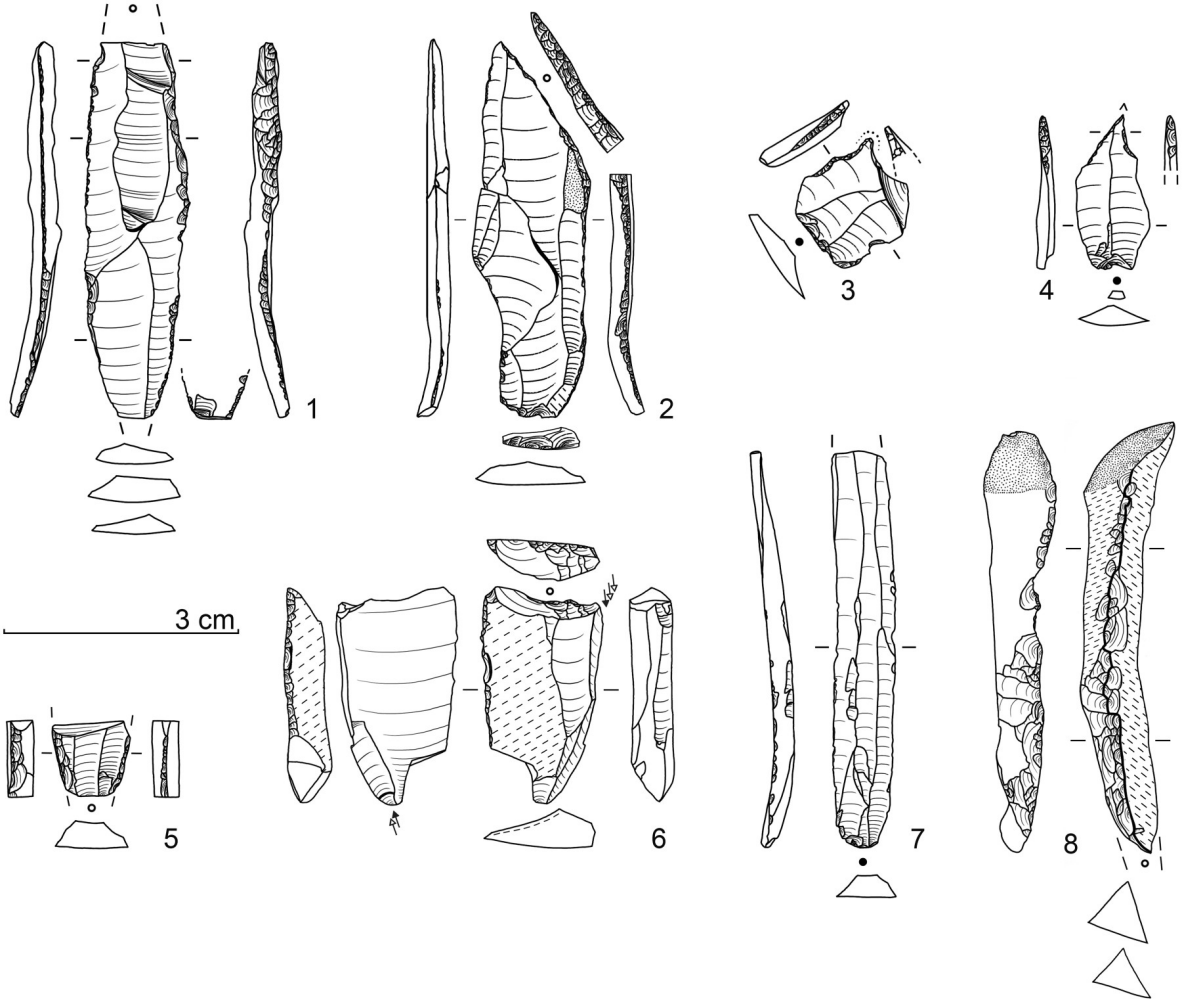
Lithic implements from sediment 8 (no. 5) and sediment unit 6b/8 (all others) (all white patinated flint). For a more detailed description see text.–Graphic: LWL-AfW Olpe/A. Müller.

*Sedimentary unit 6b/8*. 117 lithic artefacts (Fig 20).

This sedimentary unit delivered the fragment of a long, narrow backed point with one edge partly backed (Fig 20, 1), a nearly complete angle-backed point (or “knive”?) with basal retouch (Fig 20, 2), as well as several small fragments of backed pieces. There are also fine borers (Fig 20, 3 and 4) and a double burin (Fig 20, 6) as well as retouched blade fragments included. Worthy mentioning is that among the debitage, there is a very narrow and regular proximal fragment of a bladelet (Fig 20, 7), as well as an elongated primary crested blade (Fig 20, 8).

*Sediment 8*. 5 lithic artefacts (Fig 20, 5)

Only five lithic artefacts could be assigned to this sediment. Of limited significance is a medial bladelet fragment with a clearly and a marginally retouched edge.

#### The lithic raw materials used from all sediments/sediment units

The lithics mentioned are made basically on two major raw material units: siliceous slate (lydite), which occurs in the region, and cretaceous flint. In the siliceous slate several workpieces could be identified as on the basis of colour and texture [88]. The flint could mainly be assigned to the erratic Baltic variant occurring regionally in moraine deposits and gravel. Eolised rock surfaces, moraine cortex, occasionally rich bryozoan inclusions and transparency (if not patinated) could be identified. However, several workpieces could also be distinguished in the flint [88]. Due to a different texture or lithosphere, small proportions of "Western European / Meuse" flint variants cannot be ruled out as coming from the Lower Rhine or Meuse region further to the west. The distances are around 50 km and more to the west and would not be unusual in this context.

#### Profile projections in the lithic assemblage(s)

**Fig 21:**
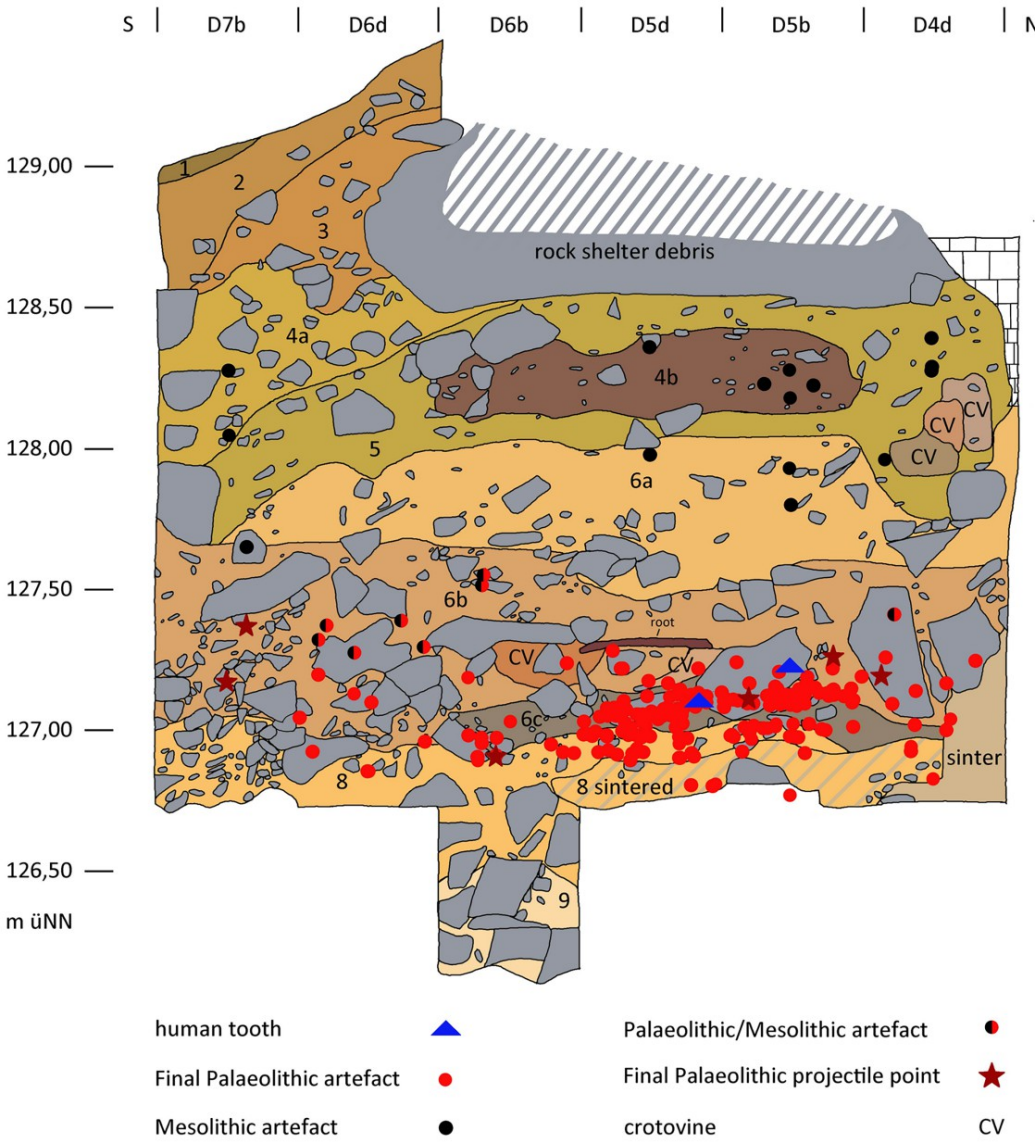
Projection of final palaeolithic and mesolithic single recorded lithic implements (n = 170) and human remains on the (assembled) NS profile DE (cf. Fig 10). All finds from the directly adjacent half square meters.–Graphic: LWL-AfW Olpe/D. Riemenschneider.

**Fig 22:**
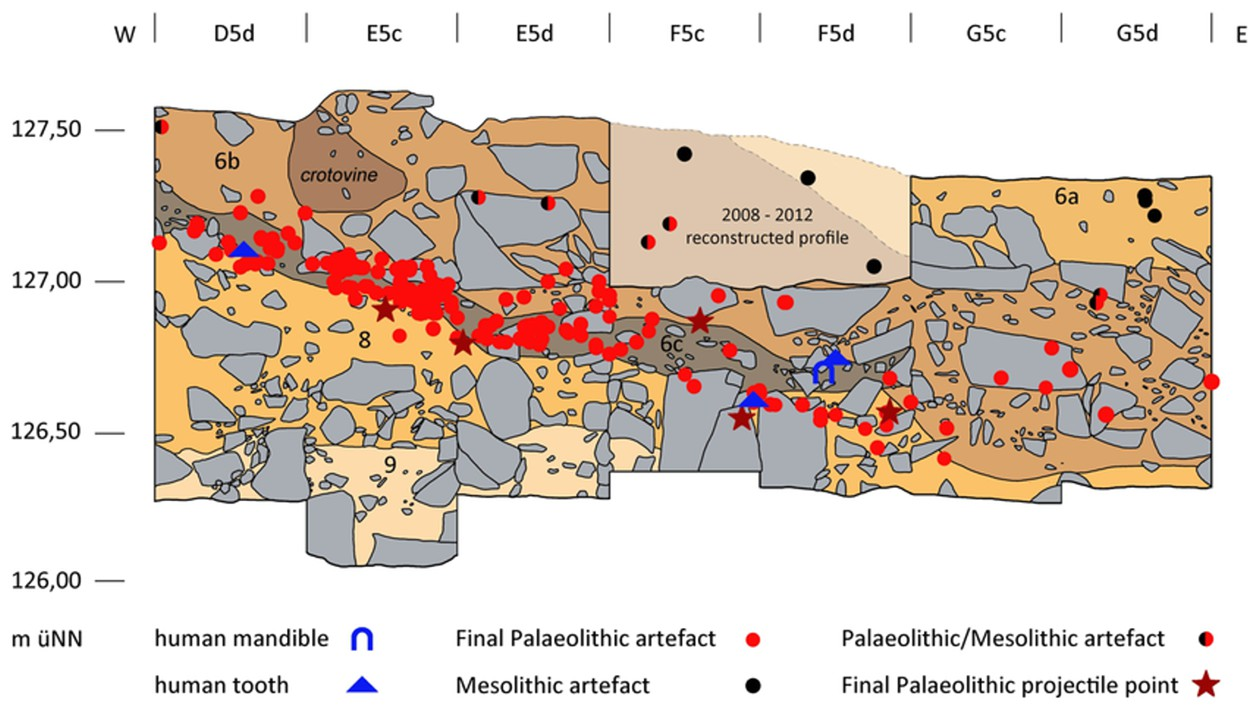
Projection of final palaeolithic and mesolithic single recorded lithic implements (n = 167) and human remains on the WE profile 5S (cf. Fig 10) showing only the basal sediments/sediment units. All finds from the directly adjacent half square meters.–Graphic: LWL-AfW Olpe/D. Riemenschneider.

In order to clarify the question of whether the Final Palaeolithic silex inventory of the various sediments/sedimentary units represent one coherent assemblage, several find projections were made on the profiles. Due to the sometimes stronger dip of the sediments to the south and east, we only projected the lithic finds from the quarter of the respective excavation squares directly adjacent to two selected profils [cf. 88]. Due to the abundance of finds, the western area of the excavation area is deemed to be the most significant, therefore, the NS-profile DE (Fig 21) and the WE-profile 5S (Fig 22) are presented in more detail (cf. Fig 10).

Both profile projections demonstrate clearly, that the bulk of the silex finds can be assigned to sediment 6c. The projections make it clear that this sediment is to be evaluated as a ‘closed find layer’. The finds assigned here during the excavation to the hanging or lying sediments can with some justification also be assigned to this layer of finds and are to be considered as having been severed by forced post-sedimentarily.

To the south or east respectively, where the grey colouring of sediment 6c disappears, the pieces are more vertically warped and scattered much more loosely. The formerly closed find layer here has obviously been more scattered and broken up due to stronger post-sedimentary processes outside the rock shelter.

As the profile projection and structure make evident, the find-rich sediment 6c is only preserved in the area below the large overhanging block–the former rock roof. 6c represents a rich find layer or archaeological horizon. On the outside (to the south and east) of the collapsed block or the rock roof, the find layer was processed, the finds were dispersed more vertically. Although it cannot be ruled out that further short-term settlement events also took place after the formation of the find layer in connection with sediment 6c (found primarily in sediment unit 6b *unten*), these cannot be separated due to the post-sedimentary processes described (however, so far no lithic refits have been achieved between the sediments after some 60 hours working on this by one of us [W. Heuschen]). Hence, the described lithic artefacts of the sedimentary units 6b *unten*, 6b/8 and sediment 6c may so far be understood as a single assemblage, all the more so as the characteristic tools do not currently justify a typo-technological differentiation.

#### Classification of the lithic assemblage(s)

The inventory of sediment 6c and its equivalents found below the Mesolithic horizons in the entrance area of the *Blätterhöhle* as well as some higher-lying finds that were probably post-sedimentarily relocated, provide evidence of a Final Palaeolithic use of the site. Typo-technologically characteristics of the assemblage are, on the one hand, larger blades and bladelets obtained in a unipolar manner, as well as numerous backed pieces (projectiles and “knives”), which show great variability. In our region, such forms are generally rather characteristic of the GI-1 (late Meiendorf and Allerød Interstadial) and have been widely documented within this period (see above).

However, due to its stratigraphic location directly below the Mesolithic find horizons / Holocene sediments and the ^14^C-age of about 10 kyr cal BC obtained from charcoal spangles from sediment 6c there are substantial indications at hand that the archaeological remains are chronologically considerable younger than GI-1. (This is clearly preferred–as discussed above–despite the numerous ^14^C-ages obtained on bone and charcoal which cover a much older time-frame–late GS-2 / early GI-1.)

Subsequently, in front of the *Blätterhöhle* a silex inventory was recovered which is characterized by variable backed pieces, large regular blades and bladelets which dates to (the second half of?) GS-1 / Younger Dryas. For this period in our area it was previously the case that Ahrensburgian sites with small tanged points and numerous reindeer remains were very much to be expected [9, 49, 53]–but this is not the case here.

Looking beyond our region but the period considered, techno-complexes based on backed points are typical for southern Central Europe [137] / Southern Europe and Western Europe [42]. Especially in France, these inventories are characterised by different types of backed points, including Malaurie points.

In France, the Younger Dryas backed points inventories are subsumed under the term “Laborian” (*Laborien* or *Laborien ancien*), followed by a Late Laborian (*Laborien recent* or *Épi-Laborien*) towards the end of the Younger Dryas / transition to the Holocene [5, 138] (cf. Fig 2). Here, several categories of lithic artefacts similar to those present in front of the *Blätterhöhle* are present:

- different backed point forms
  - straight backed
  - curved backed (*Federmesser*)
  - partially backed
  - basally truncated backed
- "tanged point" / tanged point-related
- simple micropoint (“Zonhoven points”)
- blade/bladelet knapping concepts
- blades/bladelets with a straight longitudinal section and flat cross-section

Both the characteristically narrow pointed backed bladelets ("Blanchères points") and the "bitruncated trapezoids" [139] are absent in the assemblage considered here which contradicts a closeness of our *Blätterhöhle* inventory to the Late Laborian. Both lithic implements are currently widespread in France up to the Seine and Rhône [cf. 44], but isolated Blanchères points have been recoverd even further apart as Ghent in Belgium [140] and Avington VI in southern England [58]. The "^14^C-sedimentary age" of 10 kyr cal BC further supports the archaeological proximity of our inventory to the preceding Laborian, as there are corresponding ^14^C-ages for this [cf. 43, 44].

In the Laborian/Epi-Laborian, variable backed lithic implements are typical, including Malaurie points. As specified earlier, in the *Blätterhöhle* assemblage likewise numerous backed pieces are present including one fragmented, basally truncated backed piece (Fig 19, 6) which can be placed alongside Malaurie points. (As mentioned in the introduction, however, Malaurie points are generally present in the Rhineland and Westphalia.) Furthermore, tanged or tanged-like points (cf. Fig 17, 8) and Zonhoven points (cf. Figs 17, 8 and 19, 5) are also linking elements; for the Late Laborian comparable lithic forms are interpreted as being an influence by the “northern” Ahrensburgian (therefore sometimes assigned as “Épi-Ahrensbourgien” [138] where numerous small tanged points are typical.

To sum up: from the basal sediments reached so far at the entrance area of the *Blätterhöhle*, a silex inventory has been recovered that, due to its typological characteristics, was previously completely unknown for our region and beyond. However, it shows a clear proximity to the French Laborian/Late Laborian technocomplex(es) and, after weighing up all the facts, dats within the same time frame of GS-1.

It is remarkable that among the bone fragments recovered so far at the *Blätterhöhle*, the reindeer does not appear abundantly. This is somewhat unusual for a Younger Dryas-period site in our region. Rather, a "warm" fauna seems to predominate (as is also known in the *Laborian ancien*/*Laborien recent* [141])–but, however, the determinable faunal remains are rare so that further analyses (and significant finds) are necessary.

Basically, however, it needs to be stated that if we evaluate the archaeo-stratigraphic situation according to the facts presented, there is a Final Palaeolithic inventory of the (middle) Younger Dryas/GS-1 period on the entrance area to the *Blätterhöhle* situated below Holocene sediments, which is not characterised by Ahrensburgian tanged points and numerous reindeer remains, but by backed points with red deer remains. This circumstance clearly points to western and southern regions and is a hitherto new and unexpected result for our region and those regions adjacent.

### Site formation

According to the micromorphological analyses, the initial sediment for our sediment 6c and its base (sediment 8) is a primary loess, which is not to be classified in GS-1 but in GS-2. The high proportion of debris, partly in the form of larger blocks, and perhaps also some of the identifiable loess snails clearly indicate a cold climate at the time of formation.

Based on the chronological classification of sediment 6c discussed above and the inventory of finds contained therein, there must be a larger hiatus between the formation time of the loess and human settlement. Sediments from the GI-1, especially the Allerød Interstadial, have not survived or were not detected. If one does not consider the bone and charcoal finds from the find horizon, some of which are significantly older, as relics of these sediments (dated between approx. 13,3–11,4 kyr cal BC; see discussion above), then at the beginning of GS-1 the GI-1 sediments and their contents have been completely cut and removed. This is also supported by the fact that sediment 6c represents an anthropogenic overprint of the upper part of the much older, GS-2 temporal loess (see the contribution by M. Kehl).

Later on, early Holocene sediments were quickly deposited, which prevented the remaining Final Pleistocene sediment(s) from being cleared out. But on the outside of the rock overhang, which probably broke during the Atlantic, intense postdepositional processes remained until the younger Holocene. Here, the find horizon has been cut off and incorporated into younger sediments. A complete Holocene overprinting (e.g. by soil formation processes) of the sediment 6c did not take place but some parts of the Pleistocene sediment along with its archaeological content may have been transferred to the forming sedimentary unit 6b *unten*.

### Human remains

**Fig 23:**
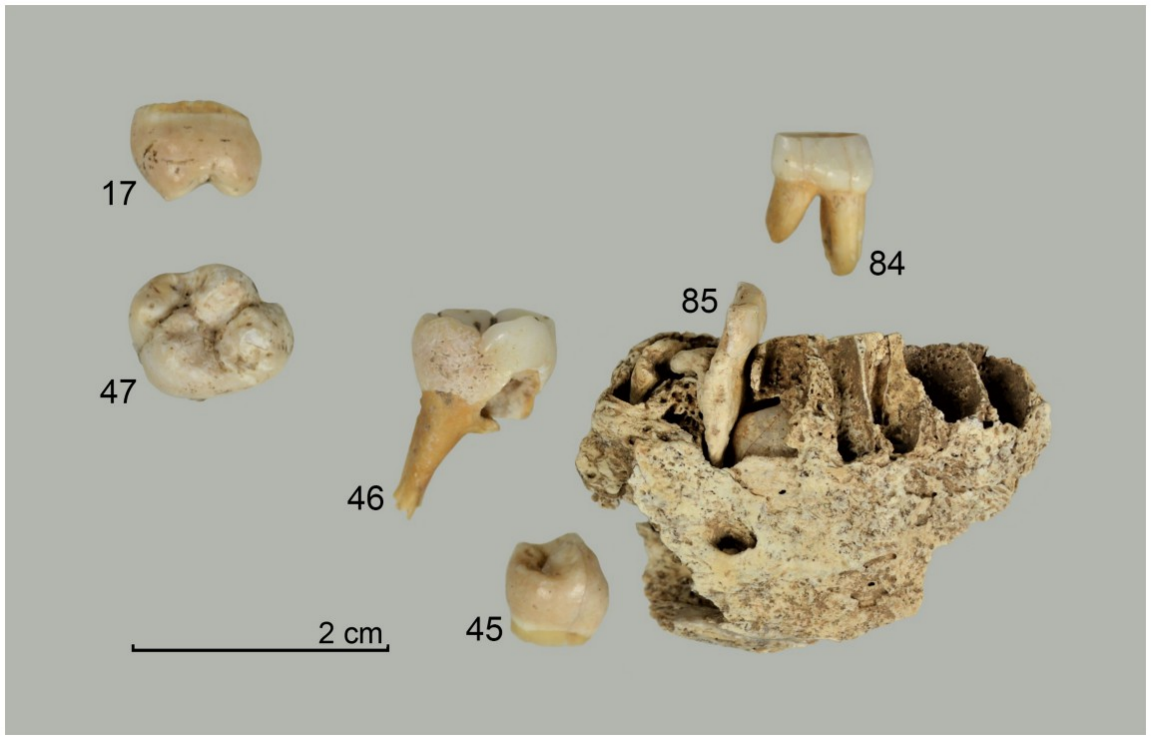
Final palaeolithic tooth and mandible fragments of a child of about 7 years. Single tooth finds: unerupted molar M2 (tooth germ, 47?), unerupted molar M2 (tooth germ, 17?), molar M1 (46) with only a half root, complete deciduous molar m1 with two roots (84); the right sided fragment of a mandible with fitting halved deciduous molar m2 (85) and a tooth germ of the PM2 (45) with a fully formed crown and root base. Further erupting permanent teeth are visible in the mandible (cf. Fig 27).–Photo: J. Orschiedt, LWL-AfW Olpe/A. Müller.

In the course of the 2018 excavation campaign, surprisingly the first human remains were discovered in the Final Palaeolithic sediments, namely four teeth (the details of which are as follows; Table 3 and Fig 23):

1. BV 18, sq. E 5d profile alignment; most probably sediment 6c: unerupted molar M2 (tooth germ) (47?)*, the lower edge is old and damaged, development stage: crown completed with defined pulp roof (Crc) [142, 143].
2. BV 18, sq. D 4d, x 56 y 3 z 127.20; sediment 6c: molar M1 (46) with only a half root, recently damaged, development stage: rooth length completed (Rc) (Morree 1963 a, b), and wear stage 1 [144].
3. BV 18, sq. D 5b, crotovine, disturbance; unerupted molar M2 (tooth germ) (17?), damaged and only about half preserved, development stage: crown completed with defined pulp roof (Crc) [142, 143].
4. BV 18, sq. D 5d, x 79 y 43 z 127.11; sediment unit 6c/8: deciduous molar m1 with two roots (84), complete, complete, development stage: rooth length compled (Rc) with root resorption less than 1/4 [142, 143] and wear stage 3 [144].
  (* the numbers in brackets relate to the international dental nomenclature code)

**Table 3:** Preserved teeth from excavations 2018 and 2019. –Development stages and Root resorption based on modified Mooreen stages [142, 143] according to [145]: CrC—crown completed with defined pulp roof; Rc—root length completed with parallel ends; R1/4—root length less than crown length with visible bifurcation area; < 1/4 resorption of apical quarter of the root.–Wear stages according to [144]: 1—unworn; 2—wear facets, no observable dentine; 3—cusp pattern partially or completely obliterated, small dentine patches.

ID	Sediment	Tooth	Development stage	Root resorption	Wear stages
**BV 18, E5d**	6c	M2 (47?)	Crc	-	1
**BV 18, D4d**	6c	M1 (46)	Rc	-	2
**BV 18, D5b**	Crotovine	M2 (17?)	Crc	-	1
**BV18, D5d**	6c/8	m1 (84)	Rc	< 1/4	3
**BV 19, F6b**	6b/6c	Mandible fragment w. PM1 (44), C (43)	Crc	-	1
**BV 19, F6b**	6b/6c	PM2 (45)	R 1/4	< 1/4	1
**BV 19, F6b**	6b/6c	m2 (85)	Rc	< 1/4	indet. (damage)

The four teeth or tooth germs were found in the north-western excavation area, not far from cave entrance 1 and below the former rock roof (cf. Figs 10, 21 and 22). If we evaluate the two stratified recovered finds as representative for all, the teeth–a molar of the permanent dentition, a deciduous molar, and two crowns or crown fragments (so-called tooth germs)–come from sediment 6c and indicate a subadult individual in dentition. In addition to the presence of tooth germs, this age assessment is supported by the severe abrasion of the deciduous molar (m1) with simultaneous incipient degradation of the tooth root and the possibility that the root tip of the permanent molar (46) was either not yet fully developed or damaged.

**Fig 24:**
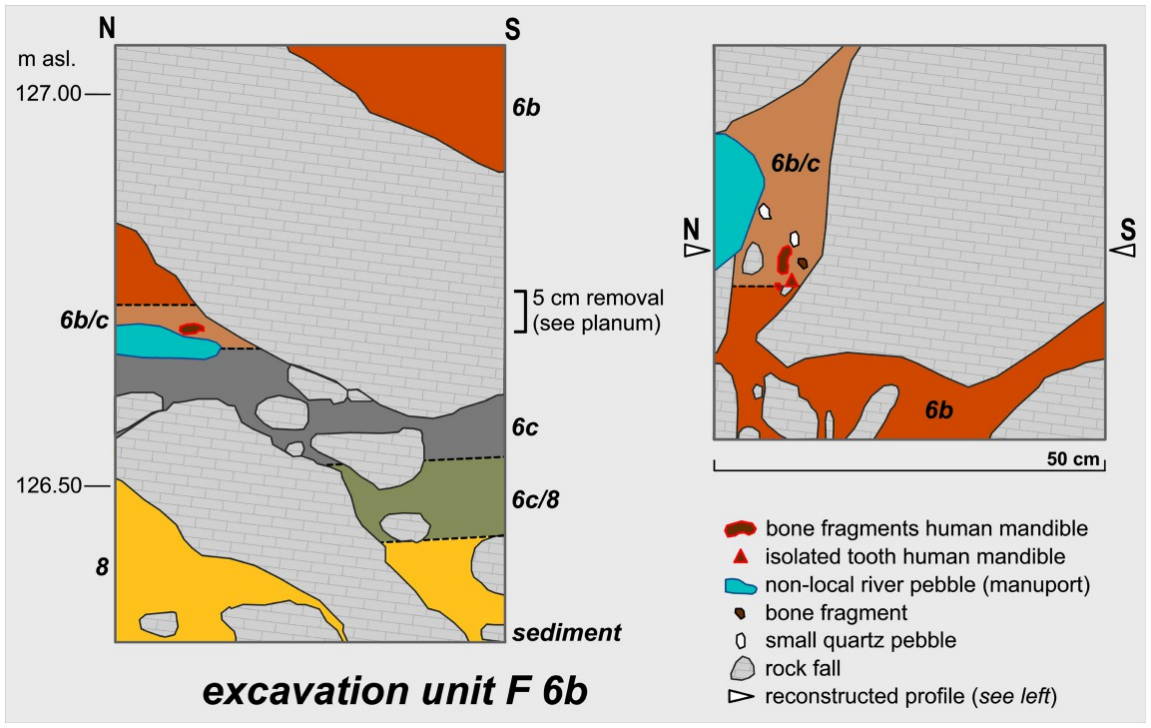
Situation of the human mandible fragment and an adjacent tooth in the same find level. Left: NS profile reconstructed on the basis of the planum documentation of the excavated square F 6b, right: top view of the find level in F 6b.–Graphic: LWL-AfW Olpe/M. Baales.

**Fig 25:**
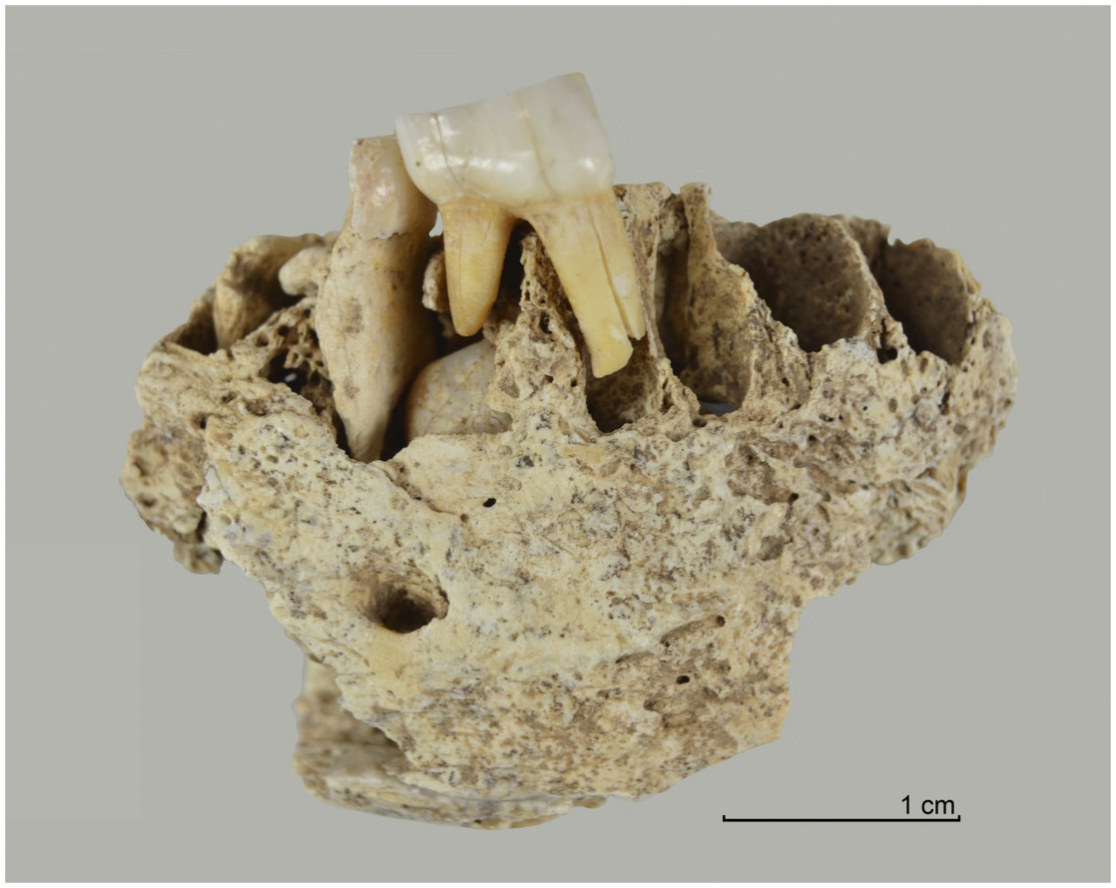
Fitting of the complete deciduous molar m1 with two roots (84) into the alveoli of the mandible. –Photo: J. Orschiedt, LWL-AfW Olpe/A. Müller.

**Fig 26:**
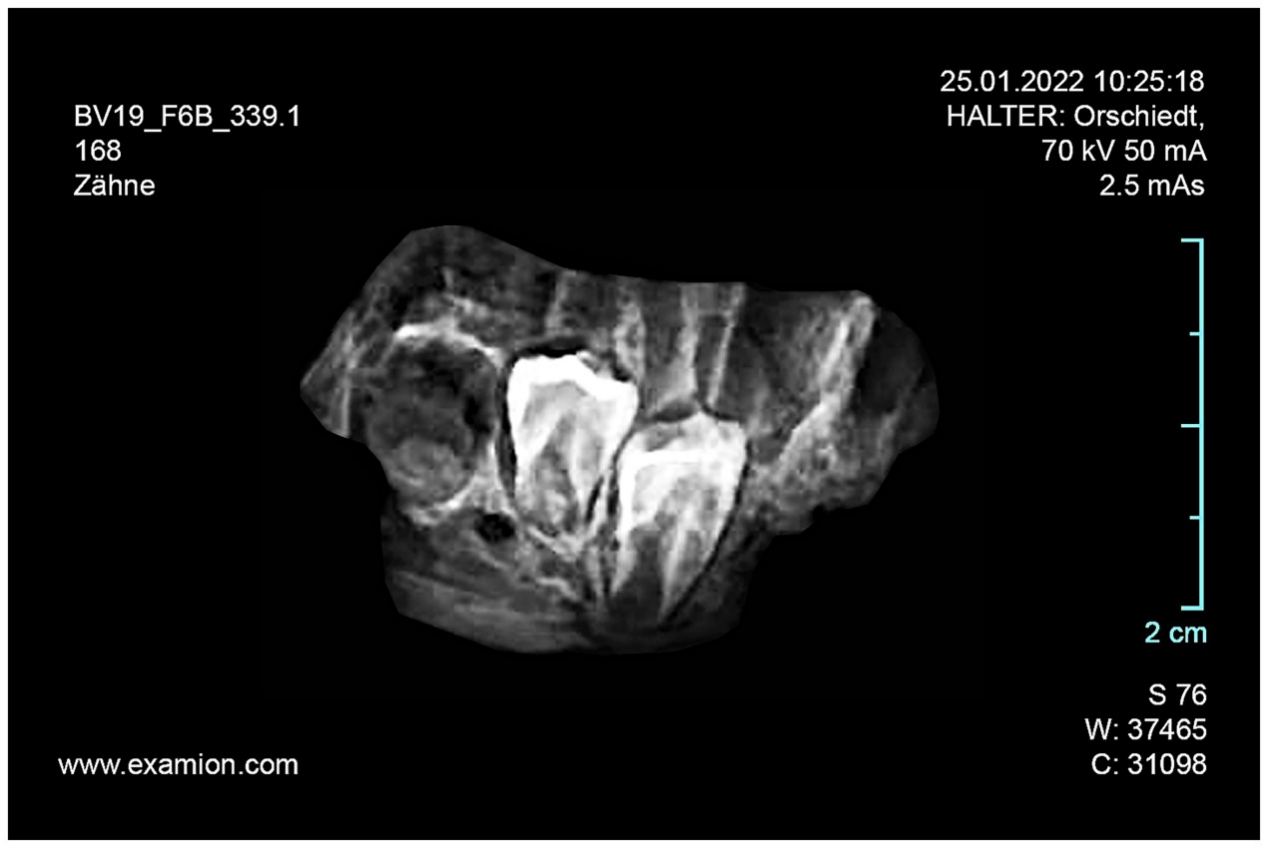
X-ray of the mandibular fragment showing the crowns of not yet erupted permanent teeth. –Photo: J. Orschiedt, LWL-AfW Olpe/A. Müller.

In the light of these first finds, it was not surprising that a year later a right-sided fragment of the body of a mandible was discovered (Fig 23) between large boulders to the east (square F 6b; x 71, y 92, z 126.70; sedimentary unit 6b/c; Fig 24). Further teeth were found in the immediate vicinity and include: a tooth germ of the permanent dentition, the PM2 (45) with a fully formed crown and root base (it could be fitted into the jaw fragment) of less than 1/4 root length (R 1/4) [142, 143] and the halved deciduous molar m2 (85) with an isolated root remnant, with a root resorption less than 1/4 [142, 143] and unidentifiable wear stage according to post depositional damage (which can also be fitted into the jaw fragment; Fig 25). The crown of the tooth germ of PM1 (44) is still preserved in the jaw, as is the tooth germ of Caninus (43) on the underside of the jaw fragment, in which only the crown is completed with defined pulp roof (Crc) [142, 143] (Fig 26).

In total, in addition to the mandibular fragment, there are two isolated deciduous molars (m1, m2), a fully developed first permanent molar and the germs of three other permanent teeth (two second molars and one premolar) present.

The lower jaw fragment lay a little to the south of a non-local pebble. Part of a large block of limestone protruded about 10 cm above the find position of the jaw (Fig 24). Below this block approximately 5–7 cm of sediment unit 6b *unten* and below about 3–4 cm of sediment unit 6b/c, in which the jawbone was located, could be documented. The material referred to as sedimentary unit 6b/c was a transitional horizon, where sediment unit 6b *unten* continuously transitioned into Sediment 6c, but on a small scale no clear boundary could be identified. Sediment 6c is approximately 10 cm thick in the area under the rock and below that, under another large collapsed block, sediment 8 came to light.

No disturbances could be observed in the near part of ​​the jaw. Sedimentary unit 6b/c and sediment 6c below the large collapsed block appeared somewhat crumbly overall in comparison. It has not been possible to clarify in the field whether this characteristic was caused by (small-scale) bioturbation or by the "protection" of the overlying, larger collapse block and the resulting reduced post-sedimentary compaction of the sediment. Types of finds that are typical for sediment 6c were found immediately below the lower jaw fragment, e.g. larger non-local boulders, lithic artefacts, bone fragments etc.

**Fig 27:**
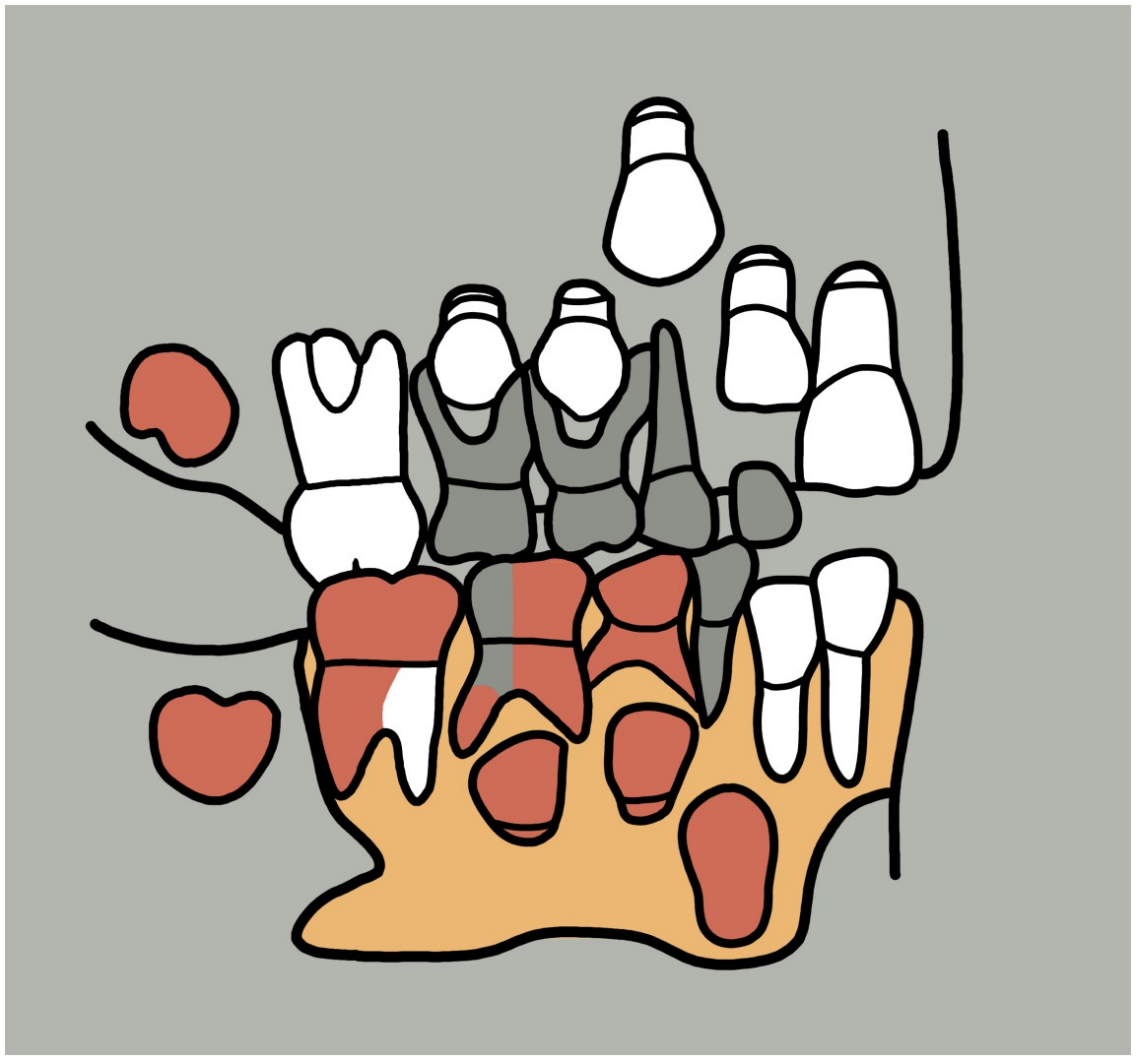
Schematic representation of the jaws (right sided view) of an approximately 7-year-old child: The permanent teeth/crowns are highlighted in white and the deciduous dentition in dark grey. The recovered mandibular fragment is highlighted in brown and the associated teeth and tooth fragments in red.–Graphic: J. Orschiedt, LWL-AfW Olpe/A. Müller.

Pertaining to the interpretation of the few human remains, the fact that the isolated deciduous molar m1 (84), found approximately 2.5 metres to the north-west a year before the lower jaw, fits perfectly into the jaw fragment (Fig 25). This would indicate that all the remains could be attributed to a single young child of the age-category Infans II (Fig 27). Overall, the isolated teeth and jaw fragment can be considered to belong to a child aged 7 years ± 24 months [146].

The find depths of the individual teeth and the jaw fragment vary greatly in terms of the absolute height values, but this is only due to the slope situation and the layers sloping both south and east at the same time. The jaw fragment, with the lowest depth value of all finds, was located well away towards the east. The piece could thus have been relocated from a higher site position with some teeth falling out in the process. It can therefore be assumed that both horizontal and certain vertical relocation processes have led to the distribution of the recovered finds.

How can this small lower jaw fragment be explained? No further clear human skeletal remains could be recovered from the excavation of the surrounding quarter of a square metre. It can therefore be assumed that the jaw was deposited here in isolation and was affected by later taphonomic processes. Rockfall could also have played a role here, as the jaw fragment (alongside damage from excavation in an area packed with rock fall debris) clearly shows signs of damage, which indicate the occurrence of a major rock fall. The jaw fragment shows old contusions of the bone substance and damage to a tooth in the same region. Since the piece was recovered from beneath a large boulder, rockfall could be assumed to have been the cause of these defects, even though the jaw was not found in direct contact with an overlying boulder.

Currently, we consider it possible that the findings are due to a disturbed burial, remains of a dead child being laid down on the surface or the introduction by carnivores from elsewhere. Exactly how the jaw fragment came to be in the find position, however, remains open to question. It is possible that the burial was (or is) further "up the slope" in a northwesterly direction, roughly below cave entrance 1, and that the jaw fragment was moved away from there, causing teeth to be lost. Alternatively, predators may have played a role in this case and brought the lower jaw fragment here from a completely different place.

**Fig 28:**
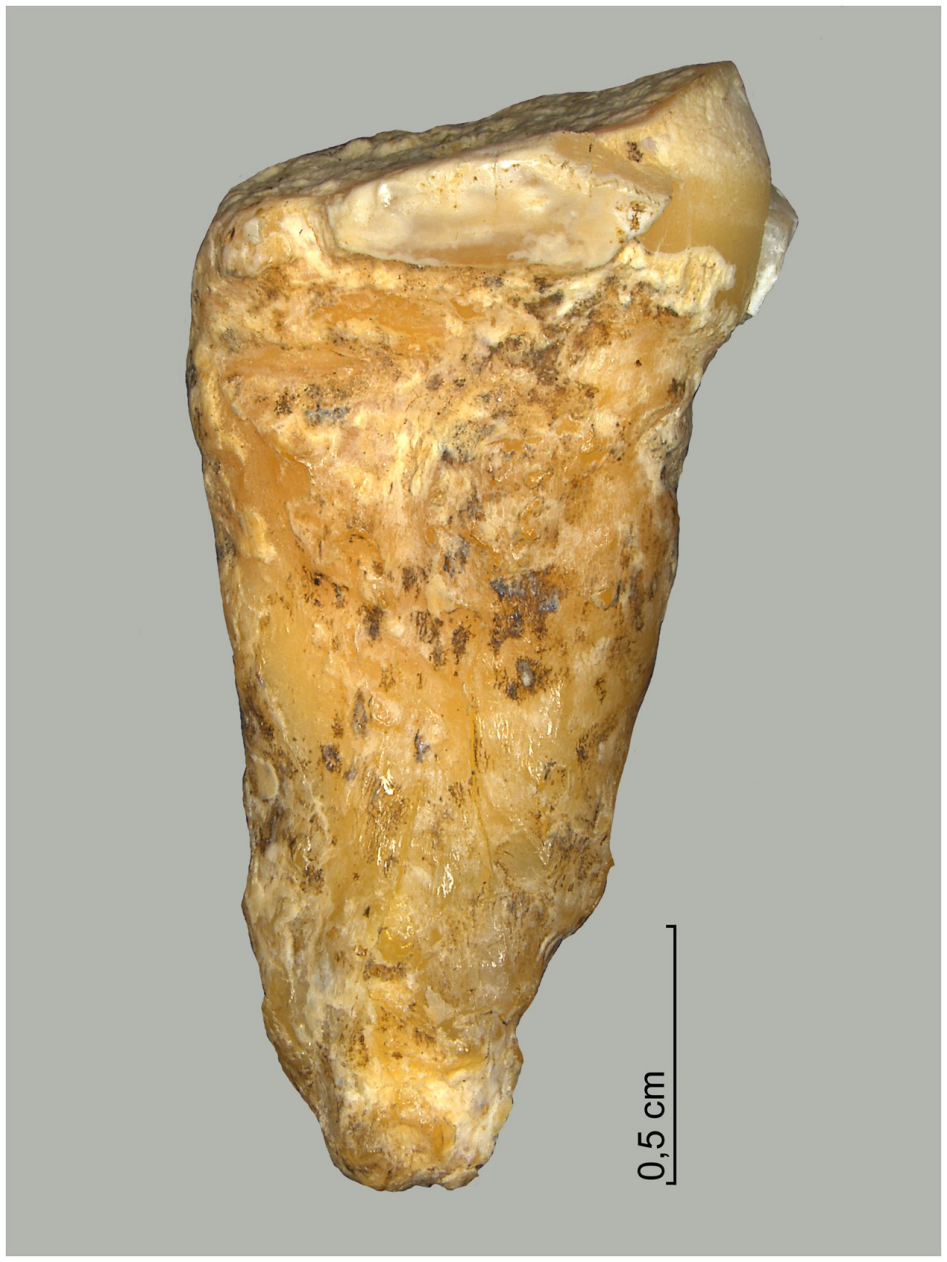
Mandibular premolar from a further, older adult human individual. –Photo: J. Orschiedt, LWL-AfW Olpe/A. Müller.

One of us (N. Nolde) identified another human tooth among the wet sieved finds (sediment 6c / square F 6a; cf. Fig 10) from 2019. It was found to be an abraded mandibular premolar from an older adult individual (Fig 28), which therefore does not belong to the child’s mandible described above. The tooth was lost postmortem; how it got into the find layer, however, must remain open (cf. above). The tooth clearly shows damage and signs of erosion and may therefore also have been relocated. Such isolated teeth are not uncommon at Pleistocene sites.

It should be noted that with the remains described here, especially the lower jaw fragment, Pleistocene human remains were recovered in an undisturbed stratum context for the first time in North Rhine-Westphalia (and neighbouring federal states of Germany) through systematic excavations. In addition, the small number of finds of Younger Dryas or Final Palaeolithic human remains in NW-Europe, and further afield, is rewardingly expanding. The taphonomic history of the mandibular fragment still remains to a great extent ‘open’ at present.

## Discussion and outlook

Below the former rock overhang in the entrance area to the “Leaf Cave”–*Blätterhöhle*–in the dolomite massif of the Weissenstein not far from the *Lenne* river near the city of Hagen in southern Westphalia (in the northern edge of the *Sauerland* uplands, southern Westphalia, North Rhine-Westphalia, western Germany), another, this time Pleistocene, sediment package with archaeological finds was documented below Mesolithic/Holocene find horizons. The grey-coloured sediment 6c is particularly striking and the emergence of this can be traced back to human activities near a former combustion feature. Sediment 6c soon disappears, but has its equivalent in other sedimentary forms with corresponding archaeological finds. It remains to be seen whether the isolated location of characteristic lithic artefacts a little further above this find horizon is the result of larger bioturbate shifts (the sediment layers also clearly dip to the east and to the south) or of somewhat more recent human occupation events. The sediments are very rich in calcareous debris including larger boulders. Nevertheless, 6c is basically a fine grained sediment, according to micromorphological results, an overprinted Upper Pleistocene loess, which is also primarily present as sediment 8 below.

The stratigraphic position of sediment 6c and equivalent sediment units (and thus also the archaeological finds preserved here) below Holocene cover layers with Mesolithic archaeological remains suggests that they can be dated to the Younger Dryas / transition to the Preboreal (Last Glacial Transition). However, this classification is not supported by the vast majority of the ^14^C-ages obtained from bones and individual charcoal pieces (Table 2 and Fig 16). We do not have an explanation for this phenomenon, but suspect post-sedimentary contamination processes to be behind it, a finding that should also have consequences for other comparable sites. Finally, two bone dates and, most worthy of note, ^14^C-dating on silted charcoal spangles from the middle of sediment 6c which yielded an age of approximately 10 kyr cal BC, support the placement of the Late Glacial settlement in the mid/second half of the Younger Dryas (GS-1). This classification is also tendentially confirmed by the luminescence ages obtained from over- and underlying sediments of sediment 6c and–this should be emphasised once again–by the macroscopically undisturbed stratigraphic position of sediment 6c under Holocene cover sediments.

The archaeological find material is characterised by a blade and bladelet industry with numerous variable backed implements and supplemented by an atypical small tanged point and some simple points (Zonhoven points). This GS-1 inventory is unique to our region (Rhineland and Westphalia, western Germany) and the whole of Northwest Europe. Comparable lithic assemblages are only known to have existed in the Younger Dryas from more southern and western regions such as France (here the *Laborien* / *Laborien récent*).

Reindeer, which were typical hunting prey during the GS-1 Ahrensburgian in our and adjacent regions, are–if at all–not prominent amongst the animal finds in front of the *Blätterhöhle*. Inventories of the Ahrensburgian with reindeer remains are documented by scientific dating for both the beginning and the end of the Younger Dryas (although the older dates might need to be checked, see above). The two reindeer remains from the Ahrensburgian of the *Hohler Stein* near Kallenhardt are comparable in their obtained ^14^C-ages [15] to the "sediment date" of the *Blätterhöhle* sediment 6c. The *Hohler Stein* is located just less than 90 km to the east in an identical region (northern edge of the South Westphalian low mountain range; cf. Fig 1). But just how can this been explained?

If our assumptions of a mid-Younger Dryas Final Palaeolithic techno-complex with backed points at the *Blätterhöhle* are correct, this assemblage possibly indicates a short-term immigration of groups from southwestern regions whose lithic industries were attached to the backed point (e.g. Laborian) tradition. This expansionary movement may have been facilitated by a brief north/northeast expansion of their ancestral environment, as indicated by the red deer remains at the *Blatterhöhle*.

**Fig 29:**
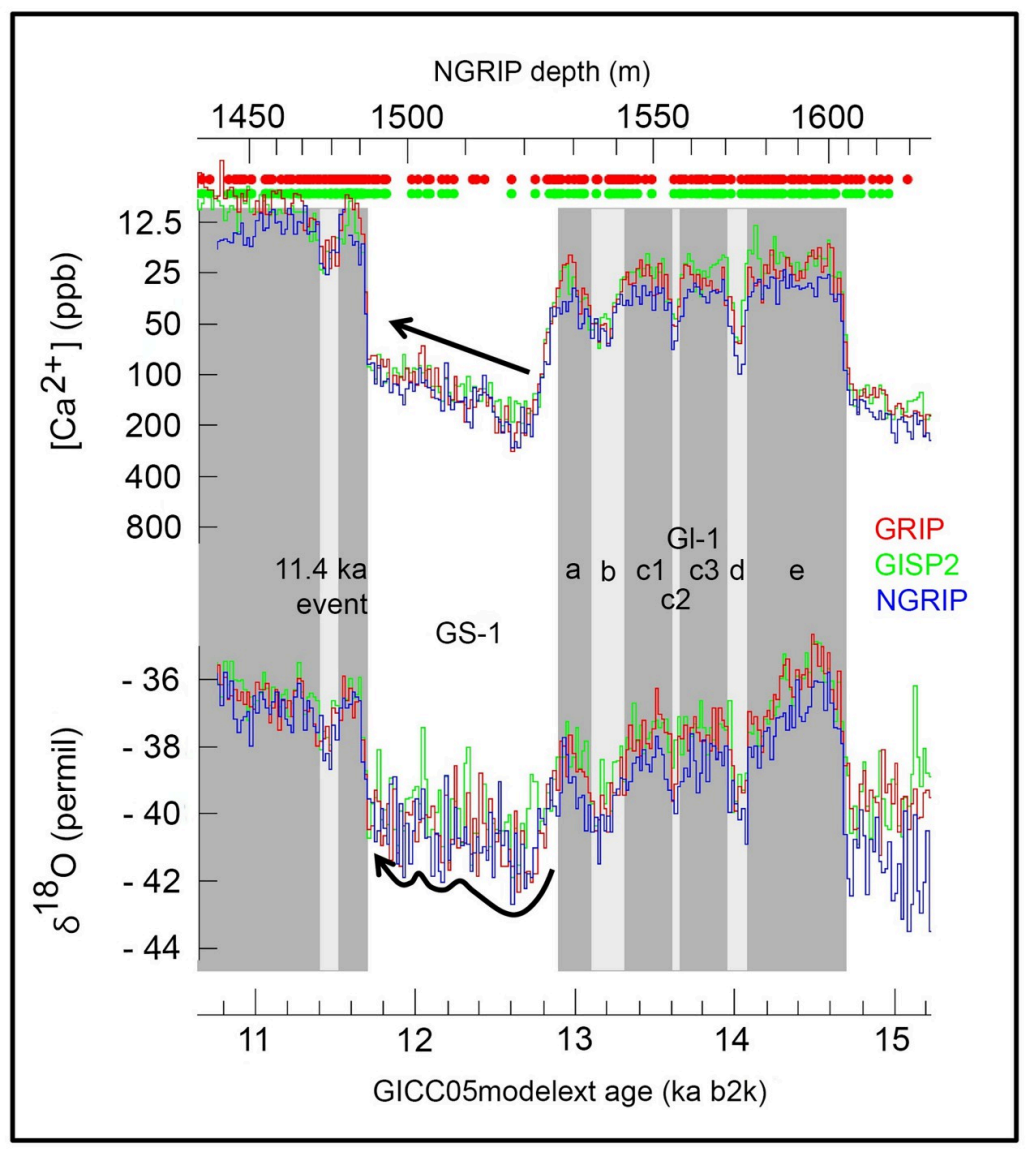
20-year average values of δ^18^O and [Ca^2+^] from the Greenland ice cores GRIP (red), GISP2 (green), and NGRIP (blue) on the GICC05modelext time scale focussing on the 15 to 11 kyr (b2k) time slice. The fluctuations respectively generally positive climate trend for the middle and younger part of GS-1 are clearly visible.–Graphic: LWL-AfW Olpe/M. Baales changed according to [147, Fig 1].

In fact, the climate-relevant proxies, e.g. the Greenland ice cores [cf. 147], do not identify the Younger Dryas / GS-1 as a monolithic climate block. Noticeable fluctuations and deviations towards a very short-term milder climate are recorded here, e.g. clearly around 10 kyr cal BC (Fig 29; cf. Fig 2). Also, the mean δ^18^O-values at the beginning of GS-1 are different from those towards the end of GS-1, which shows a trend towards a slight climate improvement. Futhermore, there are indications of a short-term "warming trend" in northern central Europe for the middle Younger Dryas [148]. Considering the "identical" ^14^C-ages assigned to the reindeer remains of the *Hohler Stein*, we have to assume very short-term and short-lived influences and changes as well as a high dynamic in human behaviour in our region–and also the ability to quickly cover larger distances.

However, it should be pointed out once again that according to the ^14^C-ages, the Ahrensburgian with numerous finds of reindeer has also been proven for the younger section of the Younger Dryas in the northern low mountain range. The ecology, and thus the history of human settlement within the region, seems to have been much more differentiated and sometimes erratic than previously assumed. This suggests a high dynamic of human settlement events and adaptation for our area during the GS-1. However, according to our results in front of the *Blätterhöhle* presented here, the presence of only the Ahrensburgian (and the *long-blade industries*) and the associated hunting of seasonally migrating reindeer herds during GS-1, which lasted for more than a thousand years, should be considered outdated for our region.

Surprisingly, the sparse remains of an approximately 7-year-old child were found within the Final Pleistocene sediments near the entrance to the *Blätterhöhle*: a lower jaw fragment with remains of the dentition and a few dislodged isolated teeth. So far, it has not been possible to clearly explain their presence in the find layer and no other human bones from a burial etc. have come to light in the adjacent excavation areas. We must therefore consider a complex taphonomic history. However, it should be noted that in front of the *Blätterhöhle* for the first time for North Rhine-Westphalia and neighbouring regions informative Pleistocene human remains have been recovered in an undisturbed stratigraphic context [cf. 149]. Moreover, they can be added to the small number of finds of Younger Dryas-era human remains in NW-Europe [cf. 150, 151] (but see [152, p. 78] for a possible reservoir effect affecting the radiocarbon dating of the Rhünda human skull]) and further afield.

Currently, the excavations in the area with the Pleistocene sediments in front of the *Blätterhöhle* have had to be suspended, because before they can be continued, as planned, to the west, overhanging, quite thick Holocene sediments have to be excavated. Consequently, the results obtained so far cannot be verified by further excavation results in near future. However, a further analysis of the 2016–2021 find materials, e.g. the animal remains by means of Zooarchaeology by Mass Spectrometry (ZooMS) etc. is being considered and the micro mammals and other remains of small animals still need to be studied. In addition, the ^14^C-dating problem is awaiting further analysis.

All in all, the situation remains quite exciting.

## Supporting information

S1 File Micromorphological analysis of the sediments / sediment units 6a to 8: Materials and method & dating methods applied to the *Blätterhöhle Vorplatz* used by the Mannheim labs. (PDF)

S1 Table Results of micromorphological analyses. (PDF)

S1 Fig Thin section of BV_20_3 scanned using ordinary transmitted light, reflected light modus or two polarization foils. (TIF)

S2 Fig Thin sections of BV_20_3 and BV_11_7_3 with sediments 8 and 6b *unten*. (TIF)
