# Supplementary material for: Western visitors at the Blätterhöhle (city of Hagen, southern Westphalia) during the Younger Dryas? A new final palaeolithic assemblage type in western Germany
Source: PLoS One. 2023 May 3;18(5):e0284479. doi: 10.1371/journal.pone.0284479 (PMC10156063; doi:10.1371/journal.pone.0284479)
Supplement: S1 File — (PDF) [file pone.0284479.s001.pdf]

Supporting Information

Michael Baales, Wolfgang Heuschen, Martin Kehl, Annika Manz, Nadine Nolde, Daniel Riemenschneider, Holger Rittweger and Jörg Orschiedt

Western visitors at the *Blätterhöhle* (city of Hagen, southern Westphalia) during the Younger Dryas?

A new Final Palaeolithic assemblage type in western Germany

with a contribution by Susanne Lindauer, CEZA Curt-Engelhorn Centre Archaeometry gGmbH
D-68159 Mannheim (Germany)

Micromorphological analysis of the sediments / sediment units 6a to 8

Martin Kehl

Materials and method

The sampling took place at three profiles on the Blätterhöhle *Vorplatz* (cf. Fig 10) in the years 2011 (profile DE, squares D5b/D6b), 2017 (profile DE, square D5d) and 2020 (profile DM, square D5a, and profile of the southern edge of the excavation, square G7b). The allocation of the cuts to the layers and profiles can be found in S1 Table. The monoliths prepared in the field were up to 50 cm long and were fixed with plaster bandages. Having being dried for at least 10 days at 40°C, the blocks were impregnated with polyester resin in a vacuum, cured for approximately six weeks, and then sawn into discs, from which the maximum of 80 mm x 60 mm large and approximately 25-μm-thick sections were created.

Micromorphological analysis was carried out at different scale levels (cf. Macphail & Goldberg 2018). Firstly, flatbed scans of the sections were examined (S1 Fig) on the computer screen at up to 10x to 20x magnification to assess possible compositional and structural heterogeneity. The sections were then analysed with a polarisation microscope at 12.5x to 500x magnification under plane polarised light (PPL), crossed polarisers (XPL) and oblique incident light (RL). The description of the thin sections is in accordance with the terminology suggested by Georges S. Stoops in Stoops, Vepraskas & Jongmans (2003).

The micromorphological analysis of thin sections of sediment aims to examine the size, shape and abundance of the mineral and organic components and provides descriptions of the pore space and the microstructure. Sediment structures resulting from deposition (primary) can clearly be distinguished from those of soil formation (secondary), such as the presence of soil aggregates and characteristics of the accumulation or depletion of mineral or organic matter due to pedogenesis. This makes it possible to disentangle the chronological sequence of deposition and alteration processes. The micromorphological analysis of thin sections has been an important tool in archaeology, prehistory and early history for recording diachronic changes in sediment composition and the natural or anthropogenic accumulation processes (Macphail & Goldberg 2018; Courty, Goldberg & Macphail 1989; Nicosia & Stoops 2017). Through this, a great deal of information can be obtained about the stratigraphy of archaeological sites and also about human behaviour.

An important concept in micromorphology is the distinction made between groundmass and pedofeatures (Stoops, Vepraskas & Jongmans 2003). While the groundmass comprises the composition of the fine or coarse mineral / organic components of the matrix and the pore space, pedofeatures represent discrete structures that are characterised by significant differences in the material composition or the internal spatial structure compared with the surrounding matrix. Pedofeatures result from processes of soil formation (Stoops, Marcelino & Mees 2018).

The coarse constituents (> 2 mm in diametre) consist predominantly of micritic limestone fragments, which in some cases contain sparitic zones or veins where fossils are also often visible. In most cases, these fragments have a triaxial or regular geometry and are rounded on the edges and rarely sharp-edged. Some of the limestone fragments appear somewhat weathered. The majority of the rock fragments comes from the outcropping limestone and were probably released from the rock face by physical weathering before being deposited on the entrance area and overprinted by carbonate dissolution in the sediment. Additionally there are very few quartzite or siltstone fragments found in thin section but, when discovered, they are both well rounded and often spherical in shape**.** This is fine gravel which may well have come from terrace sediments of the Lenne or the Milchenbach.

In addition to the rock fragments mentioned above, there are also coarse components of biogenic origin. Larger chunks as well as small pieces of charcoal are found in most of the thin sections from layers 6a to 6c and are particularly common in thin section BV_20_3 while being absent in the thin sections from sediment 8 (S1 Table). The same applies to broken bones, where the size of the bone fragments is almost always less than 5 mm in edge-length while larger fragments with an edge-length of up to 10 mm only occur rarely. The fragments show the staining characteristic of bone in PPL and XPL. In a few cases, an unusual brown discolouration (PPL) of the bones can be observed, which in at least one case indicates the influence of fire. Bone preservation quality is good to fair. In isolated cases, a clear dissolution of the bone substance can be seen. Finally, very few fragments of snail shells were to be found in certain sections. Remains of recent roots occur down to sediment 8.

The fine fraction (< 2000 to about 5 µm in diameter) is rich in quartz, while calcite and *feldspar* occur to a lesser extent (S2 Fig). Mica platelets, mostly muscovite but also biotite, can be found, too. The siliceous particles are predominantly of the size of silt grains, fine sand is rare. Spherical multicrystalline calcite aggregates of up to approximately 1mm in diameter are present in very small numbers in all of the thin sections. These so-called *calcite biospheroids* can probably be traced back to earthworms (Canti 1998, 2017). The pore space essentially consists of channels and chambers created by soil organisms, while cavities between soil aggregates, which are formed either as irregularly delimited "compound packing voids" or as "planes" characterised by parallel borders, are far less significant in terms of quantity. In some biogenic pores, the remains of recent plant roots, loosely stored mineral grains of the ground mass or small excremental droppings have been found.

Due to the intense bioturbation in places, the microstructure is often prevalent as a "channel and burrow microstructure" or as a spongy microstructure. The formation of a subandgular blocky microstructure can be seen clearly in the sections from sediments 6a and 6b where the average size of the structural elements increases with depth. In sections BV_11_7.1 and 7.2 there are also some sharp-edged (angular blocky) structural elements. The lowest thin section contains biogenic pores, but overall it has a massive microstructure. Some thin sections also show a slightly distinguishable platy structure and often different structural forms can be found in one and the same sample. As already observed in the field, generally the thin sections reflect the loosely packed to moderately dense storage of the sediments.

The ‘micromass’ that cannot be separated by light microscopy (i.e. particles smaller than 5 µm in diameter) is light brown in sediments 6a and 6b, grey in sediment 6c and light-yellow / grey in sediment 8. A few spots that appear opaque under PPL are mostly reddish brown in OIL, which indicates the presence of Fe hydroxides that were formed during the browning process. In addition, small black stains can also be found under OIL, and these can be traced back to an accumulation of organic matter (*humus*), fine charcoal pieces or manganese oxides.

In the sections from profile DE, the sediments 6a and 6b show stipple speckled or mosaic speckled birefringence fabric (b-fabric), which indicate the formation of clay domains arising after complete decalcification of the groundmass by chemical silicate weathering, e.g. from biotites (e.g. Stephan 2000). This also applies to the two thin sections from the profile in square G7b. In contrast, both thin sections from profile DM show a crystallitic b-fabric, which is caused by fine calcite grains within the fine substance. Crystallitic b-fabric is also present in the sections from the lowest part of sediment 8 (S2 Fig).

The pedofeatures, which can be traced back to soil formation processes, are manifold. Calcite depletion zones occur in sections BV17_1 to 17_2_2 (sediment unit 6b *unten*; S2 Fig), while they are completely absent in the lower profile area. The thin sections with speckled b-fabric mentioned above are completely decalcified and in addition to the evidence of the depletion of primary carbonate, numerous precipitations of secondary carbonate can be found. These are predominantly micritic, partly microsparitic calcite crystals that occur as hypocoatings, nodules or as incomplete infillings. Besides calcitic pedofeatures, very thin patchy clay coatings occur along the pore walls (S2 Fig) and this indicates the beginning of clay mobilisation. In thin section BV_20_3 from sediment 6c and in two sections from sediment 8 there are also silt cappings on the upper side of coarse rock fragments. Finally, very few typical Fe/Mn concretions occur and this indicates a very weak hydromorphic overprint by stagnic water.

Accumulations of phosphate were only very occasionally observed in the form of coprolites. No clear indications of fine stratification or the presence of ash layers or ash aggregates were evident from the analysis.

The sorting of the fine substance suggests that a high proportion of dust grains were added to the slope sediments during the loess accumulation phases of the last glacial period and then, through the process of relocation, reached the Holocene layers.

S1 & S2 Figure captions

S1 Fig. Thin section BV_20_3 scanned using ordinary transmitted light (left), reflected light modus (center) or two polarization foils (right). The dashed line marks the boundary between sediment 8 at the bottom and sediment 6c at the top. Note that sediment 6c has a grey colour at the base, while it grades into brown at the top (Scale is 10 mm). – Photos: University of Cologne/M. Kehl.

S2 Fig. The groundmass of sediment 8 is rich in primary grains of carbonate (Ca) and has a crystallitic b-fabric (top, thin section Bv_20_3). Several samples from sediment unit 6b *unten* (bottom, here thin section BV_11_7_3) show complete carbonate leaching, have a stipple to mosaic speckled b-fabric and contain thin impure clay coatings (Cc; scales are 200 µm). – Photos: University of Cologne/M. Kehl.

Dating methods applied to the Blätterhöhle *Vorplatz*

Susanne Lindauer

The chronological context of the Final Pleistocene sediments in front of the Blatterhöhle was determined using two dating methods, optically stimulated luminescence (OSL) for the sediment layers and radiocarbon dating for the organic contents found throughout the stratigraphic context. Both dating methods are provided by the Curt-Engelhorn Centre Archaeometry gGmbH (CEZA) in Mannheim, Germany.

Luminescence dating

Sediment samples for OSL dating were taken using light- and water proof plastic cylinders. In the dark laboratory at CEZA, at each end approximately 2 cm of material were removed as this material had seen light during sampling. This material was used to determine the water content of the samples and, when dried, used to determine the radioactivity of the samples via gammaspectrometry (high purity Ge-crystal, well detector, Canberra, now Mirion). Here, Thorium and uranium together with their daughter nuclides as well as potassium are measured to determine the doserate, i.e. radioactivity per time. The water content is measured as water absorbs radiation so that not all radioactivity reaches the mineral grains and contributed to the OSL signal build-up. The OSL sample is prepared by sieving to different grain sizes: <100 µm which is used for fine grain, 100 - 200 µm which provides the coarse grain fraction and >200 µm which is not used. In a next step, carbonates and organic remains are removed by using perdrogen (H_2O_2) and acetic acid. For polymineral fine grains, the grainsize is then further reduced to 4 - 11 µm by settling the grains in acetone for different time according to Stoke´s law. In the coarse grain fraction, grains are heavy enough to be separated into different minerals using heavy liquid (lithiumploytungstate). Because the coarse grain fraction is too big for alpha particles to complete travel through them, the contribution of alpha radiation cannot be reliably reconstructed due to the resulting inhomogeneous radiation field. Therefore, grains of this size are then etched with hydrofluoric acid to remove the outer, alpha-influenced, 20 µm (Lang et al. 1996).

After sample preparation, the samples are brought on discs and measured in a TL-DA-20 Reader (Riso). OSL quartz samples are measured according to (Murray & Wintle 2000). Quartz is stimulated using blue LEDs and the OSL detected in the ultraviolett (filter U340, Schott). Here, one tries to reconstruct the natural dose of the sample by several irradiation/measurement cycles with increasing dose and constant test dose irradiation/measurements between each irradiation cycle. To remove short-lived signals that occur directly after irradiation, a preheat step is done between each irradiation and measurement. The ideal preheat temperature is determined by a preheat test measurement beforehand. For feldspar measurements, the mineral grains are stimulated using infrared LEDs and detect the OSL signal in the blue wavelength range (filter BG3, BG39, Schott). Feldspar IRSL (infrared stimulated luminescence) is measured at several temperatures to find the signal that is most stable over time because feldspar can show fading (Von Suchodoletz et al. 2022). This is a tunneling effect that leads to signal loss over time without stimulation and leads to age underestimation.

The data is then analyzed with respect to scatter of the data points, determination of the dose using a central age model (CAM), if the data shows few scatter, or a minimum age model (MAM), if large scatter occurs. Both methods use all datapoints chosen, but the MAM puts more weights on the low data points that represent younger ages assuming partial bleach. This is an effect when the sediment accumulated under bad light conditions resulting in some grains not being bleached in the settlement process. As quartz bleached faster than feldspar, measuring the feldspar in addition can also give hints towards partial bleach when the feldspar ages are older. Different ages for the different grain sizes on the other hand might provide insight into settlement processes where fine grain and coarse grain contributed to this sediment layer from different processes.

AMS-Radiocarbon dating

The radiocarbon (^{14}C) samples do not date the sediment directly as radiocarbon is dating the event when a sample incorporates the carbon from the atmosphere or another reservoir. However, still radiocarbon dates on samples in sediment layers can be contemporaneous, especially when dealing with short-lived samples such as seeds. Charcoal samples on the other hand date the event when the tree rings being part of the charcoal incorporated the ^{14}C into their ring, but not the burning event, unless the sample really includes the very last ring before felling. When using bones for radiocarbon dating, usually collagen is extracted as this does not exchange carbon with its surrounding such as limestone in the sediment. Dating bulk sediment provides a certain challenge as it is difficult to determine on which material exactly the age is measured.

The sample preparation for radiocarbon dating aims at removing unwanted carbon, such as later induced humic acids, limestone etc. from the fraction to be used. This usually starts with an acid step (diluted hydrochloric acid, HCl) to remove carbonates (in the case of shells, secondary carbonates or surface contamination), followed by a base step with diluted sodium hydroxide (NaOH) to remove soluble humic acids. As NaOH collects fresh CO2 from the atmosphere, another acid step is needed to remove the modern contamination again. This is in short the sample preparation for charcoal and bulk sediment (Lindauer et al. 2015). The bone pretreatment is slightly more elaborate and starts in the same way with acid-base-acid, followed by a gelatinization step using HCl at pH3 for 20 hours at 60°C. The liquid collagen is then filter through an ultrafilter to separate heavier, intact molecules from broken pieces and foreign collagen (Lindauer et al. 2015). After pretreatment, samples are converted to elemental carbon (“graphitized”). Samples are combusted in an elemental analyzer (MicroCube, elementar) and the resulting CO_2 catalytically reduced (“graphitized) to C with iron as catalyst. The iron-C mixture is pressend into a target and measured in a MICADAS AMS system (Kromer et al. 2013). The data is fractionation corrected using the $\delta^{13}C$ data from the AMS measurement and calibrated using OxCal v4.4.4 with the IntCal20 dataset.

S1 References

Canti M. Origin of Calcium carbonate granules found in buried soils and Quaternary deposits. Boreas 1998; 27: 275–288.

Canti, MG. Biospheroids Produced by Earthworms. In: Nicosia C, Stoops GS, editors. Archaeological Soil and Sediment Micromorphology. Chichester/UK: Wiley; 2017. pp. 47–49.

Courty MA, Goldberg P, Macphail RI. Soils and micromorphology in archaeology. Cambridge/UK: Cambridge University Press; 1989.

Kromer B, Lindauer S, Synal H-A, Wacker L. MAMS – A new AMS facility at the Curt-Engelhorn-Centre for Achaeometry, Mannheim, Germany. Nuclear Instruments and Methods in Physics Research Section B: Beam Interactions with Materials and Atoms 2013; 294: 11–13.

Lang A, Lindauer S, Kuhn R, Wagner GA. Procedures Used for optically and Infrared Stimulated Luminescence Dating of Sediments in Heidelberg. Ancient TL 1996; 14(3): 7–11.

Lindauer S, Tomasto-Cagigao E, Fehren-Schmitz L. The skeletons of Lauricocha: New data on old bones. J Archaeol Sc: Rep. 2015; 4: 387–394.

Macphail RI, Goldberg P. Applied soil and Micromorphology in Archaeology. Cambridge Manuals in Archaeology. Cambridge/UK: Cambridge University Press; 2018.

Murray AS, Wintle AG. Luminescence dating of quartz using an improved single-aliquot regenerative-dose protocol. Radiation Measurements 2000; 32: 57–73.

Nicosia C, Stoops GS, editors. Archaeological Soil and Sediment Micromorphology. Chichester/UK: Wiley; 2017.

Stephan S. Bt-Horizonte als Interglazial-Zeiger in den humiden Mittelbreiten: Bildung, Mikromorphologie, Kriterien. Eiszeitalter und Gegenwart 2000; 50: 95–106.

Stoops G, Marcelino V, Mees F, editors. Interpretation of Micromorphological Features of Soils and Regoliths. 2nd ed. Dordrecht: Elsevier; 2018.

Stoops GS, Vepraskas MJ, Jongmans AG, editors. Guidelines for Analysis and description of soil and regolith thin sections. Madison/Wi.: Soil Science Society of America; 2003.

Von Suchodoletz H, Kirkitadze G, Koff T, Fischer ML, Poch RM, Khosravichenar A, et al. Human-environmental interactions and seismic activity in a Late Bronze to Early Iron Age settlement center in the southeastern Caucasus. Front. Earth Sci. 2022; 10: 964188. doi: 10.3389/feart.2022.964188.
