# Supplementary material for: Western visitors at the Blätterhöhle (city of Hagen, southern Westphalia) during the Younger Dryas? A new final palaeolithic assemblage type in western Germany
Source: PLoS One. 2023 May 3;18(5):e0284479. doi: 10.1371/journal.pone.0284479 (PMC10156063; doi:10.1371/journal.pone.0284479)
Supplement: S1 Table — (PDF) [file pone.0284479.s002.pdf]

S1 Table. Results of micromorphological analyses. Key: Abundance rating is from 0 = not found to 5 = very abundant (20 - 30%). B-fabric types: cr = crystallitic; ssp = stipple speckled; msp = mosaic speckled; Microstructure types: cha = channel; chb = chamber; cru = crumb; ma = massive; pla = platy; spo = spongy; sub bl or ang bl = subangular or angular blocky; dgm = depleted groundmass.

**Thin section**	**Layer**	**Profile**	**Charcoal**	**Bone**	**Fresh roots**	**Calcite biosp.**	**Microstructure**	**b-fabric**	**Carbonate depletion**	**Calcite hypocoat.**	**Calcite nodules**	**Impure clay coats**	**Silt cappings**
BV_11_6_3	6a	P2 D6b	1	1	1	1	sub bl, cha, chb	ssp	dgm	3	2	2	0
BV_11_6_4	6b	P2 D6b	1	1	1	1	pla, sub bl, cha	ssp (msp)	dgm	3	3	2	0
BV_11_7_1	6a/6b	P2 D6b+D5d	1	1	1	1	sub to ang bl, pla	ssp (msp)	dgm	3	1	2	0
BV_11_7_2	6b	P2 D6b+D5d	1	1	2	1	sub to ang bl, cha, chb	ssp (msp)	dgm	3	1	2	0
BV_11_7_3	6b	P2 D6b+D5d	1	1	0	1	sub bl, cha, chb	ssp (msp)	dgm	3	1	1	0
BV_17_1	6a/6b	P5 D5d	1	1	1	1	spo, cru, fine sub bl	cr (ssp)	2	3	1	2	0
BV_17_2_1	6b	P5 D5d	1	1	1	1	spo, cru, fine sub bl	cr (ssp)	1	3	1	1	0
BV_17_2_2	6b	P5 D5d	0	1	1	1	fine sub bl, cru, spo	cr (ssp)	2	3	1	0	0
BV_17_3_1	6b/TG	P5 D5d	0	0	1	1	fine sub bl, cru, spo	cr	0	4	1	0	0
BV_17_3_2	6c	P5 D5d	1	1	0	1	cha, chb, sub bl	cr	0	5	2	0	0
BV_17_3_2	8	P5 D5d	0	0	0	1	cha, sub bl, ma	cr	0	4	1	0	0
BV_17_3_3	8	P5 D5d	0	0	1	1	sub bl, pla, ma	cr	0	4	1	0	1
BV_20_1	6b	G7a	1	1	1	1	vug, sub bl, mas, pla	ssp, msp	dgm	4	2	2	0
BV_20_2	6b	G7a	0	1	1	1	spo	ssp (cr)	dgm	3	1	1	0
BV_20_3	6c	P5 D5a	2	2	0	1	cha, spo	cr	0	5	2	0	2
BV_20_3	8	P5 D5a	0	0	0	1	ma, cha	cr	0	4	2	0	1
