# Supplementary figures and images for: Western visitors at the Blätterhöhle (city of Hagen, southern Westphalia) during the Younger Dryas? A new final palaeolithic assemblage type in western Germany
Source: PLoS One. 2023 May 3;18(5):e0284479. doi: 10.1371/journal.pone.0284479 (PMC10156063; doi:10.1371/journal.pone.0284479)

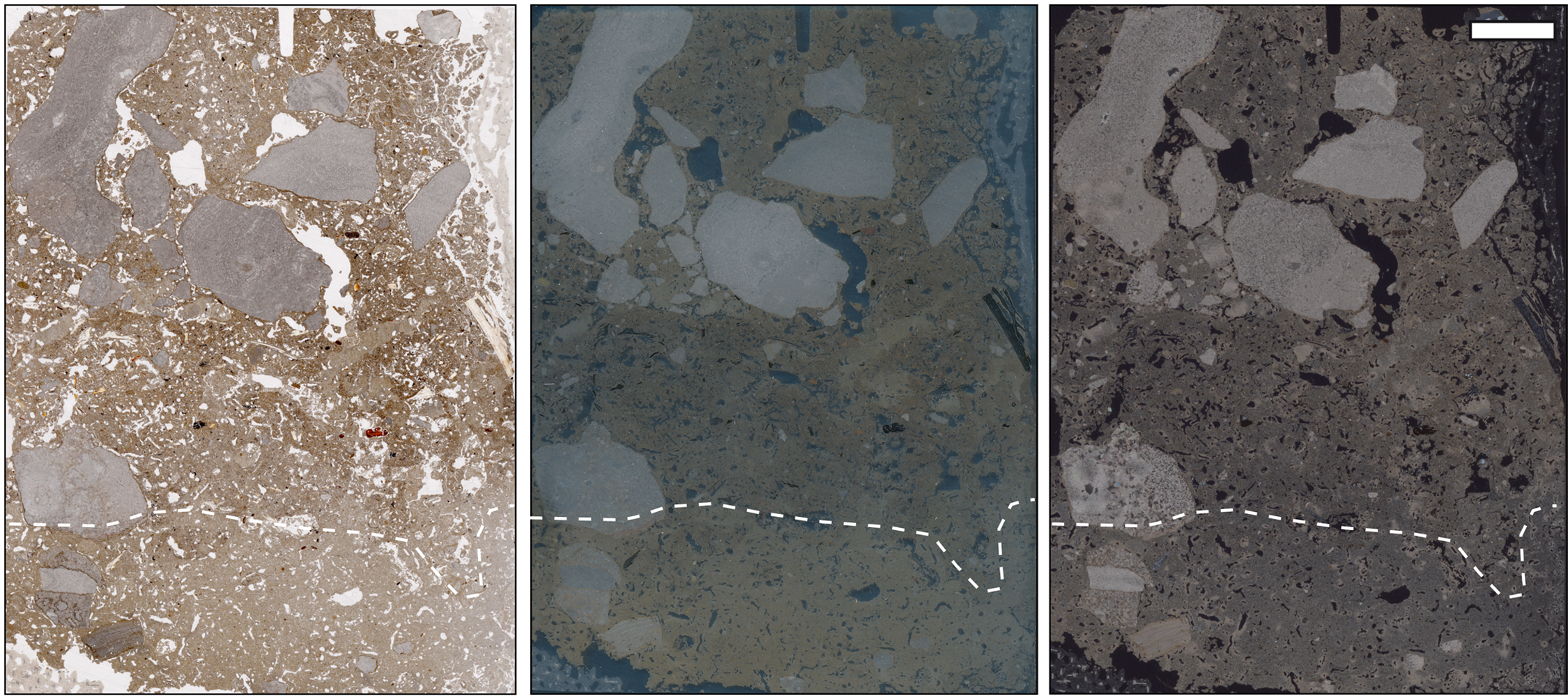

Supplement: S1 Fig — (TIF) [file pone.0284479.s003.tif]

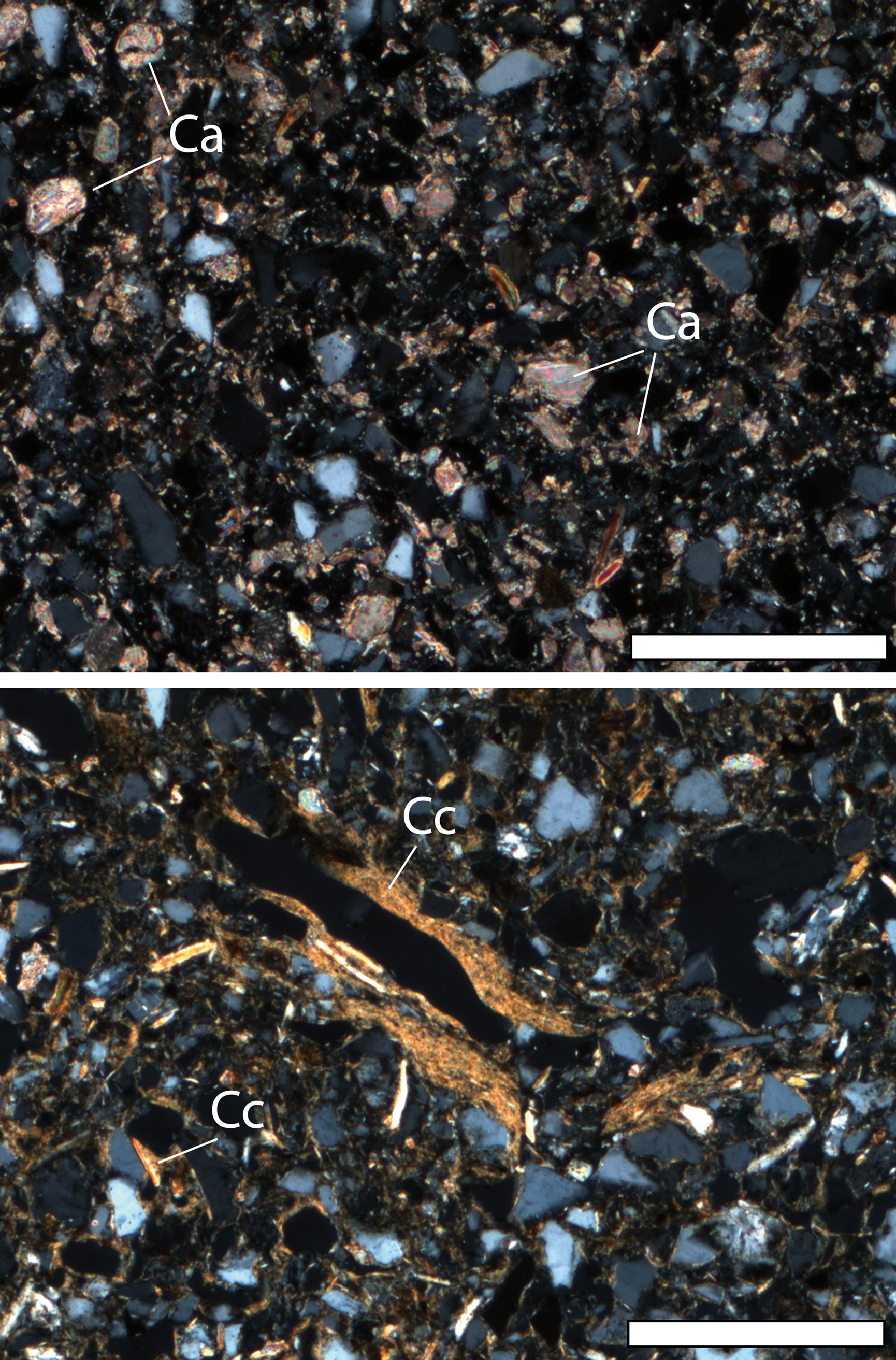

Supplement: S2 Fig — (TIF) [file pone.0284479.s004.tif]
